# The role of HDAC2 inhibition in cardioprotection against doxorubicin-induced myocardial injury

**DOI:** 10.3389/fcvm.2025.1557119

**Published:** 2025-02-26

**Authors:** Jinsha Liu, Wenwen Fu, Xue Wang, Zuowen Liang, Fanbo Meng

**Affiliations:** ^1^ Department of Cardiology, China-Japan Union Hospital of Jilin University, Changchun, China; ^2^ Jilin Provincial Engineering Laboratory for Endothelial Function and Genetic Diagnosis of Cardiovascular Disease, Jilin Provincial Cardiovascular Research Center, Changchun, China; ^3^ Department of Pharmacology, School of Pharmaceutical Sciences, Jilin University, Changchun, China; ^4^ Department of Andrology, The First Hospital of Jilin University, Changchun, China

**Keywords:** HDAC2, doxorubicin, cardiac function, PI3K/AKT, apoptosis

## Abstract

**Introduction:**

The molecular mechanisms underlying cardioprotection against doxorubicin (DOX)-induced myocardial injury are poorly understood. Histone deacetylase 2 (HDAC2) plays a significant role in oxidative stress, apoptosis, and mitochondrial dysfunction and is implicated in many human diseases, This study investigated the relationship between HDAC2 expression and DOX-induced myocardial injury using the *in vivo* rat model of DOX-induced cardiotoxicity and *in vitro* experiments with the H9c2 cardiomyocytes.

**Methods:**

The rat model of DOX-induced myocardial injury was established by administering DOX via intraperitoneal injections. HDAC2 expression was suppressed by administering rats with sodium butyrate (SB) via intraperitoneal injections. Echocardiography measurements were performed at baseline and on day 15 post-treatment. The rats were euthanized on day 15 and cardiac tissues were harvested. The cardiac tissue samples were analyzed by hematoxylin and eosin H&E staining, immunohistochemistry, Masson staining, Sirius Red staining, TUNEL staining, and western blotting to determine the status of HDAC2 expression and myocardial apoptosis. In the vitro experiments, H9c2 cells were treated with DOX. HDAC2 expression was suppressed using sodium butyrate or transfected cells with the shRNA knockdown HDAC2 (shHDAC2). The H9c2 cells from different groups were analyzed by Rt-qPCR, CCK-8 cell viability assay, and western blotting to determine the status of HDAC2 expression and cardiomyocyte apoptosis.

**Results:**

DOX treatment induced cardiac dysfunction in rats. The cardiac tissues of the DOX-treated rats and H9c2 cells showed significantly higher levels of HDAC2 compared to the corresponding controls. However, inhibition of HDAC2 significantly mitigated DOX-induced myocardial injury in rats. This suggested a strong association between HDAC2 expression and DOX-induced myocardial injury. In the H9c2 cells, HDAC2 knockdown by shHDAC2 alleviated DOX-induced apoptosis by enhacing AKT phosphorylation. These findings demonstrated that HDAC2 silencing protected against DOX-induced cardiomyocyte apoptosis by activating the PI3K/AKT signaling pathway.

**Conclusion:**

Suppressing HDAC2 protected against DOX-induced cardiomyocyte apoptosis by activating the PI3K/AKT signaling pathway. Therefore, HDAC2 is a promising therapeutic target for mitigating DOX-induced myocardial injury.

## 1 Introduction

In the last few decades, advances in medicine and medical technology have significantly increased life expectancy (1). Cardiovascular disease (CVD) and cancer have emerged as two major health challenges worldwide in the aging population (1–3). The development of diverse and effective cancer treatment modalities have improved the survival outcomes of cancer patients. However many chemotherapeutic drugs cause myocardial injury. Therefore, understanding and managing the cardiotoxic effects of chemotherapy has gained the attention of researchers in the fields of oncology and cardiology.

**Table 1:** Echocardiography measurements of rats in different groups at baseline and day 15.

Data	Group							
	Baseline				Day 15			
	CTR	DOX	DB	SB	CTR	DOX	DB	SB
LVIDd (cm)	0.6	0.6	0.6	0.7	0.7	0.7	0.7	0.6
	0.8	0.6	0.6	0.6	0.6	0.7	0.6	0.8
	0.7	0.7	0.7	0.5	0.6	0.9	0.5	0.6
	0.6	0.7	0.7	0.6	0.7	0.6	0.6	0.6
	0.8	0.5	0.6	0.8	0.5	0.7	0.5	0.7
	0.8	0.5	0.7	0.6	0.5	0.5	0.5	0.7
LVIDs (cm)	0.3	0.3	0.3	0.3	0.3	0.5	0.4	0.3
	0.4	0.3	0.3	0.3	0.3	0.5	0.3	0.4
	0.4	0.4	0.3	0.3	0.3	0.6	0.3	0.3
	0.3	0.4	0.4	0.3	0.3	0.4	0.4	0.3
	0.4	0.3	0.3	0.4	0.3	0.5	0.2	0.3
	0.4	0.2	0.3	0.3	0.2	0.4	0.3	0.3
EF (%)	83	85	83	85	85	64	81	84
	80	85	84	85	86	65	83	88
	80	81	85	82	86	63	83	83
	85	87	84	85	85	67	77	86
	85	84	87	83	87	64	85	84
	84	86	87	86	88	65	87	85
FS (%)	47	49	46	48	49	31	44	48
	43	48	48	49	50	31	47	53
	44	45	49	45	50	30	46	47
	49	50	47	50	49	32	41	50
	49	47	51	47	51	30	49	48
	48	50	51	50	53	31	51	49
HR (BPM)	254	312	386	353	285	477	353	345
	353	377	290	301	280	369	312	254
	369	386	369	369	325	338	361	427
	331	353	338	427	254	290	439	439
	416	464	338	338	325	369	427	406
	338	492	325	477	507	353	439	325

LVIDd, left ventricular end-diastolic diameter; LVIDs, left ventricular end-systolic diameter; EF%, ejection fraction; FS%, fractional shortening; HR, heart rate. CTR, control; DOX, Doxorubicin; DB, Doxorubicin + SB; SB, control + sodium butyrate.

Doxorubicin (DOX), a well-established anthracycline chemotherapeutic agent, is widely used for the treatment of pediatric cancers, leukemias, and breast cancer. However, its clinical application is significantly limited because of significant adverse effects, especially DOX-induced myocardial injury (4, 5). DOX-induced myocardial injury can lead to heart failure and ultimately result in death (6). Strategies such as low-dose regimens (7), continuous and prolonged infusion schedules (8), administration of cardioprotective agents, development of novel anthracycline analogs (9), and innovative formulations such as liposomes (10) and nanoformulations (11) have been tested in the preclinical studies to overcome the cardiotoxic effects and enhance the therapeutic efficacy of DOX, but have shown suboptimal results (12–14). Currently, there is no definitive method or consensus method to effectively protect against DOX-induced myocardial injury. Furthermore, celluar-mechanisms that protect cardiomyocytes against the toxic effects of DOX-induced myocardial injury has not been.

HDAC2, a member of the class I histone deacetylases (HDACs), is associated with poor prognosis across various tumors (15–17). HDAC2also linked with myocardial fibrosis (18), atrial fibrillation (19) and cardiac hypertrophy (20). HDAC2 plays as significant role in tumorigenesis and heart diseases by regulating oxidative stress, cell apoptosis, and mitochondrial dysfunction (21–25). Sodium butyrate (SB), a short-chain fatty acid (SCFA), is a well-known HDAC2 inhibitor (26). In this study, we established both *in vitro* and *in vivo* models of DOX-induced myocardial injury and investigated whether SB effectively suppressed HDAC2 expression. Since SB is not a specific inhibitor of HDAC2, we performed HDAC2 knockdown via transfection *in vitro*. The PI3 K/AKT signaling axis regulates several critical cellular processes, including cell growth, metabolism, and survival (27). In this study, we investigated HDAC2 was as a novel therapeutic target for DOX-induced myocardial injury using the rat model of DOX-induced myocardial injury and the *in vitro* model of DOX-induced cardiomyocyte death.

## 2 Materials and methods

### 2.1 Animals

Adult male Sprague Dawley rats (200–220 g, 6–8 weeks old) were obtained from Jiutai District Tumenling Integrity Animal Distribution Office Co., Ltd. All the rats were housed in the Animal Center of the Jilin University School of Pharmaceutical Sciences at a constant temperature of 25 ± 1°C, 40%–70% humidity, 12/12 h light-dark cycle, and constant air exchange. The rats had free access to water and food. The study was reported according to the ARRIVE guidelines.

Sodium butyrate (SB) was purchased from Sigma-Aldrich (Cat. No. 303410, Germany). Doxorubicin was purchased from APExBIO (Cat. No. A1832, APExBIO Technology LLC) that Sprague Dawley rats were randomly divided into the following four groups: (1) control group; (2) control + SB (SB) group; (3) DOX group; (4) DOX + SB (DB) group (*n* = 6 per group). The cumulative dose of DOX was 15 mg/kg. Briefly, DOX was administered into rats via intraperitoneal injections at a dose of 2.5 mg/kg on day 2, 4, 6, 8, 10, 12 and 14. The rats in the control group were administered saline 5 ml/kg via intraperitoneal injections at the same frequency as DOX. The dose of SB was 300 mg/kg and was administered via intraperitoneal injection every day. All the rats were euthanized on day 15. Then the hearts were harvested and stored in liquid nitrogen for further experiments or fixed with 4% paraformaldehyde for histological analysis.

The animal protocols used in this study were approved by the Institutional Animal Care and Use Committee of the Jilin University of Pharmaceutical Sciences (Project No. is 20230085).

### 2.2 Echocardiography

Echocardiography was performed at baseline and on day 15 using a GE Vivid-i probe (10S; 4.0–11.0 MHz) to evaluate the the rats cardiac function of the rats in different groups. The rats were anesthetized using 100% oxygen with 1.5%–2% isofluraneusing. We measured left ventricular end-diastolic diameter (LVIDd), left ventricular end-systolic diameter (LVIDs), ejection fraction (EF%) and fractional shortening (FS%). The staff performing the echocardiography weas blinded to the treatment groups.

### 2.3 H&E staining

The cardiac tissue was fixed in 4% paraformaldehyde at 4°C for at least 24 h, then, it was embedded in paraffin and sectioned. The slices were then processed in a stepwise manner as follows: xylene Ⅰ (Sinopharm Chemical Reagent Co., Ltd. Cat. No. 10023418) for 15min-xylene Ⅱ for 15 min-absolute ethanol Ⅰ (Sinopharm Chemical Reagent Co., Ltd. Cat. No. 100092008) for 5 min-absolute ethanol Ⅱ for 5 min-absolute ethanol for 5 min in sequence, and washed. The sections were stained with hematoxylin (Beijing Solarbio Science & Technology Co., Ltd. Cat. No. G1120) dye for 3–5 min and washed with distilled water. The sections were then incubated with differentiation solution and washed. Then, the sections were incubated with the blue solution followed by washing. The sections were dehydrated in 85% and 95% alcohol solutions for 5 min each. Subsequently, the sections were then processed as follows: stained in the eosin staining solution for 5 min. The sections were placed in absolute ethanol Ⅰ for 5 min-absolute ethanol Ⅱ for 5 min-absolute ethanol Ⅲ for 5 min- ylene Ⅰ for 5 min-xylene Ⅱ for 5 min. Sealed with neutral gum (Sinopharm Chemical Reagent Co., Ltd. Cat. No.10004160), and photographed using the (Nikon Eclipse E100; DS-U3).

### 2.4 Immunohistochemistry

Cardiac tissues were fixed in 4% paraformaldehyde at 4°C for at least 24 h. Then they were embedded in paraffin and sectioned. The tissue slices were then processed as follows: three incubations with xylene Ⅰ, Ⅱ, Ⅲ for 15 min each; absolute ethanol Ⅰ, Ⅱ for 5 min each; 85% alcohol for 5 min; 75% alcohol for 5 min; washed with distilled water. The sections were incubated in a repair box filled with the antigen retrieval buffer. Subsequently, antigen retrieval was performed by boiling for 8 min at medium heat in a microwave oven followed by medium-low heat for 7 min. After cooling, the sections were incubated in PBS buffer (Sangon Biotech Co., Ltd. Cat. No. B548117) and shaken on a destaining shaker thrice for 5 min each. Then, the sections were incubated in the dark at room temperature for 25 min in 30% hydrogen peroxide solution (Sinopharm Chemical Reagent Co., Ltd. Cat. No. 10011208). The sections were then washed in PBS buffer three times while shaking on a destaining shaker for 5 min each. Then, the sections were blocked with 3% BSA and sealed at room temperature for 30 min. Subsequently, the sections were incubated with the primary antibody overnight at 4°C. After washing, they were incubated with the secondary antibody at room temperature for 50 min. Then, the sections were incubated with DAB for color development. The nuclei were counterstained. The sections were dehydrated with graded series of alcohol and sealed. The images were captured using a Nikon E100 light microscope.

### 2.5 Masson staining

The tissues were fixed in 4% paraformaldehyde at 4°C for at least 24 h. Then there were embedded in paraffin, sectioned, and dehydrated through graded alcohol series. The sections were then soaked in Masson C solution at room temperature overnight. Subsequently, the sections soaked in Masson C solution were incubated at a 65°C in an oven for 30 min, They were then washed in distilled water for 30 s until the yellow color faded from the tissue. At the same time, we pre-heated the Masson D and Masson F solutions at 65°C in an oven. We then mixed equal volumes of Masson A and Masson B solutions. The sections were incubated in the mixed Masson A + B solution mixture for 1 min and then washed. The sections were differentiated with 1% hydrochloric acid alcohol for about 1 min until the cell nuclei turned grayish-black and the background was almost colorless or light gray. The sections were washed briefly, and excess water was drained. The sections were soaked in the Masson D solution for 6 min. Then, after draining the sections, they were soaked in the Masson E solution for about 1 min. After draining the excess Masson E, solution the sections were directly stained in Masson the F solution for 2–30 s without washing. The slices were rinsed and differentiated in three consecutive tanks of 1% glacial acetic acid thrice for about 8 s each. Then, they were serially dehydrated in a absolute ethanol for 5 s, 10 s, and 30 s. each. Then, they were serially dehydrated in n-butanol for 30 s and 2 min. The sections were finally cleared by incubation with xylene twice for 5 min each. The sections were then sealed sealed and photographed using the Nikon Eclipse E100 DS-U3a microscope.

### 2.6 Sirius Red staining

The cardiac tissue slices were processed as follows: incubation with in xylene Ⅰ, Ⅱ for 20 min each; incubation with absolute ethanol Ⅰ, Ⅱ for 5 min each; incubation with 75% alcohol for 5 min, wash with distilled water. The sections were incubated in the Sirius Red staining solution for 8–10 min. Then, they were and dehydrated twice or thrice with absolute ethanol. The slices were cleared with xylene for 5 min and then sealed and photographed with the Nikon Eclipse E100; DS—U3 light microscope camera.

### 2.7 TUNEL staining

The cardiac tissue slices were processed as follows: incubation with xylene Ⅰ, Ⅱ for 15 min each; incubation with absolute ethanol Ⅰ, Ⅱ for 5 min each; incubation with 85% alcohol, 75% alcohol for 5 min each. Then, wash with distilled water. After dying the slices slightly, a histochemistry pen was used to draw a circle around the tissue to prevent the liquid from flowing away. Then proteinase K working solution was added dropwise, and the tissues were the tissue at a 37°C for 30 min. Subsequently, the sections were incubated in PBS with constant shaking thrice for 5 min each. The slices were slightly dried followed by incubation with the membrane permeabilization solution at room temperature for 20 min. Then, they were washed in PBS on a shaker on a shaker 3 times for 5 min each. Subsequently, we mixed reagent 1 (TdT) and reagent 2 (dUTP) from the Tunnel staining kit in a 2: 29 ratio. The tissue sections were then incubated with this mixture in a humidified box at 37°C for 2 h. The sections were washed three times with PBS for 5 min each. After removing PBS, the nuclei were stained with the DAPI dye solution at room temperature for 10 min in the dark. The slides were then washed thrice with in PBS by constant shaking for 5 min each. After drying the slides briefly, the sections were mounted them with the anti-fluorescence quenching mounting medium. The stained sections were analyzed and photographed using the Nikon ECLIPSE C1 DS-U3 fluorescence microscope.

### 2.8 Cell culture

The H9c2 cell line was purchased from iCell Bioscience (Shanghai, China) and cultured in Dulbecco's modified Eagle's medium (DMEM) supplemented with 10% fetal bovine serum (FBS). 4,500 mg/L glucose (Viva Cell Biosciences, Shanghai, China) and penicillin/streptomycin solution (Solarbio, Beijing, China) at 37°C and 5% CO_2_ in a humidified incubator. For the experiments, H9c2 cells after stable 2–3 passages and less than 20 passages were used. They were seeded in a 10 cm petridish at a density of 5∼6 × 10^6^.

### 2.9 CCK-8 cell viability assay

H9c2 cells were cultured in a 96-well plate for 24 h and cell viability was measured using the Cell Counting Kit-8 (CCK-8) assay (Beyotime, Jiangsu, China) according to the manufacturer's instructions. The cells were treated with different concentrations of DOX (0–5 µmol/L), DOX + SB and SB for 24 h. The concentration of SB was 2.5 mmol/L. Then, the cells were incubated with the CCK-8 solutions at 37°C for 1 h. The absorbance was then measured at 450 nm with a microplate reader.

### 2.10 Cell experiments

H9c2 cells were cultured with DOX at 37°C for 24 h to generate a model of apoptosis. SB is an inhibitor of HDAC2 that can inhibit the expression of HDAC2. SB was added to the cells with DOX coculture for 24 h. The concentration of the DOX concentration was 5 *µ*mol/L, and the SB concentration was 2.5 mmol/L.

### 2.11 Cell transfections

H9c2 cells were seeded in 6-well plates at a density of 5 × 10^5^ cells/well. When the cells reached 60%–70% confluence, they were transfected with the shHDAC2 plasmids (Gene Pharma Co., Ltd., Suzhou, China) using the GP-transfect-Mate. Serum-free Opti-MEM was mixed well with 6 µl of the shHDAC2 plasmids (7.5 µl of shHDAC2 at a concentration of 20 pmol/µl) for 5 min at room temperature. After 20 min, cells were mixed with the plasmid mixture and cultured for 6 h. Then the culture medium was removed and replaced with DMEM-F12 medium containing 10% fetal bovine serum. The cells were cultured for 48–72 h.

### 2.12 Quantification of apoptosis by flow cytometry

Flow cytometry was be used to estimate the rate of H9c2 cells apoptosis after DOX treatment. The H9c2 cells were trypsinizedand and collected by centrifugation. The cells were washed twice with PBS and stained with Annexin V-FITC for 15 min followed by incubation with propidium iodide for 5 min at room temperature in the dark. Then, the cells were analyzed using a flow cytometer (BD Biosciences). The percentage of apoptotic cells were evaluated for different groups of cells.

### 2.13 Detection serum lactate dehydrogenase (LDH), creatine kinase (CK) and aspartate aminotransferase (AST)

Serum LDH, CK and AST levels were estimated using commercial kits (Nanjing Jiancheng Bioengineering Institute, China, Beyotime Institute of Biotechnology) according to manufacturer's instructions.

### 2.14 Western blot analysis

Cardiac tissue samples and H9c2 cells were lysed using the RIPA lysis buffer [50 mM Tris (pH 7.4), 150 mM NaCl, 1% Triton X-100, 1% sodium deoxycholate, 0.1% SDS] containing sodium orthovanadate, sodium fluoride, EDTA, and leupeptin, and 100 × protease and phosphatase inhibitors (Beyotime Institute of Biotechnology). Total protein concentrations were estimated using the BCA protein assay kit (Beyotime Institute of Biotechnology). Protein samples (40 *μ*g/pore) were mixed with loading buffer and SDS sample buffer, denatured at 95°C for 5 min, and separated on a SDS-PAGE. The separated proteins were transferred to the PVDF membrane. The membrane was blocked blots with 5% BSA (V900933, Sigma, Germany) at room temperature for 2 h. Then, the blots were incubated at 4°C overnight with primary antibodies against the following proteins: HDAC2 (1:1,000; ab219053; Abcam), p53 (1:1,000; Cat. No. 32532; Cell Signaling Technology.), cleaved caspase 3 (1:1,000; Cat. No. 9661S; Cell Signaling Technology.), cleaved caspase 9 (1:1,000; Cat. No. 10380-1-AP; Proteintech), p-AKT (1:2,000; Cat. No. 4060; Cell Signaling Technology.), AKT (1:2,000; Cat. No. 60203-2-lg; Proteintech), Bax (1:1,000; ab32503; Abcam), Bcl-2 (1:1,000; ab194583; Abcam) and β-actin (1:1,000; Cat. No. 4970S; Cell Signaling Technology.). The blots were washed and incubated with horseradish peroxidase-conjugated secondary antibodies for 1 h. Then, the blots were developed with the enhanced chemiluminescence (ECL) reagent and visualization by chemiluminescent imaging. The intensity of the protein bands were quantified using the Image J software.

### 2.15 Statistical analysis

Statistical data was analyzed using the GraphPad Prism 8.0 software (GraphPad Software Company, San Diego, CA, USA). Normally distributed continuous data was presented as mean ± standard deviation (SD). The differences in normally distributed continuous data between two groups analyzed using the *T*-test, whereas one-way ANOVA was used to analyze the differences between multiple groups. *P* < 0.05 was considered to as statistically significant.

## 3 Results

### 3.1 DOX-induced cardiac dysfunction is associated with increased HDAC2 expression *in vitro*

**Figure 1:**
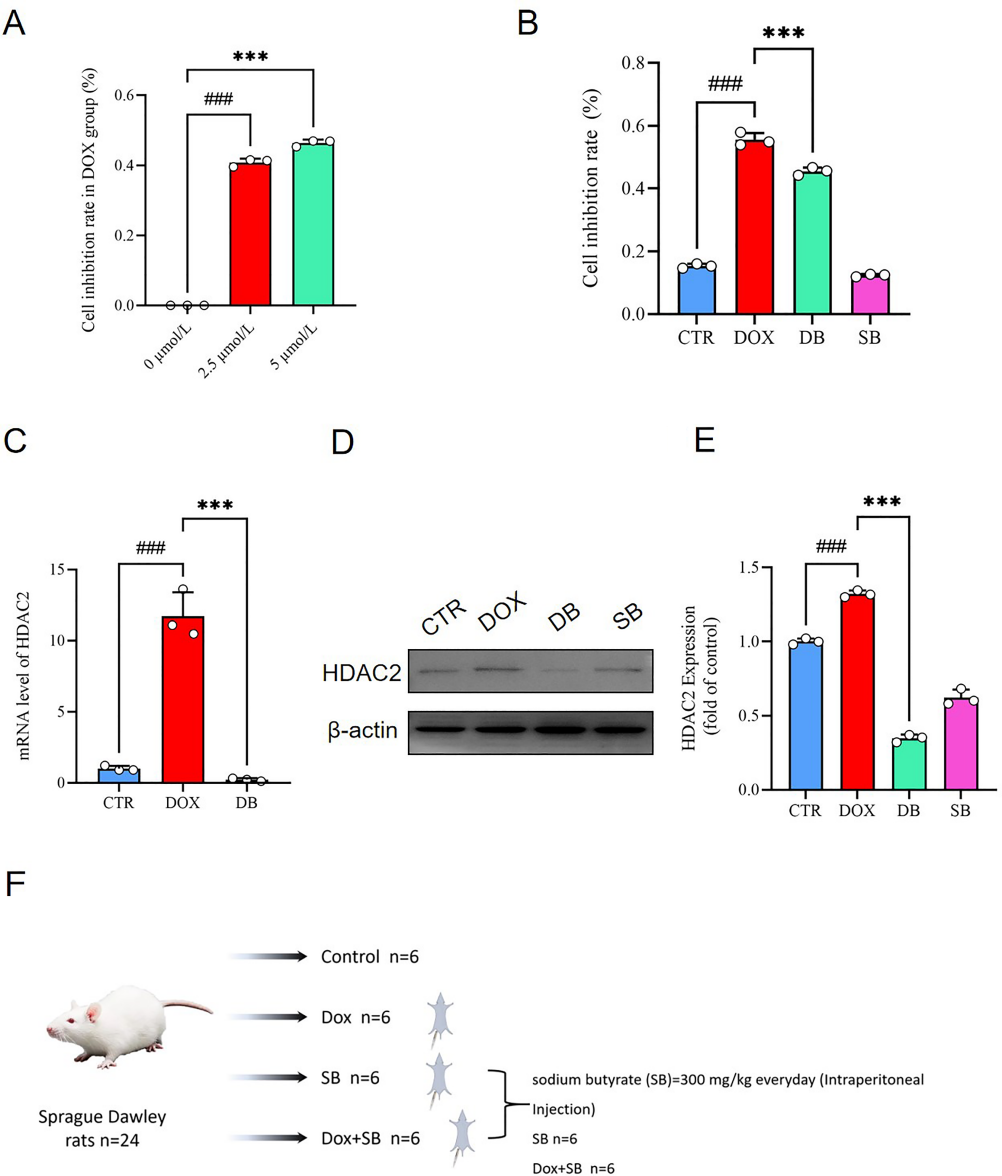
**(A)** CCK-8 assay results show the viability of H9c2 cells treated with different concentrations of DOX (0, 2.5, and 5.0 *µ*mol/L). ###*P* < 0.001 for 2.5 *μ*mol/L vs. 0 *μ*mol/L; ***P < 0.001 for 5.0 *μ*mol/L vs. 2.5 *μ*mol/L. **(B)** CCK-8 assay results show the viability of H9c2 cells in the control (CTR), DOX, DB, and SB groups. ###*P* < 0.001 for DOX vs. CTR; ***P < 0.001 for DB vs. DOX. **(C)** HDAC2 mRNA levels in the CTR, DOX, and DB groups of H9c2 cells. ###*P* < 0.001 for DOX vs. CTR; ***P < 0.001 for DB vs. DOX. **(D)** Representative image of the western blot shows HDAC2 and β-actin protein levels in the H9c2 cells belonging to the CTR, DOX, DB, and SB groups. **(E)** The relative expression levels of HDAC2 protein in the CTR, DOX, DB, and SB groups. Note:. ###*P* < 0.001 for DOX vs. CTR; ***P < 0.001 for DB vs. DOX. **(F)** The flowchart shows the establishment of animal model to evaluate DOX-induced cardiotoxicity and the effects of HDCA2 inhibition using SB. CTR, control; DOX, Doxorubicin; DB, Doxorubicin + SB; SB, control + sodium butyrate.

CCK 8 assay results demonstrated that DOX significantly reduced the viability of H9c2 cells in a dose-dependent manner (Figure 1A). However, SB significantly reduced the DOX-induced cytotoxicity of the H9c2 cells (Figure 1B). These findings suggested that DOX-induced apoptosis in the H9c2 cardiomyocytes. These results were consistent with previously reported findings (28, 29). Next, we analyzed the HDAC2 mRNA and protein levels. Our data showed that HDAC2 mRNA and protein levels significantly elevated in the DOX group, but reduced in the DOX + SB (DB) treatment group (Figure 1C–E).

### 3.2 Inhibition of HDAC2 alleviates DOX-induced cardiac injury in rats

**Figure 2:**
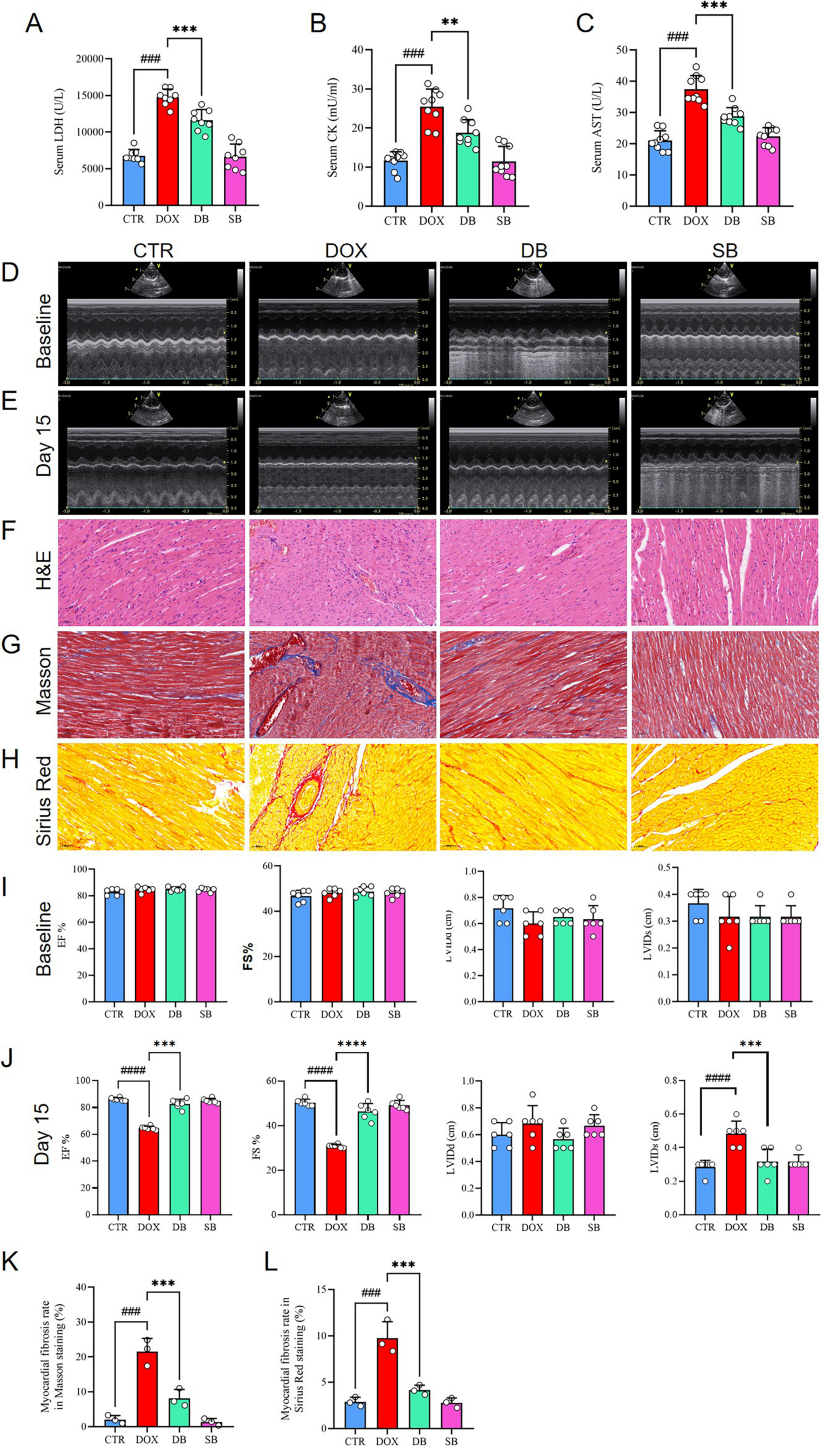
**(A)** Serum levels of LDH in the CTR, DOX, DB, and SB groups of rats. ###*P* < 0.001 for DOX vs. CTR; ***P < 0.001 for DB vs. DOX. **(B)** Serum levels of CK in the CTR, DOX, DB, and SB groups of rats. ###*P* < 0.001 for DOX vs. CTR; **P < 0.01 for DB vs. DOX. **(C)** Serum levels of AST in the CTR, DOX, DB, and SB groups of rats. ###*P* < 0.001 for DOX vs. CTR; ***P < 0.001 for DB vs. DOX. **(D)** M-mode echocardiography of rats in the CTR, DOX, DB, and SB groups at baseline. **(E)** M-mode echocardiography of rats in the CTR, DOX, DB, and SB groups at day 15. **(F-H)** Representative images of H&E staining, Masson, and Sirius Red stained cardiac tissue sections from the CTR, DOX, DB, and SB groups of rats. Scale bar = 50 µm. **(I-J)** Echocardiography data analysis of the CTR, DOX, DB, and SB groups of rats at **(I)** baseline and **(J)** day 15 of treatment *n* = 6 rats per group). ####*P* < 0.0001 for DOX vs. CTR; ****P < 0.0001 for DB vs. DOX; ***P < 0.001 for DB vs. DOX. **(K)** Bar graph shows the myocardial fibrosis rate based on Masson staining of the cardiac tissue sections from the CTR, DOX, DB, and SB groups of rats (*n* = 3 per group). ###*P* < 0.001 for DOX vs. CTR; ***P < 0.001 for DB vs. DOX. **(L)** Bar graph shows the myocardial fibrosis rate based on the Sirius Red staining of the cardiac tissues from the CTR, DOX, DB, and SB groups of rats (*n* = 3 per group). ###*P* < 0.001 for DOX vs. CTR; ***P < 0.001 for DB vs. DOX. CTR, control; DOX, Doxorubicin; DB, Doxorubicin + SB; SB, control + sodium butyrate.

To investigate the role of HDAC2 in the DOX-induced cardiac dysfunction, we established a model of DOX-induced cardiotoxicity in rats through intraperitoneal injections of DOX (Figure 1F). The serum levels of creatine kinase (CK), lactate dehydrogenase (LDH), and aspartate aminotransferase (AST) were significantly elevated in the DOX treatment group compared with the control group, but these effects were abrogated in the DB treatment group (Figure 2A–C). The data of Echo as shown in Table 1. At baseline, there were no statistically significant difference between the DOX group and control group in the left ventricular end-diastolic diameter (LVIDd): (0.72 cm ± 0.09 cm vs. 0.6 cm ± 0.08 cm *P* = 0.14), left ventricular end-systolic diameter (LVIDs): (0.37 cm ± 0.05 cm vs. 0.32 cm ± 0.07 cm *P* = 0.39), ejection fraction (EF%): (82.83% ± 2.11% vs. 84.67% ± 1.89% *P* = 0.37), and fractional shortening (FS%): (46.67% ± 2.36% vs. 48.17% ± 1.7% *P* = 0.62). However, compared to the control group, the DOX group rats exhibited characteristics of systolic dysfunction on day 15 of DOX treatment including significantly lower ejection fraction (EF%) (86.17% ± 1.07% vs. 64.17% ± 1.25%, *P* < 0.0001) and fractional shortening (FS%) (50.33% ± 1.37% vs. 30.83% ± 0.69%, *P* < 0.0001). The DOX group rats also showed significantly higher LVIDs compared to the control group (0.28 cm ± 0.04 cm vs. 0.48 cm ± 0.07 cm, *P* < 0.0001). However, there were no statistically significant differences in the LVIDd values between the two groups. Compared to the DOX group, the DB treatment group exhibited significant improvements in the EF% (64.17% ± 1.25% vs. 82.67% ± 3.14%, *P* < 0.001) and FS% (30.83% ± 0.69% vs. 46.33% ± 3.25%, *P* < 0.0001). As shown, SB treatment partially improved left ventricular systolic function but remained lower than those of the control group. (Figure 2D,E,1,J). We then performed H&E staining analysis to determine the morphological changes in the cardiac tissues. Compared to the control group, the cardiac tissue sections from the DOX group rats exhibited disordered arrangement of the cardiomyocytes, but the cardiac tissue sections from the DB treatment group showed more orderly arrangement of the cardiomyocytes (Figure 2F). Next, we evaluated cardiac fibrosis, a hallmark of adverse cardiac remodeling after myocardial injury, by performing Masson's and Sirius Red staining of the cardiac tissue sections. The DOX treatment group demonstratedhighly disordered myocardium and significantly increased collagen fiber area compared to the control group, but htese pathological changes were significant reduced in the DB group (Figure 2G,H,K,L). Collectively, these findings demonstrated that DOX treatment induce cardiac dysfunction, but HDAC2 inhibition by SB significantly attenuated DOX-induced cardiac injury *in vivo*.

### 3.3 HDAC2 expression is closely associated with DOX-induced myocardiocyte apoptosis in rats

**Figure 3:**
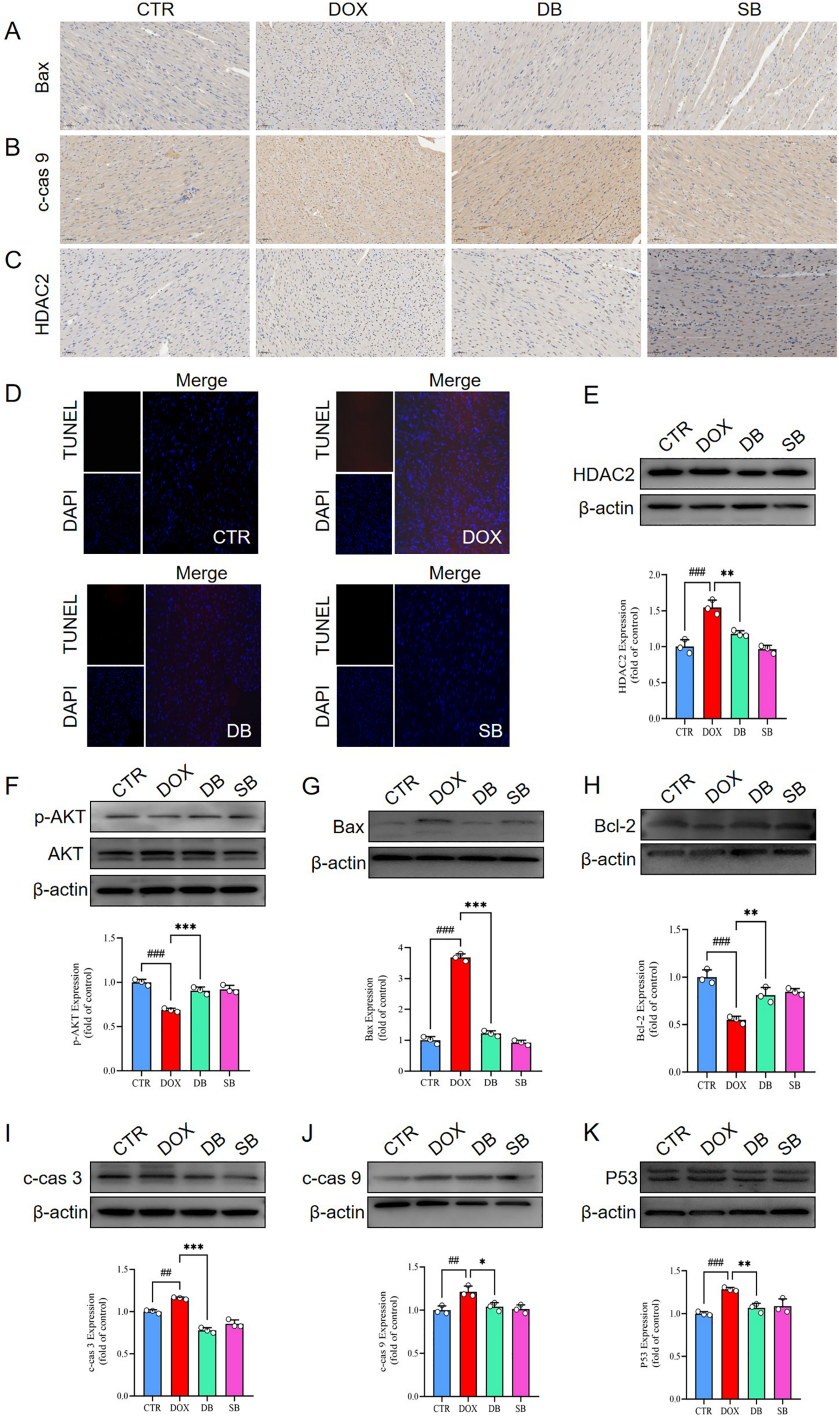
**(A-C)** Representative IHC staining images show the expression levels of Bax, c-cas 9, and HDAC2 proteins in the cardiac tissues of the CTR, DOX, DB, and SB groups of rats. Scale bar = 50 *µ*m. **(D)** Representative images of TUNEL staining show the apoptosis rate in the cardiac tissues of the CTR, DOX, DB, and SB groups of rats. **(E)** Western blotting results show the HDAC2 protein levels in the cardiac tissues of the CTR, DOX, DB, and SB groups of rats. ###*P* < 0.001 for DOX vs. CTR; **P < 0.01 for DB vs. DOX. **(F)** Western blotting results show the AKT and p-AKT levels in the cardiac tissues of the CTR, DOX, DB, and SB groups of rats. ###*P* < 0.001 for DOX vs. CTR; ***P < 0.001 for DB vs. DOX. **(G)** Western blotting results show the Bax protein levels in the cardiac tissues of the CTR, DOX, DB, and SB groups of rats. ###*P* < 0.001 for DOX vs. CTR; ***P < 0.001 for DB vs. DOX. **(H)** Western blotting results show the Bcl-2 protein levels in the cardiac tissues of the CTR, DOX, DB, and SB groups of rats. ###*P* < 0.001 for DOX vs. CTR; **P < 0.01 for DB vs. DOX. **(I)** Western blotting results show the c-cas 3 protein levels in the cardiac tissues of the CTR, DOX, DB, and SB groups of rats. ##*P* < 0.01 for DOX vs. CTR; ***P < 0.001 for DB vs. DOX. **(J)** Western blotting results show the c-cas 9 protein levels in the cardiac tissues of the CTR, DOX, DB, and SB groups of rats. ##*P* < 0.01 for DOX vs. CTR; *P < 0.05 for DB vs. DOX. **(K)** Western blotting results show the p53 protein levels in the cardiac tissues of the CTR, DOX, DB, and SB groups of rats. ###*P* < 0.001 for DOX vs. CTR; **P < 0.01 for DB vs. DOX. CTR, control; DOX, Doxorubicin; DB, Doxorubicin + SB; SB, control + sodium butyrate; c-cas 3, cleaved caspase 3; c-cas 9, cleaved caspase 9.

To further investigate the relationship between HDAC2 expression and cardiomyocyte apoptosis, we performed immunohistochemistry (IHC) and TUNEL staining analyses. In the IHC analysis, DOX group demonstrated higher number of positively stained nuclei for HDAC2, cleaved caspase-9, and Bax compared to the control group, but these pathological changes were significantly reduced in the DB treatment group (Figure 3A–C). The number of TUNEL-positive cells were significantly higher in the DOX group compared to the control group, but significantly reduced in the DB group relative to the DOX group (Figure 3D). Subsequently, we performed western blotting analysis to determine whether DOX treatment induced cardiomyocyte apoptosis. Western blotting results showed that DOX treatment increased the expression levels of HDAC2, Bax, cleaved caspase-3 (c-cas 3), cleaved caspase-9 (c-cas 9), and p53, and decreased Bcl-2 levels (Figure 3E,G–K). Furthermore, DOX treatment reduced the levels of AKT phosphorylation was reduced in the DOX-treated group (Figure 3F). However, these pathological changes were significantly alleviated by inhibiting HDAC2 with SB (Figure 3E–K).

These findings demonstrated that DOX treatment induced cardiomyocyte apoptosis in the rat hearts by increasing HDAC2 expression. However, DOX-induced cardiomyocyte apoptosis was significantly reduced by inhibiting HDAC2 expression with SB. Therefore, these results highlighted that HDAC2 was a potential therapeutic target for alleviating DOX-induced cardiomyocyte apoptosis.

### 3.4 HDAC2 suppression alleviates DOX-induced apoptosis in the H9c2 cells by activating the PI3K/AKT signaling pathway

**Figure 4:**
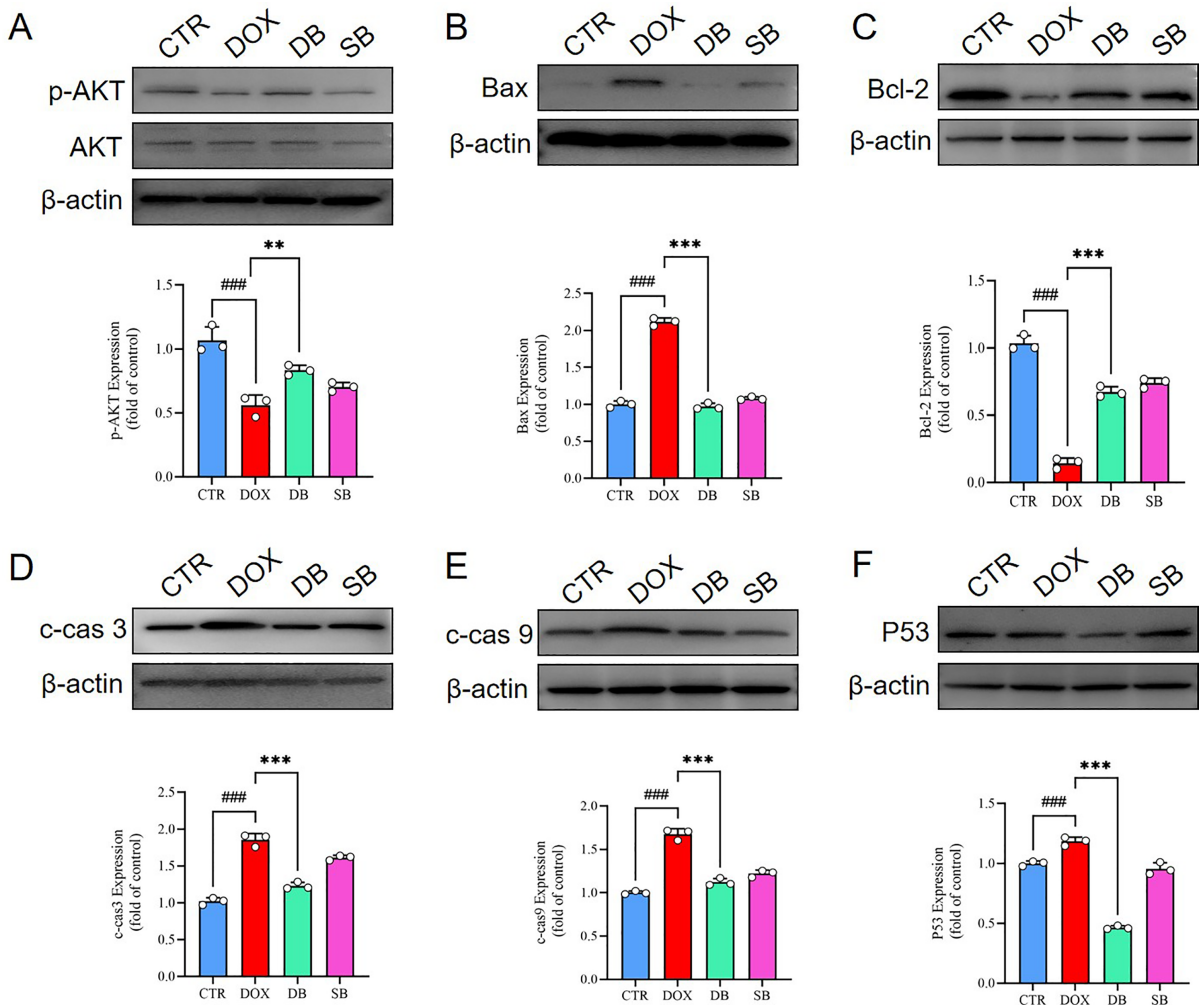
**(A)** Western blotting results show the AKT and p-AKT levels in the H9c2 cells belonging to the CTR, DOX, DB, and SB groups. ###*P* < 0.001 for DOX vs. CTR; **P < 0.01 for DB vs. DOX. **(B)** Western blotting results show the Bax protein levels in the H9c2 cells belonging to the CTR, DOX, DB, and SB groups. ###*P* < 0.001 for DOX vs. CTR; ***P < 0.001 for DB vs. DOX. **(C)** Western blotting results show the Bcl-2 protein levels in the H9c2 cells belonging to the CTR, DOX, DB, and SB groups. ###*P* < 0.001for DOX vs. CTR; ***P < 0.001 for DB vs. DOX. **(D)** Western blotting results show the c-cas 3 protein levels in the H9c2 cells belonging to the CTR, DOX, DB, and SB groups. ###*P* < 0.001 for DOX vs. CTR; ***P < 0.001 for DB vs. DOX. **(E)** Western blotting results show the c-cas 9 protein levels in the H9c2 cells belonging to the CTR, DOX, DB, and SB groups. ###*P* < 0.001 for DOX vs. CTR; ***P < 0.001 for DB vs. DOX. **(F)** Western blotting results show the p53 protein levels in the H9c2 cells belonging to the CTR, DOX, DB, and SB groups. ###*P* < 0.001 for DOX vs. CTR; ***P < 0.001 for DB vs. DOX. CTR, control; DOX, Doxorubicin; DB, Doxorubicin + SB; SB, control + sodium butyrate; c-cas 3, cleaved caspase 3; c-cas 9, cleaved caspase 9.

The PI3K/AKT signaling pathway is widely recognized as a critical regulator of celluar apoptosis. The levels of phosphorylated AKT was related with the extent of apoptosis. Western blotting analysis demonstrated that phosphorylated AKT levels were significantly upregulated in the DB group compared to the DOX group *in vitro* (Figure 4A). Furthermore, DB group showed significantly reduced expression of pro-apoptotic markers such as Bax, cleaved caspase-3 (c-cas 3), cleaved caspase-9 (c-cas 9), and p53, and increased the expression of the anti-apoptotic marker Bcl-2 compared to the DOX group (Figure 4B–F). These findings demonstrated that HDAC2 inhibitor SB mitigated DOX-induced apoptosis in the H9c2 cardiomyocytes by upregulating AKT phosphorylation.

### 3.5 Knocked down HDAC2 protects against DOX-induced cardiomyocyte apoptosis *via* PI3K/AKT signaling axis

**Figure 5:**
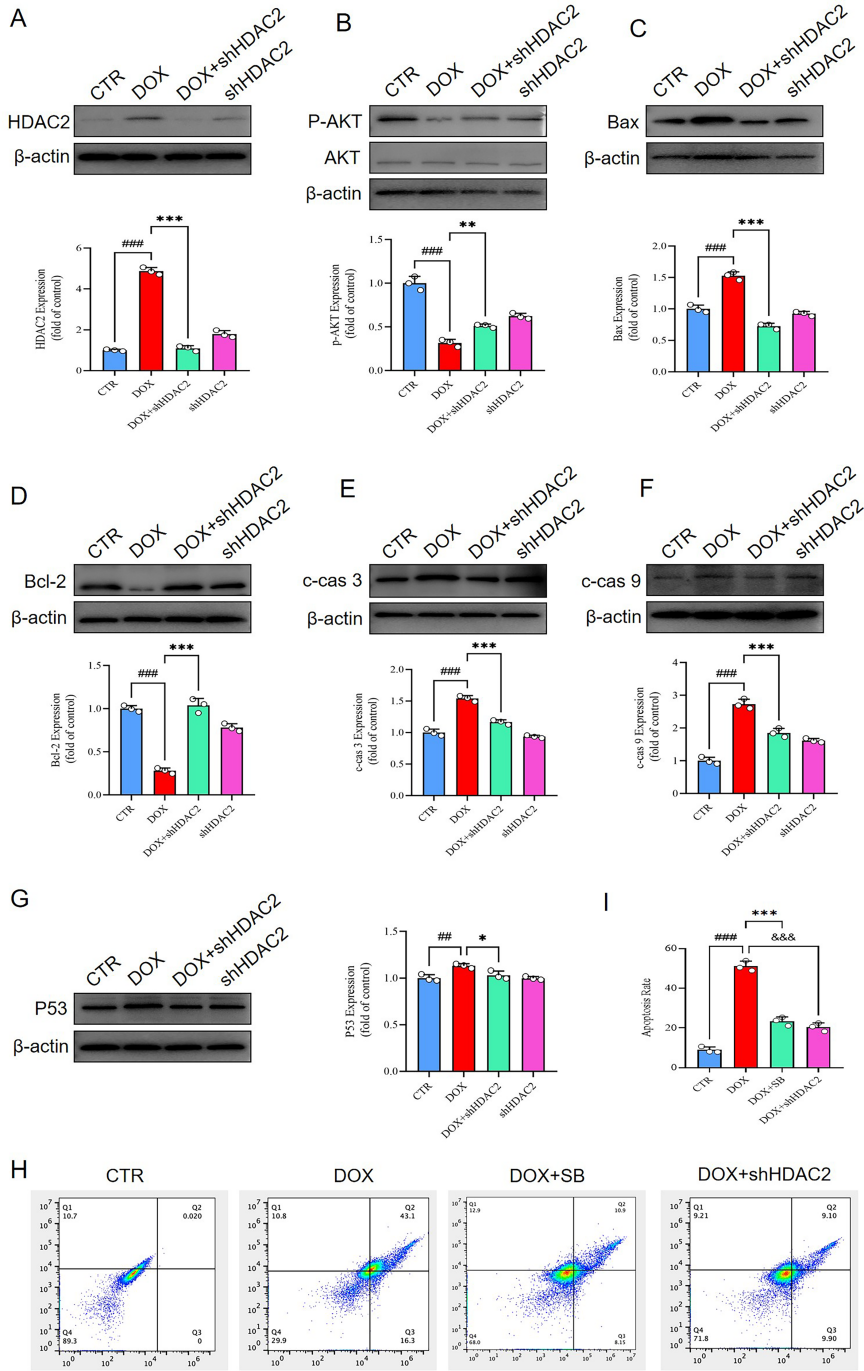
**(A)** Western blotting results show the HDAC2 protein levels in the H9c2 cells belonging to the CTR, DOX, DOX + shHDAC2, and shHDAC2 groups. ###*P* < 0.001 for DOX vs. CTR; ***P < 0.001 for DOX + shHDAC2 vs. DOX. **(B)** Western blotting results show the AKT and p-AKT levels in the H9c2 cells belonging to the CTR, DOX, DOX + shHDAC2, and shHDAC2 groups. ###*P* < 0.001 for DOX vs. CTR; **P < 0.01 for DOX + shHDAC2 vs. DOX. **(C)** Western blotting results show the Bax protein levels in the H9c2 cells belonging to the CTR, DOX, DOX + shHDAC2, and shHDAC2 groups. ###*P* < 0.001 for DOX vs. CTR; ***P < 0.001 for DOX + shHDAC2 vs. DOX. **(D)** Western blotting results show the Bcl-2 protein levels in the H9c2 cells belonging to the CTR, DOX, DOX + shHDAC2, and shHDAC2 groups. ###*P* < 0.001 for DOX vs. CTR; ***P < 0.001 for DOX + shHDAC2 vs. DOX. **(E)** Western blotting results show the c-cas 3 protein levels in the H9c2 cells belonging to the CTR, DOX, DOX + shHDAC2, and shHDAC2 groups. ###*P* < 0.001 for DOX vs. CTR; ***P < 0.001 for DOX + shHDAC2 vs. DOX. **(F)** Western blotting results show the c-cas 9 protein levels in the H9c2 cells belonging to the CTR, DOX, DOX + shHDAC2, and shHDAC2 groups. ###*P* < 0.001 for DOX vs. CTR; ***P < 0.001 for DOX + shHDAC2 vs. DOX. **(G)** Western blotting results show the p53 protein levels in the H9c2 cells. ###*P* < 0.001 for DOX vs. CTR; ***P < 0.001 for DOX + shHDAC2 vs. DOX. **(H-I)** Flow cytometry. ### *P* < 0.001 for DOX vs. CTR; ***P < 0.001 for DOX vs. DOX + SB; &&&*P* < 0.001 for DOX vs. DOX + shHDAC2. CTR, control; DOX, Doxorubicin; shHDAC2, knockdown HDAC2; SB, Sodium butyrate; c-cas 3, cleaved caspase 3; c-cas 9, cleaved caspase 9.

To determine whether HDAC2 mediated DOX-induced cardiomyocyte apoptosis via the PI3K/AKT pathway, we knocked down HDAC2 levels using shRNAs and assessed the activity of the PI3K/AKT pathway. The levels of phosphorylated AKT phosphorylation were significantly higher in the DOX + shHDAC2 group compared to the control group (Figure 5A,B). Furthermore, DOX + shHDAC2group cardiomyocytes showed reduced level of apoptosis related proteins, including Bax, cleaved caspase-3 (c-cas 3), cleaved caspase-9 (c-cas 9), and p53, and increased expression of anti-apoptotic protein Bcl-2 compared to the control group (Figure 5C–G). These findings were consistent with the *in vivo* data. Flow cytometry analysis further confirmed that HDAC2 inhibition alleviates apoptosis in the H9c2 cells (Figure 5H).

**Figure 6:**
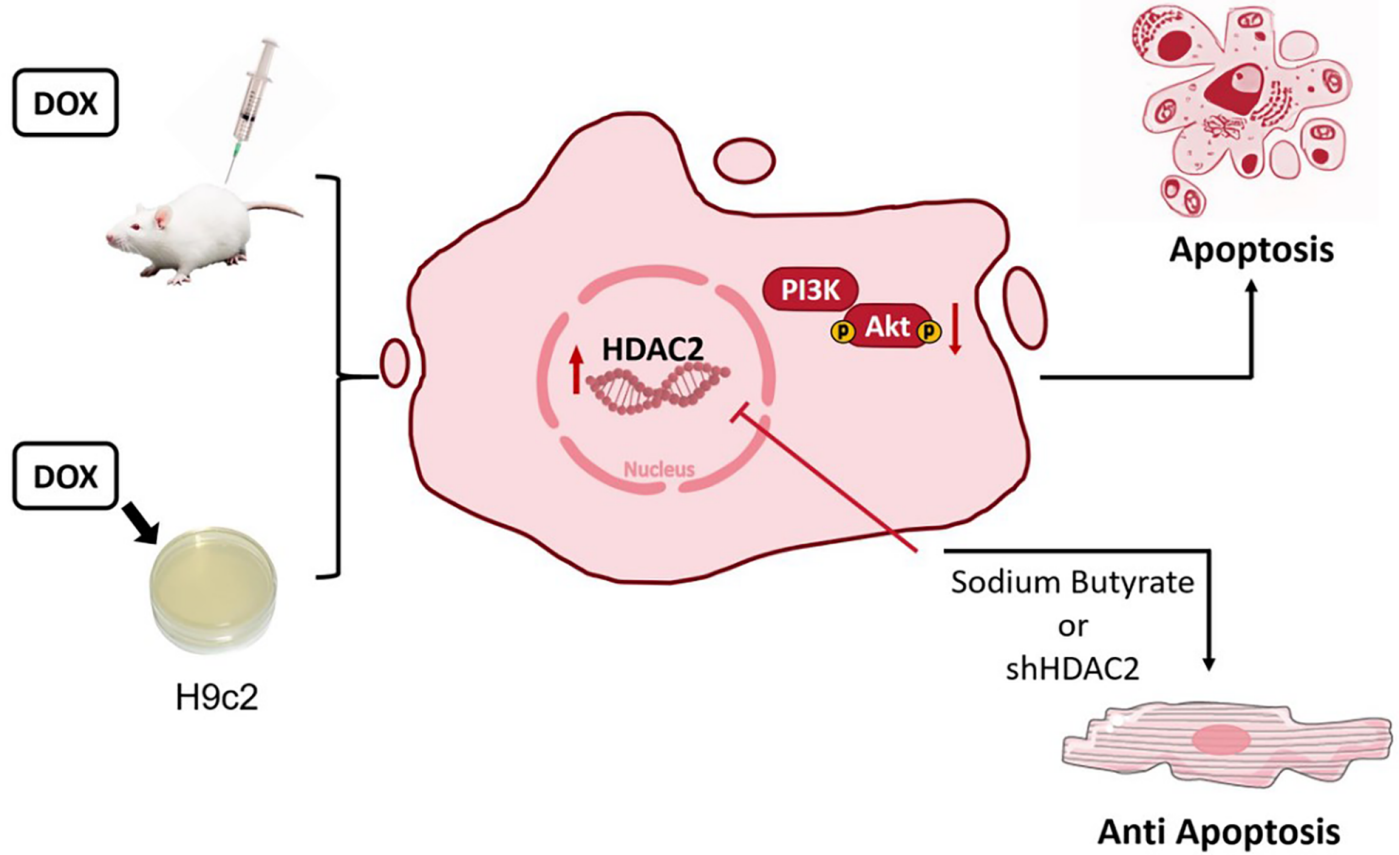
Mechanism chart.

Overall, these results demonstrated that HDAC2 mediated DOX-induced cardiomyocyte apoptosis by modulating PI3K/AKT activation (Figure 6).

## 4 Discussion

Advancements in cancer treatment, including chemotherapy, have significantly improved the survival rates of cancer patients. However, chemotherapy treatments are also associated with significant adverse effects. Doxorubicin (DOX) is a widely used chemotherapeutic drug against various hematological tumors and solid malignancies (3). However, its clinical application is limited by the enhanced risk of myocardial injury (30, 31). Cardiomyocyte apoptosis plays a central role in DOX-induced myocardial injury (32, 33). Previously studies demonstrated a link between HDAC2 expression and hypoxia-induced cardiomyocyte apoptosis. However, the relationship between HDAC2 expression and DOX-induced myocardial injury is poorly understood. Therefore, this study investigated the relationship between DOX-induced cardiomyocyte apoptosis and HDAC2 expression using both *in vitro* and *in vivo* models.

Our results showed that DOX treatment increased myocardial HDAC2 levels and induced myocardial injury and dysfunction. Western blot analysis confirmed that HDAC2 expression was significantly increased in the cardiac tissues of DOX-treated rats. Echocardiographic measurements demonstrated a marked decline in cardiac function in the DOX-treated rats compared to the control group, including reduced EF% (86.17% ± 1.07% vs. 64.17% ± 1.25%, *P* < 0.0001) and FS% (50.33% ± 1.37% vs. 30.83% ± 0.69%, *P* < 0.0001). Furthermore, there was a trend toward increased LVIDd in the DOX-treated rats (0.68 cm ± 0.12 cm vs. 0.60 cm ± 0.08 cm), but the difference was not statistically significant, probably because of the limited experimental duration. However, LVIDs was significantly increased in the DOX-treated group compared to the control group (0.28 cm ± 0.04 cm vs. 0.48 cm ± 0.07 cm, *P* < 0.0001). These pathological manifestations were further validated by histopathological analysis, including H&E staining, TUNEL staining, Masson's staining, and Sirius Red staining. These data suggested that DOX-induced cardiomyocyte apoptosis triggered adverse ventricular remodeling and was consistent with previously published research findings (34–36). Sodium butyrate (SB), a short-chain fatty acid (SCFA) (37), is a well-known inhibitor of HDAC2 expression. In the rat model of DOX-induced myocardial injury, treatment with DOX + SB improved LVIDd, LVIDs, cardiomyocyte apoptosis, FS% and EF%. These findings suggested a significant association between HDAC2 expression and DOX-induced cardiomyocyte apoptosis. Furthermore, both DOX + SB treatment and DOX + shHDAC2 (HDAC2 knockdown) demonstrated protective effects against DOX-induced apoptosis in the H9c2 cells, thereby supporting the potential link between HDAC2 and myocardial injury.

Epigenetic modifications play a significant role in cardiac pathological remodeling and the progression of heart diseases (38). HDAC2 is a member of class I HDACs (39) and has been implicated in various pathological processes; but, its precise role in DOX-induced cardiomyocyte apoptosis has been unclear. Our findings confirmed that DOX-induced cardiomyocyte apoptosis was associated with HDAC2 expression. PI3K/AKT signaling pathway plays a critical role in the regulation of cell proliferation and apoptosis (40). Activation of the PI3K/AKT pathway through phosphorylation of AKT inhibits apoptosis, whereas decreased phosphorylation of AKT exacerbates cellular apoptosis. Our study demonstrated that DOX treatment increased HDAC2 expression and reduced phospho-AKT levels in the cardiac tissues compared to the control group. Conversely, in the DOX + SB treatment group, HDAC2 expression was reduced, and phospho-AKT levels were significantly higher compared with the DOX group. These data were also consistent with the *in vitro* findings.

To further validate the relationship between HDAC2 and the PI3K/AKT pathway, we used SB, which is not specific HDAC2 inhibitor. To confirm whether the effects were mediated specifically through HDAC2 inhibition, we knocked down HDAC2 by transfecting H9c2 cells (shHDAC2). As expected, compared to the control group, phospho-AKT levels were higher in the DOX + shHDAC2 group, and the expression levels of apoptosis-related proteins and HDAC2 expression were significantly lower.

## 5 Conclusions

These findings suggested that HDAC2 was closely linked to the DOX-induced cardiomyocyte apoptosis by modulating the PI3K/AKT axis. Therefore, it is a promising therapeutic target for preventing DOX-induced myocardial injury. However, further in-depth studies are needed to elucidate the precise mechanisms by which HDAC2 regulates DOX-induced cardiomyocyte apoptosis via the PI3K/AKT signaling axis.

This study showed that suppression of HDAC2 protected against DOX-induced cardiomyocyte apoptosis by activating the PI3K/AKT axis. Therefore, HDAC2 is a potential therapeutic target for treating DOX-induced myocardial injury.

## Data Availability

The original contributions presented in the study are included in the article/Supplementary Material, further inquiries can be directed to the corresponding authors.
