# Supplementary material for: The role of HDAC2 inhibition in cardioprotection against doxorubicin-induced myocardial injury
Source: Front Cardiovasc Med. 2025 Feb 26;12:1557119. doi: 10.3389/fcvm.2025.1557119 (PMC11897267; doi:10.3389/fcvm.2025.1557119)

Western blotting in animal

From left to right is CTR DOX DB SB
CTR : control
DOX : Doxorubicin
DB : Doxorubicin + Sodium butyrate
SB : Sodium butyrate

HDAC2 - 1

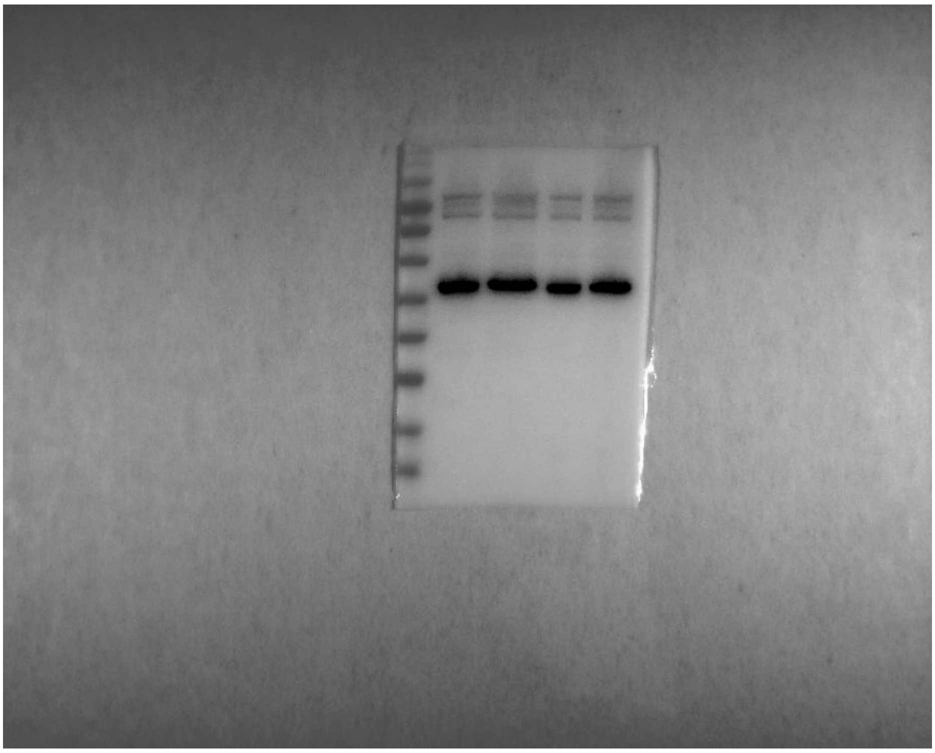

β - actin

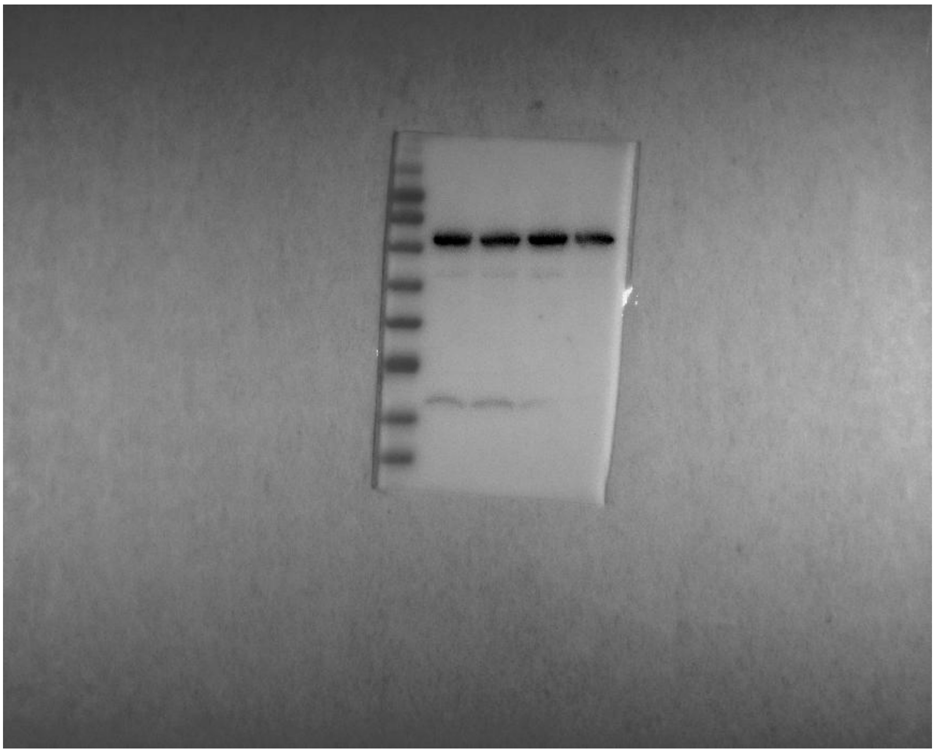

HDAC2 -2

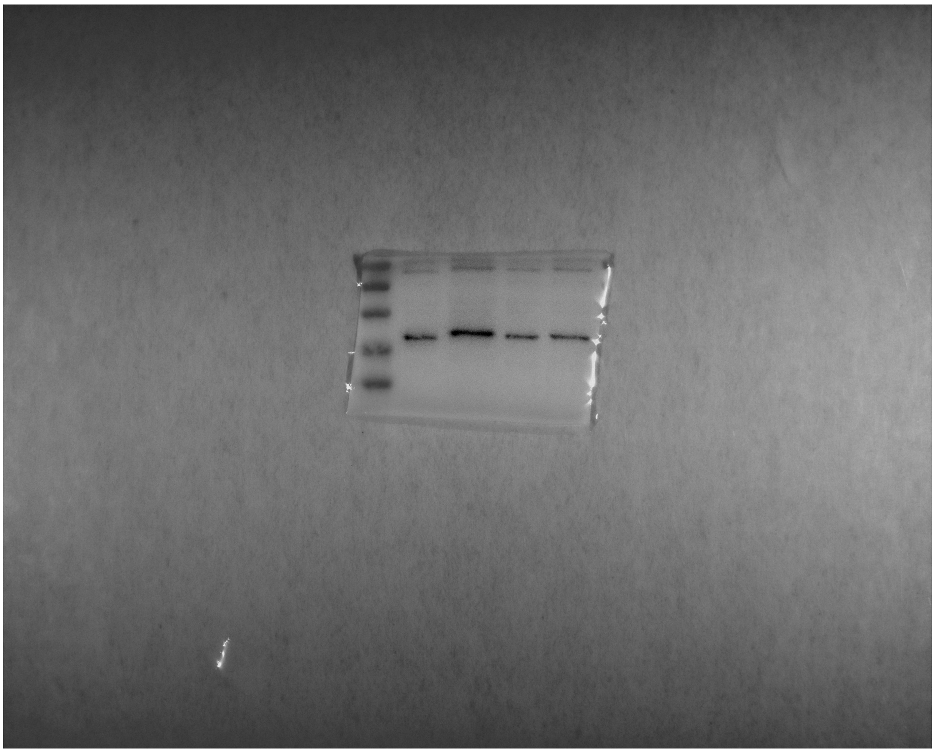

β - actin

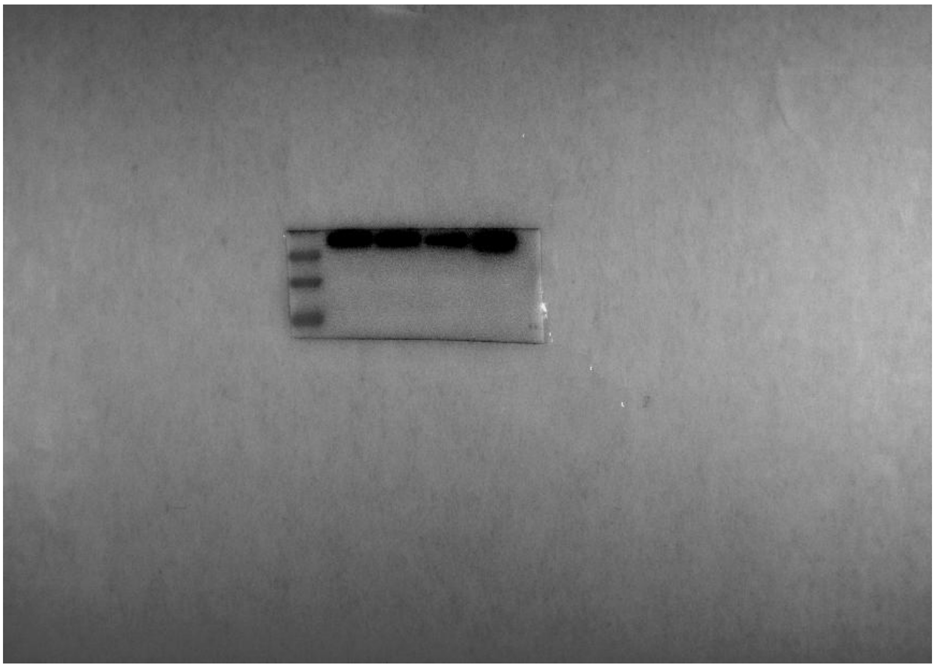

HDAC2 - 3

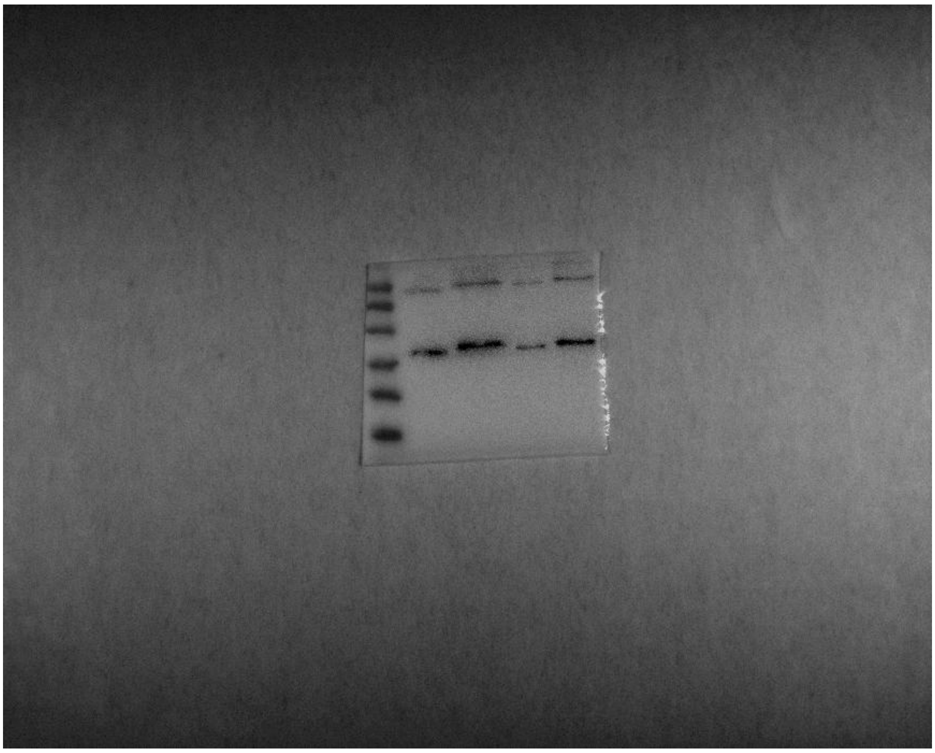

β - actin

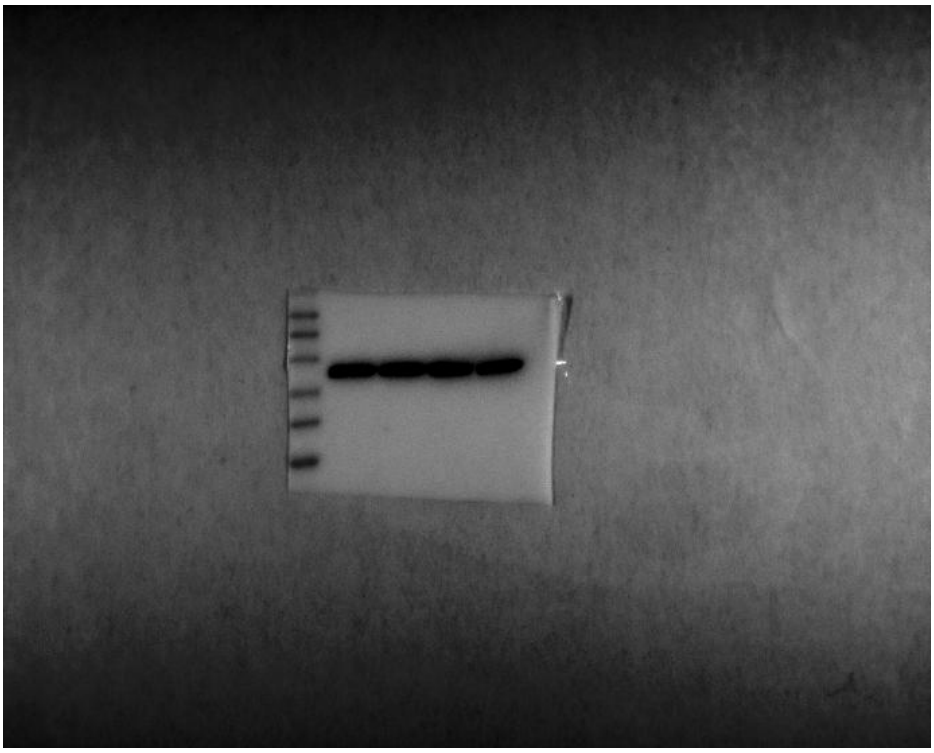

BAX - 1

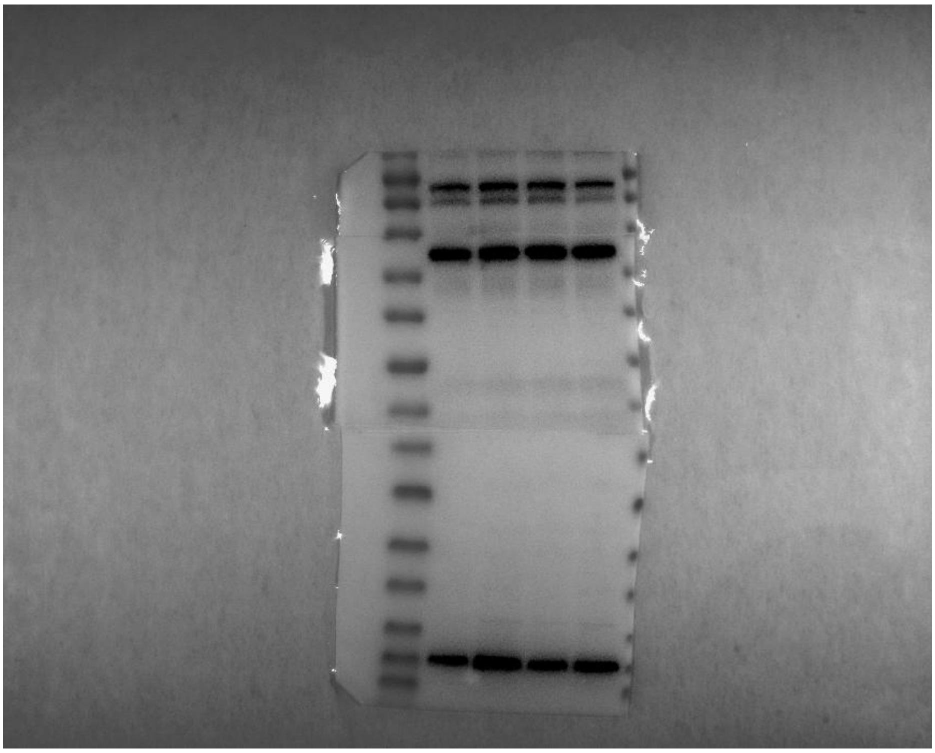

The below blot

β - actin

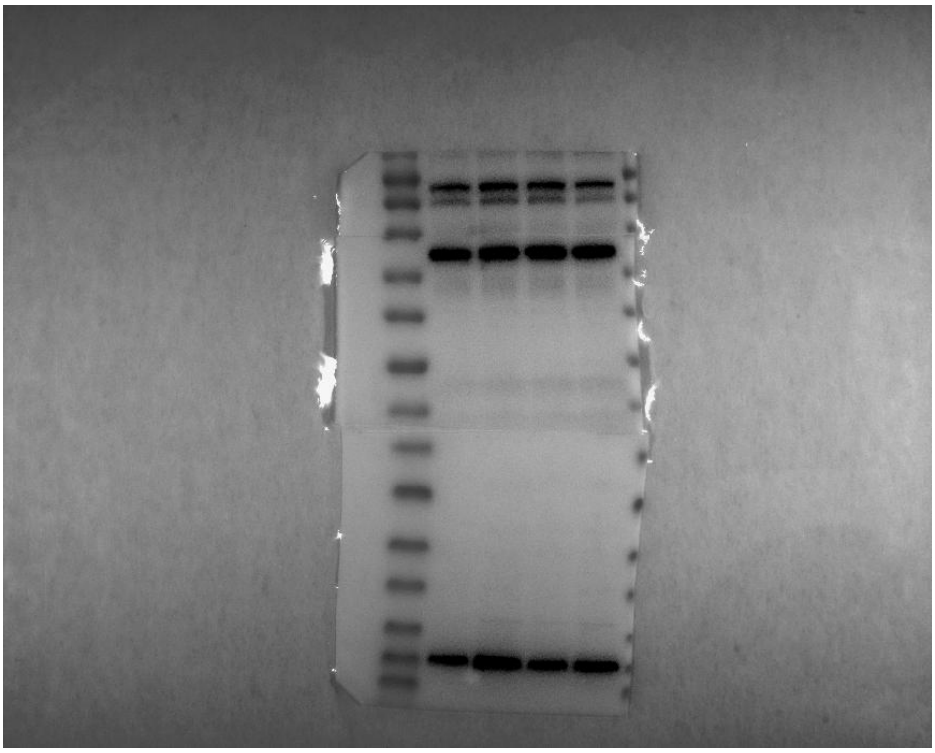

The middle blot

BAX - 2

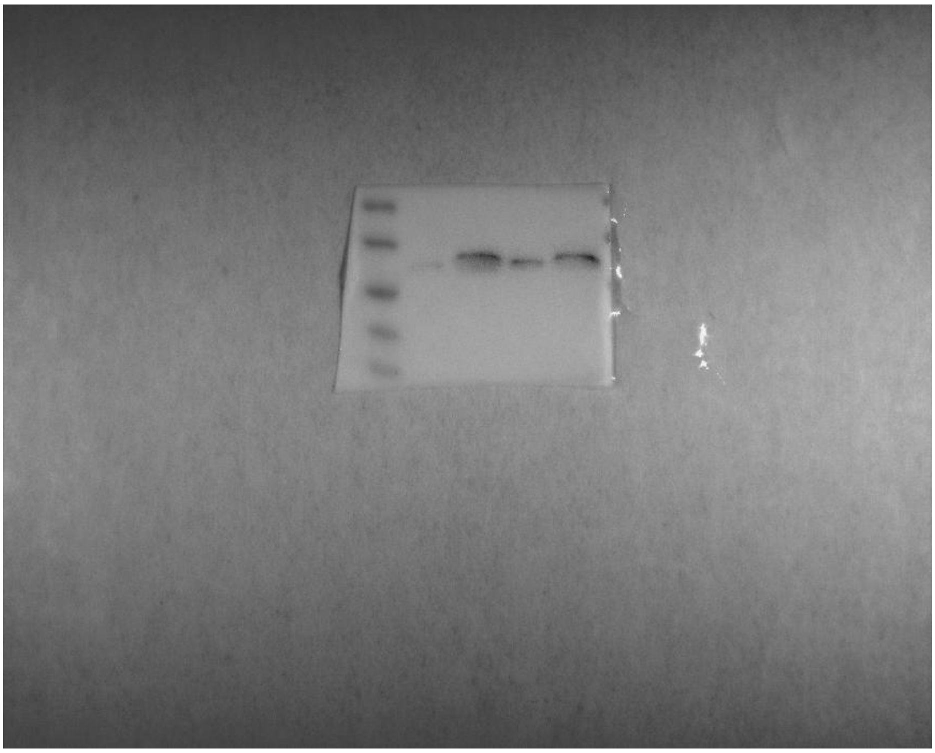

β - actin

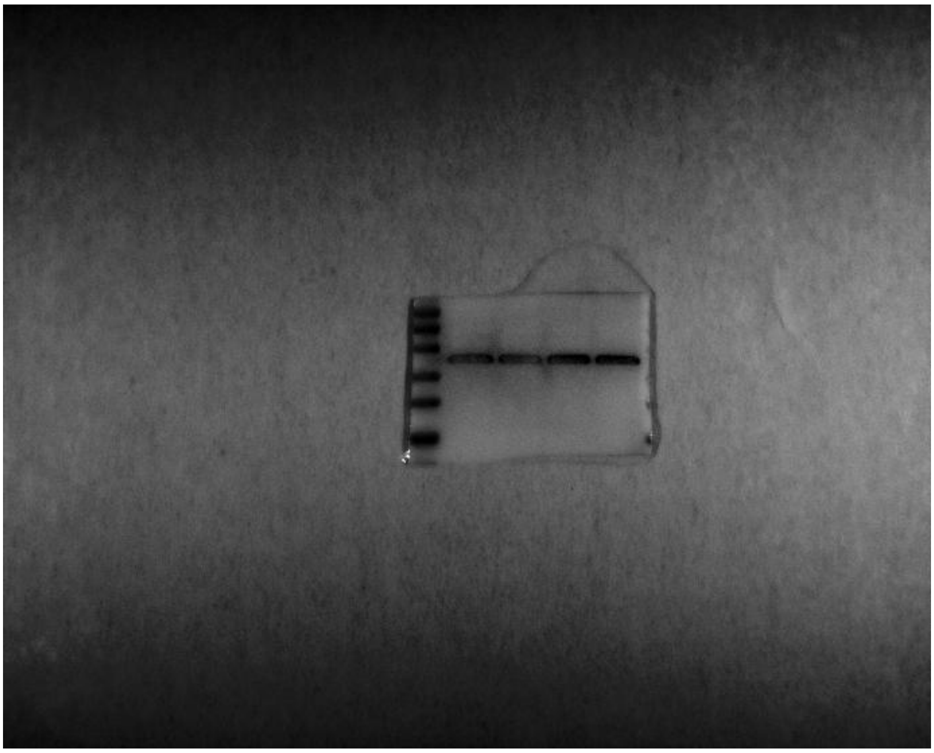

BAX - 3

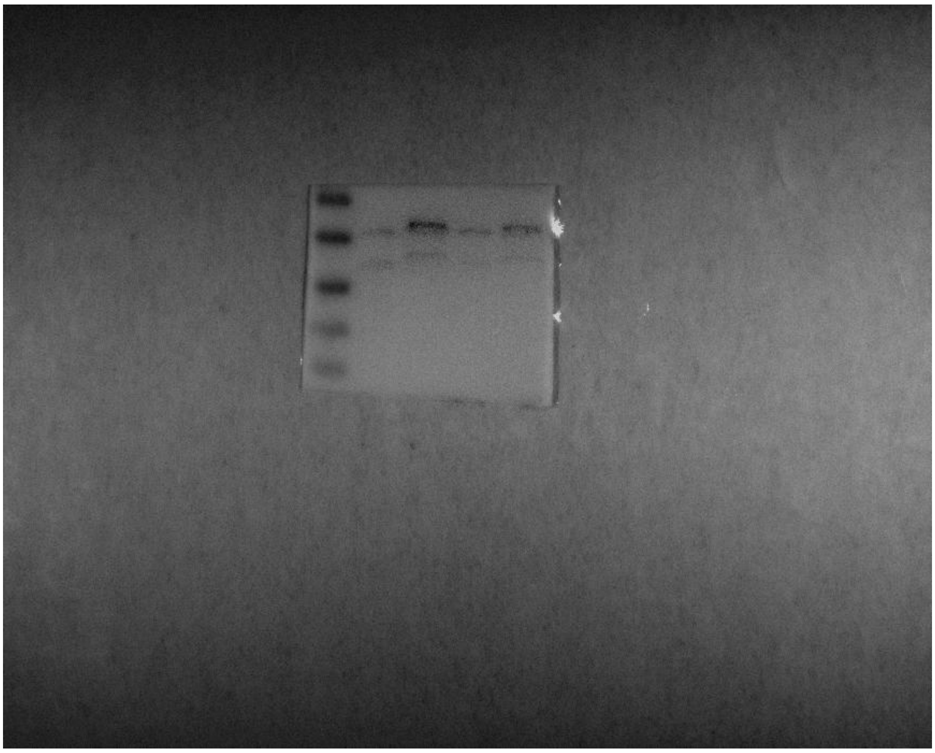

β - actin

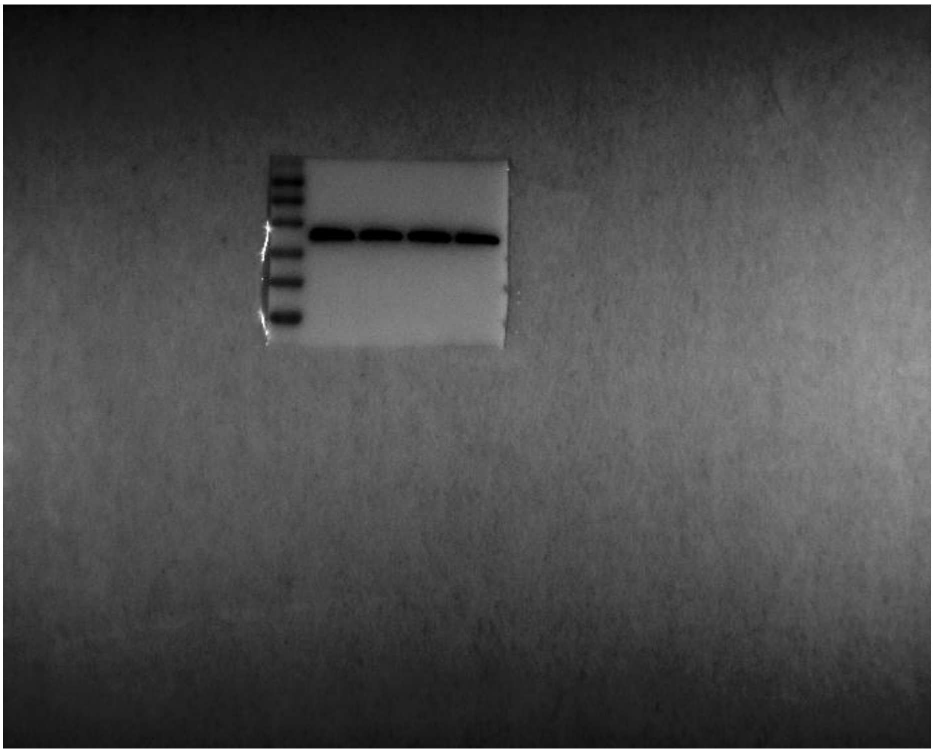

Bcl-2 - 1

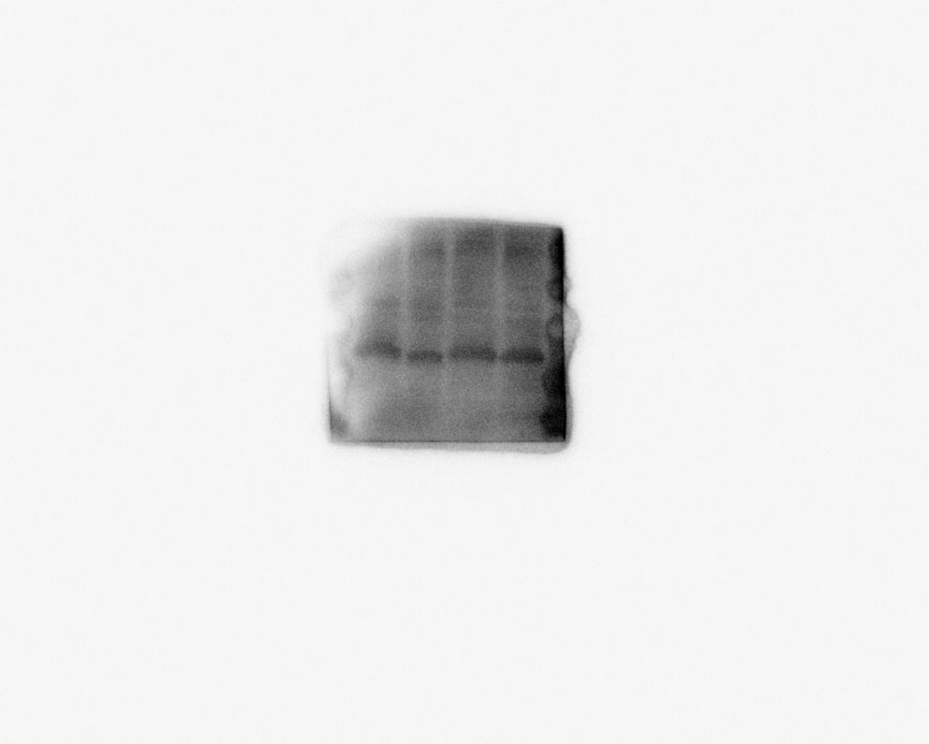

β - actin

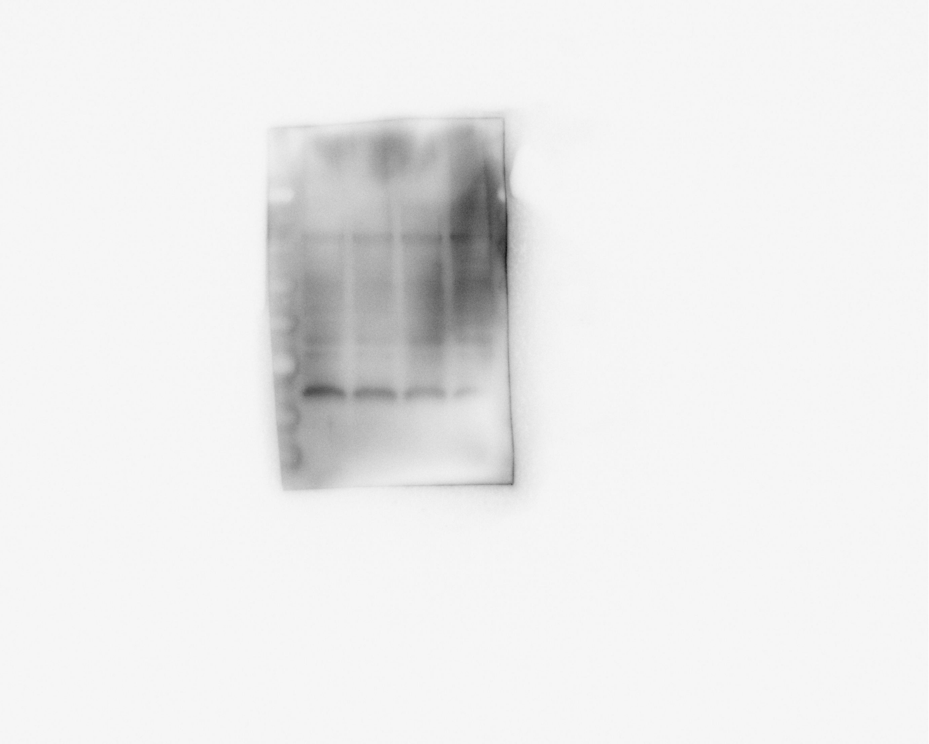

Bcl-2 - 2

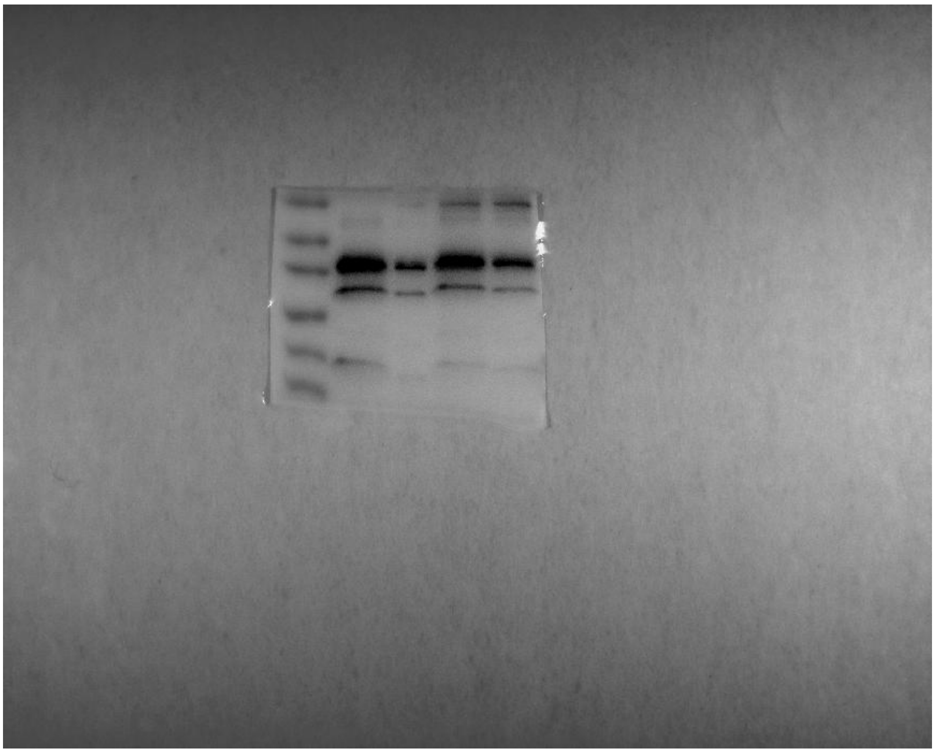

β - actin

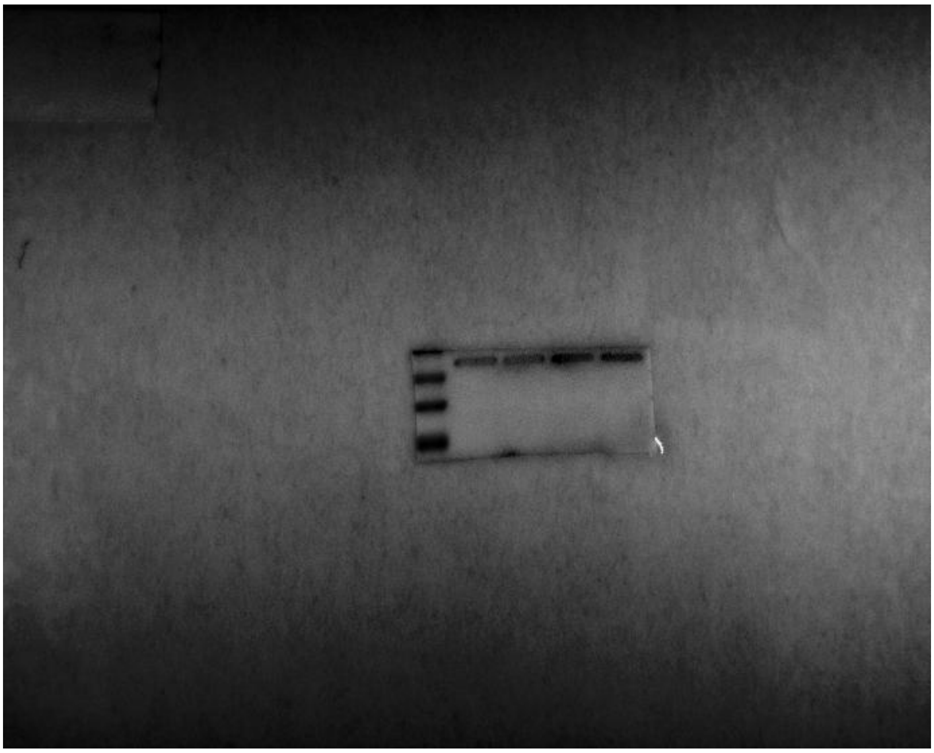

Bcl-2 - 3

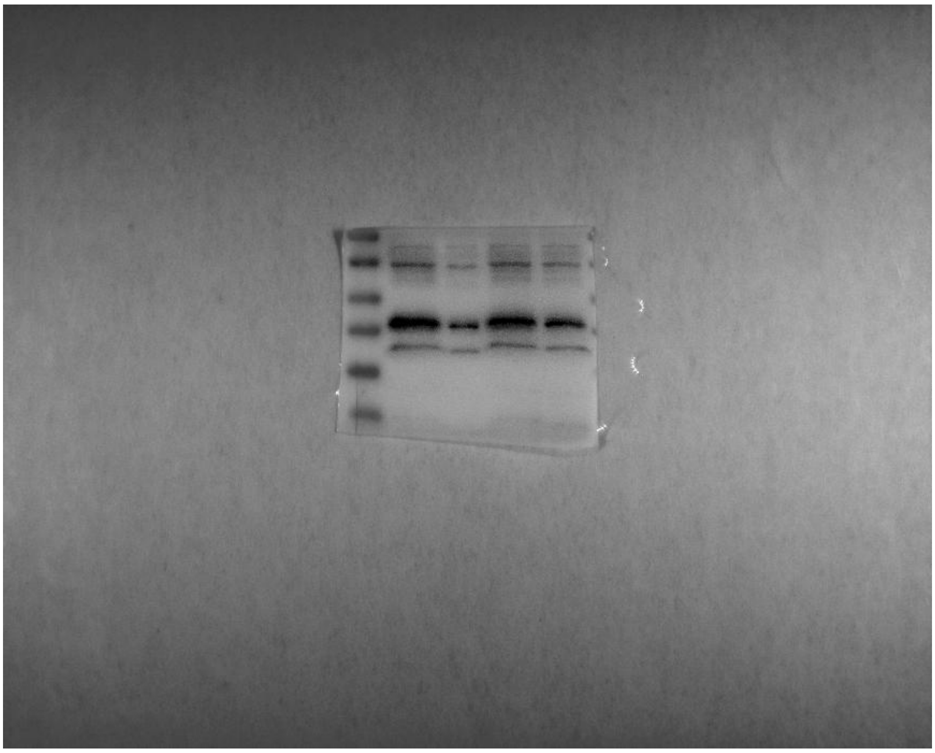

β - actin

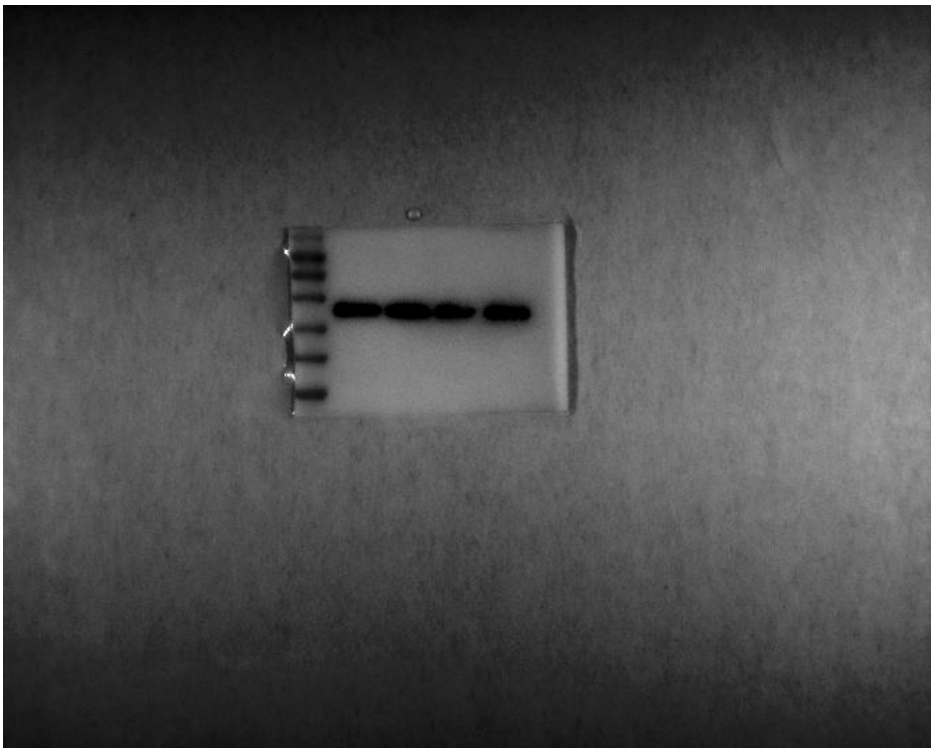

C-cas 3 - 1

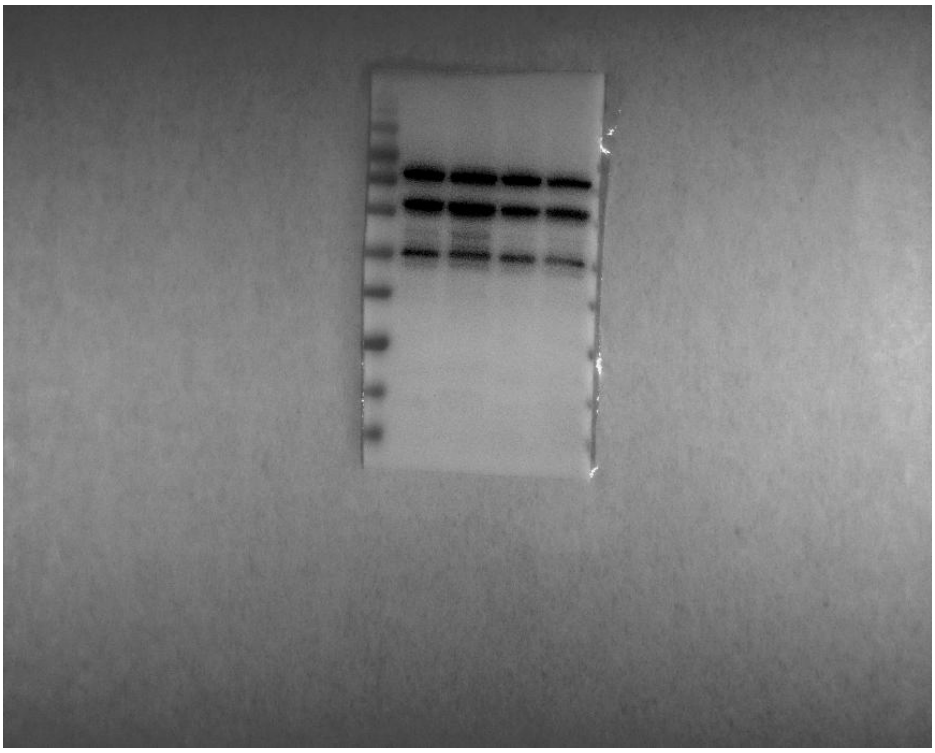

β - actin

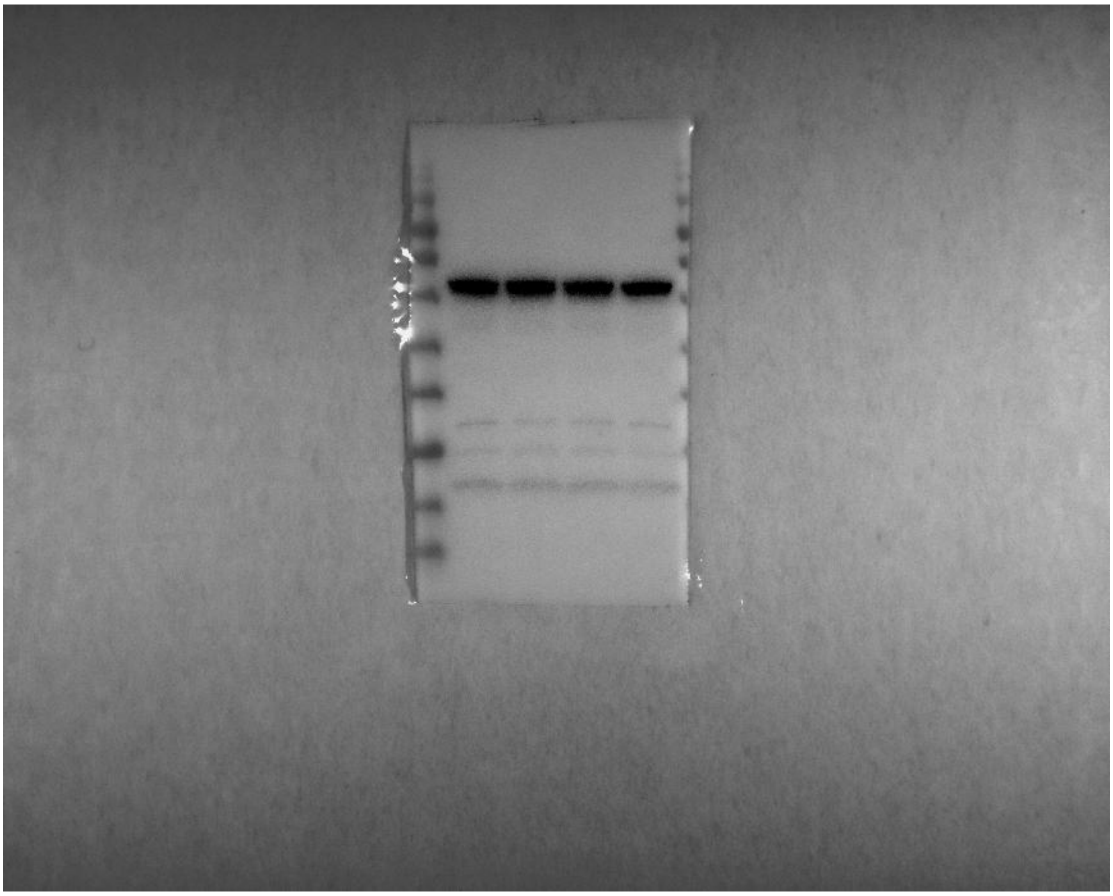

C-cas 3 - 2

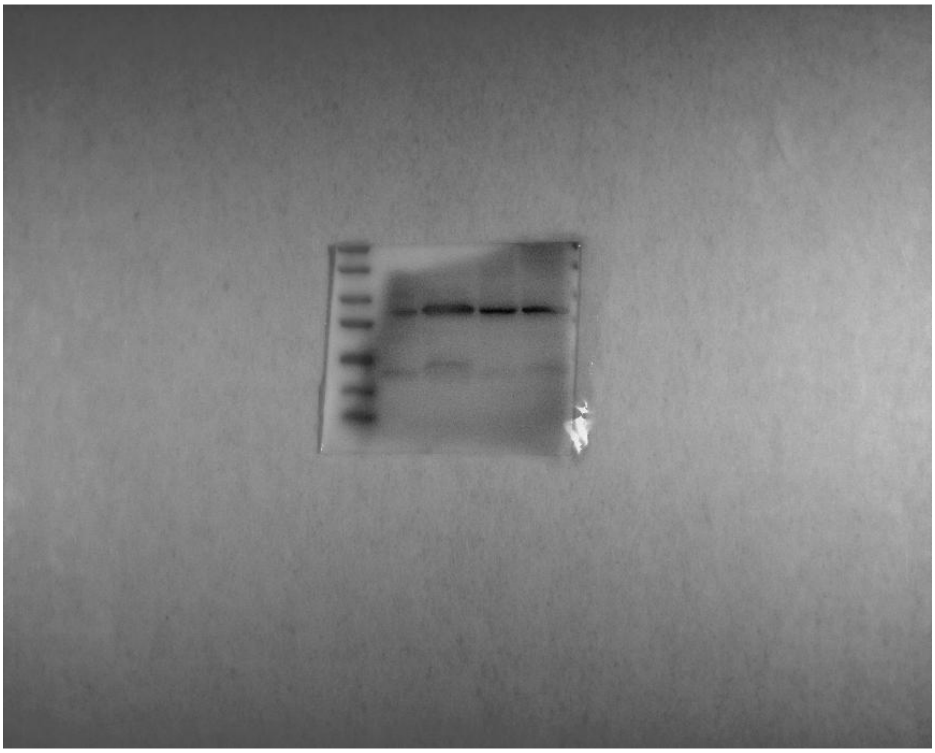

β - actin

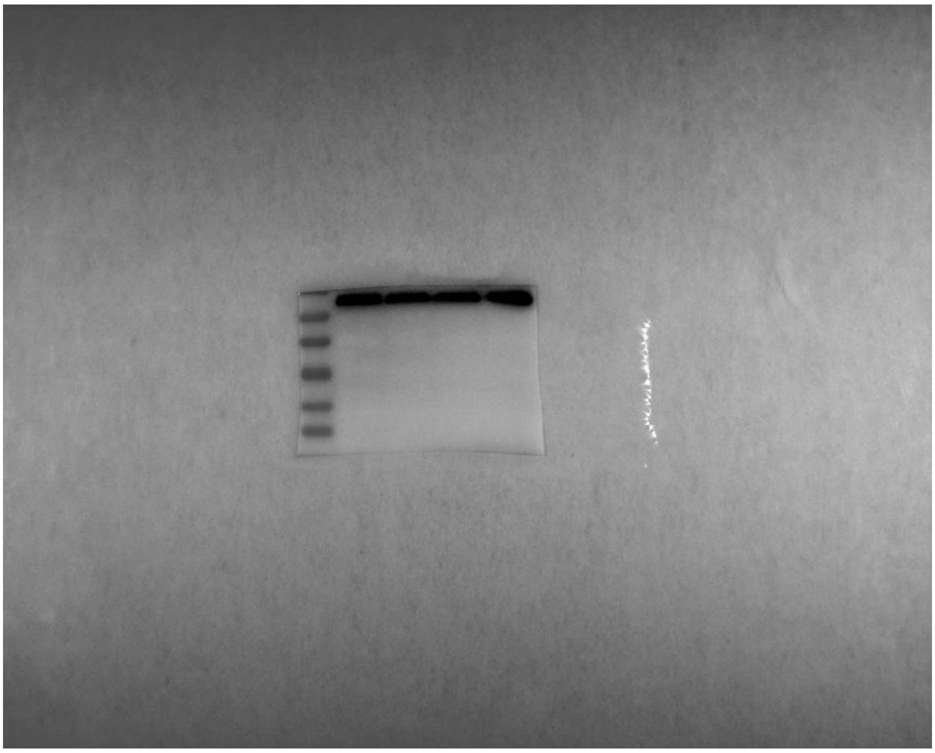

C-cas 3 - 3

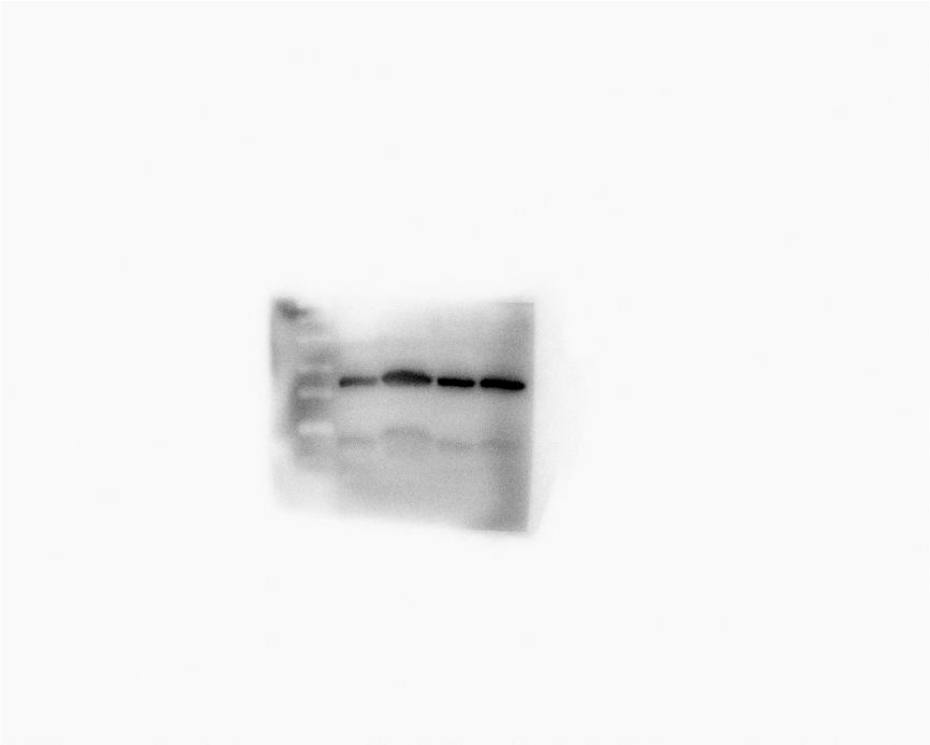

β - actin

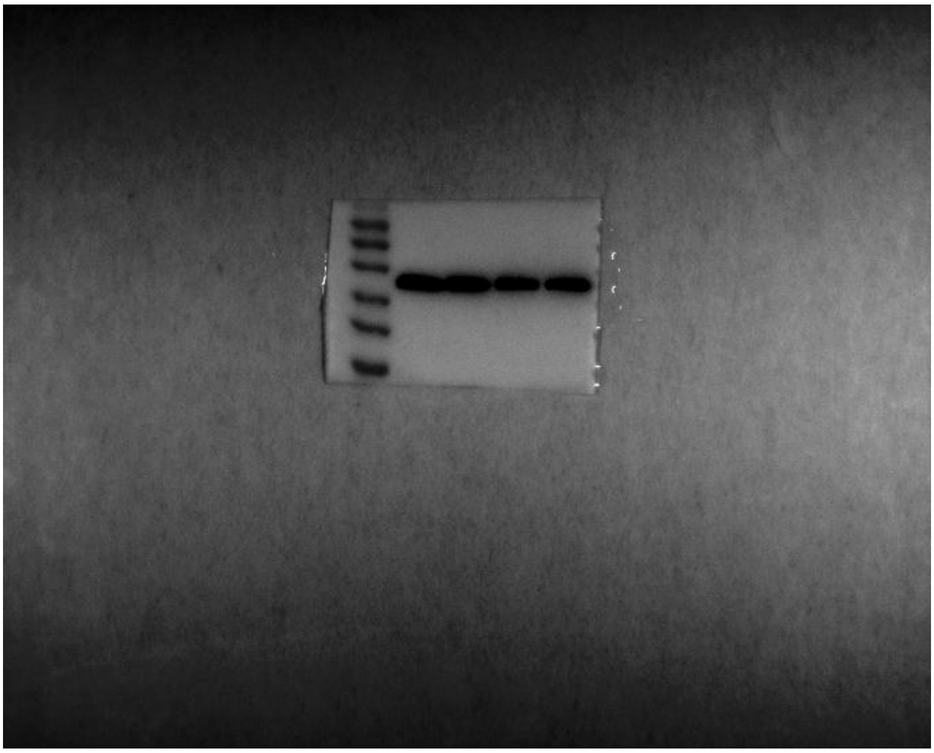

C-cas 9 - 1

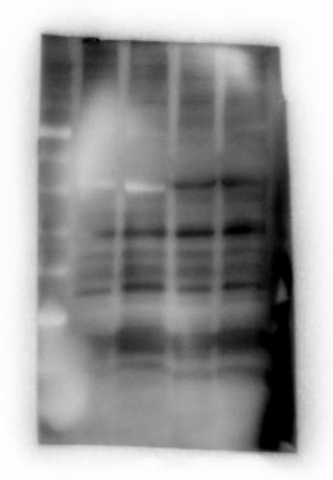

β - actin

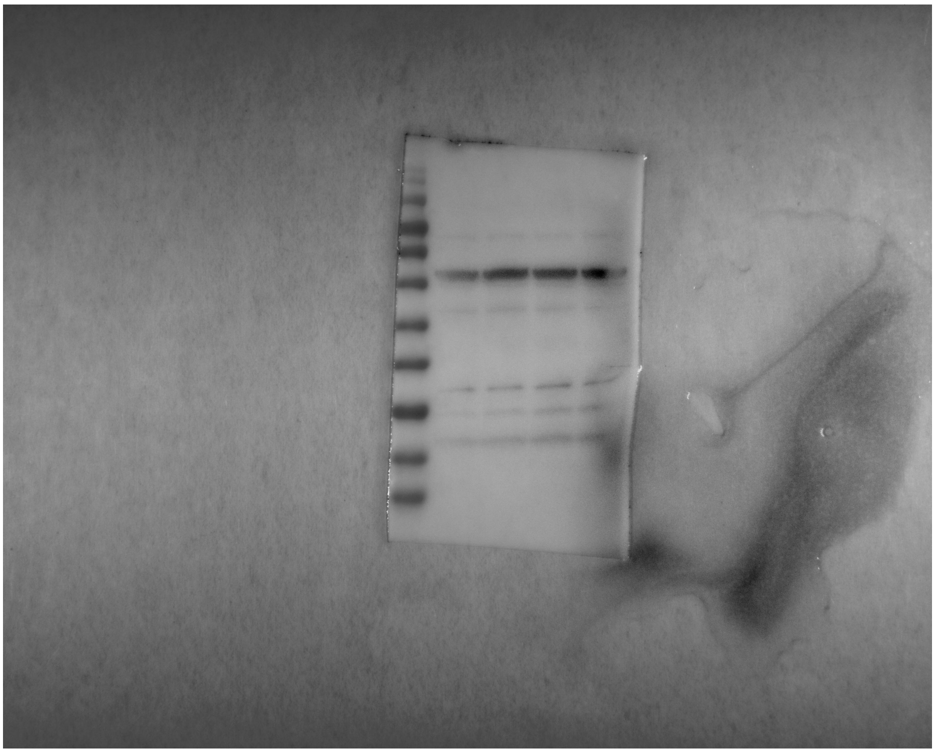

C-cas 9 -2

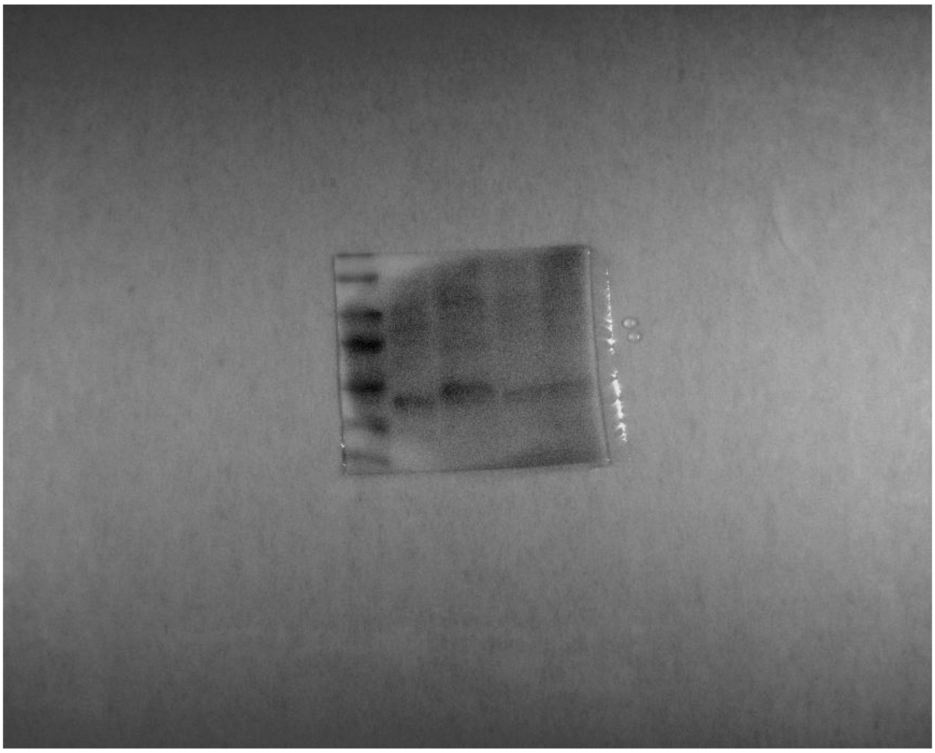

β - actin

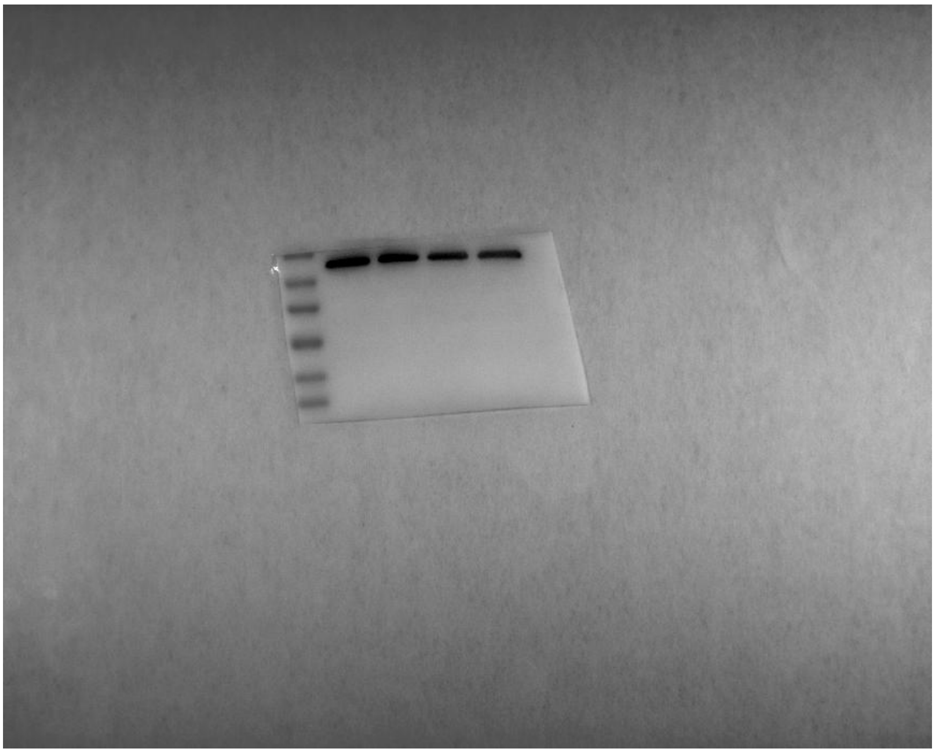

C-cas 9 -3

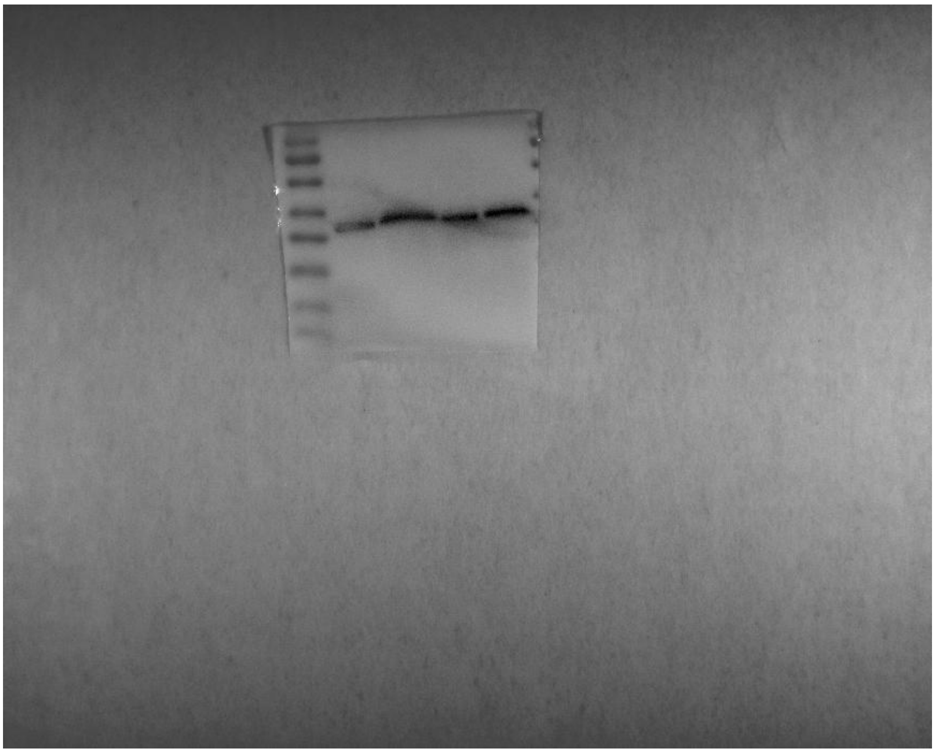

β - actin

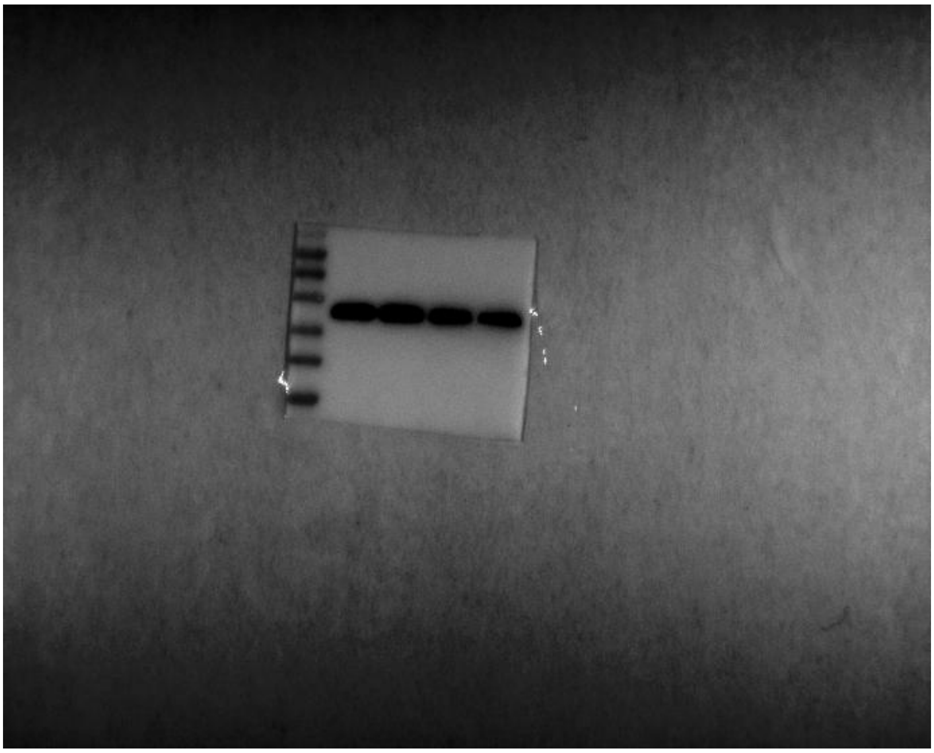

P53 - 1

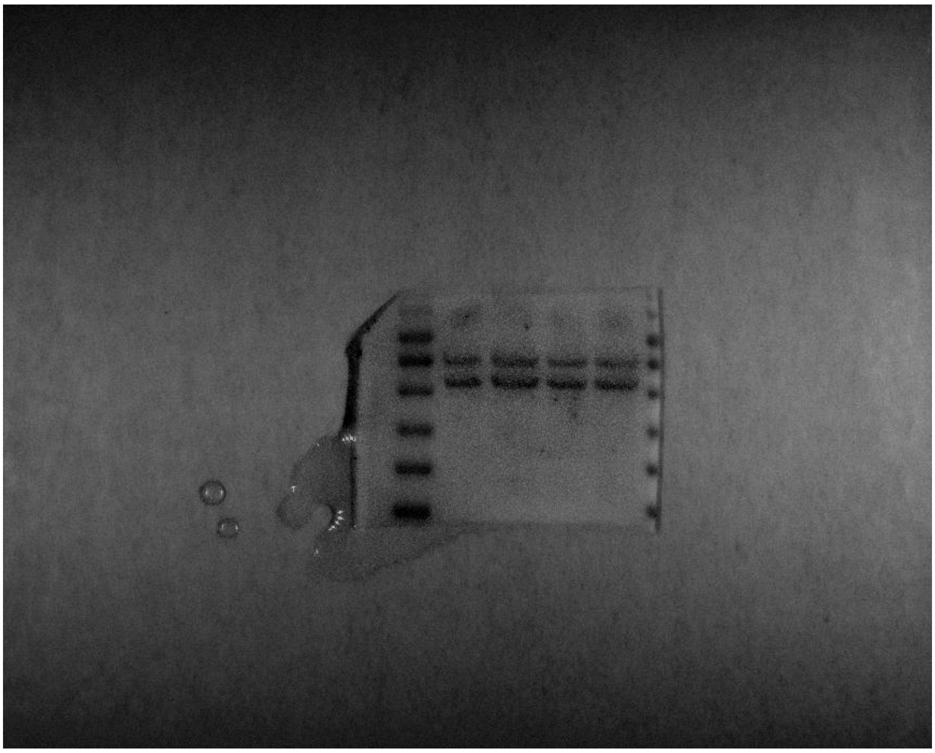

β - actin

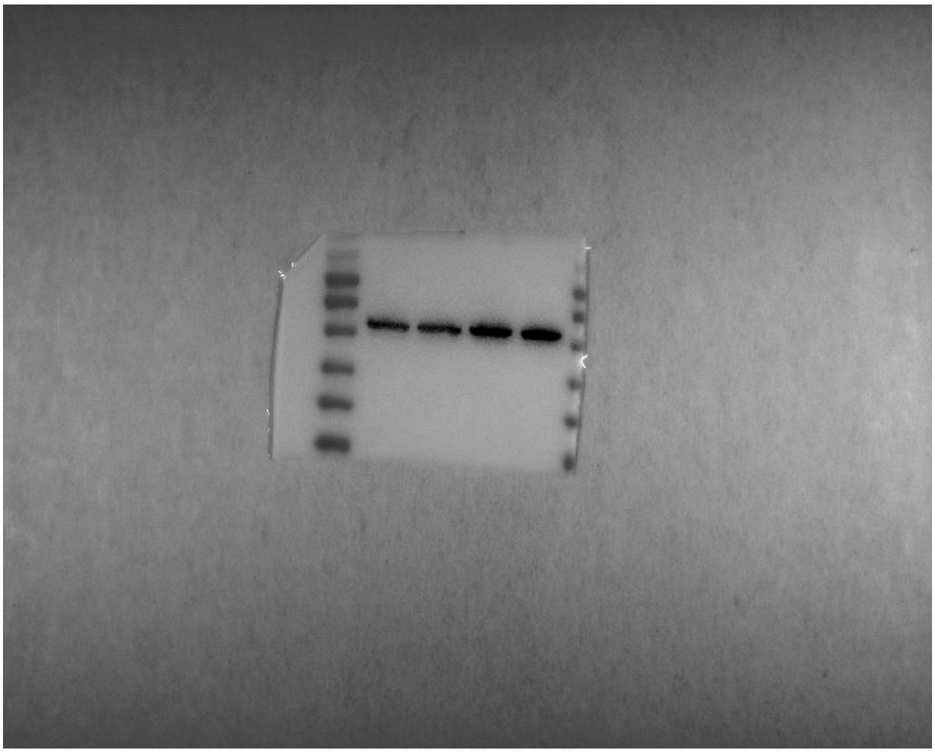

P53 - 2

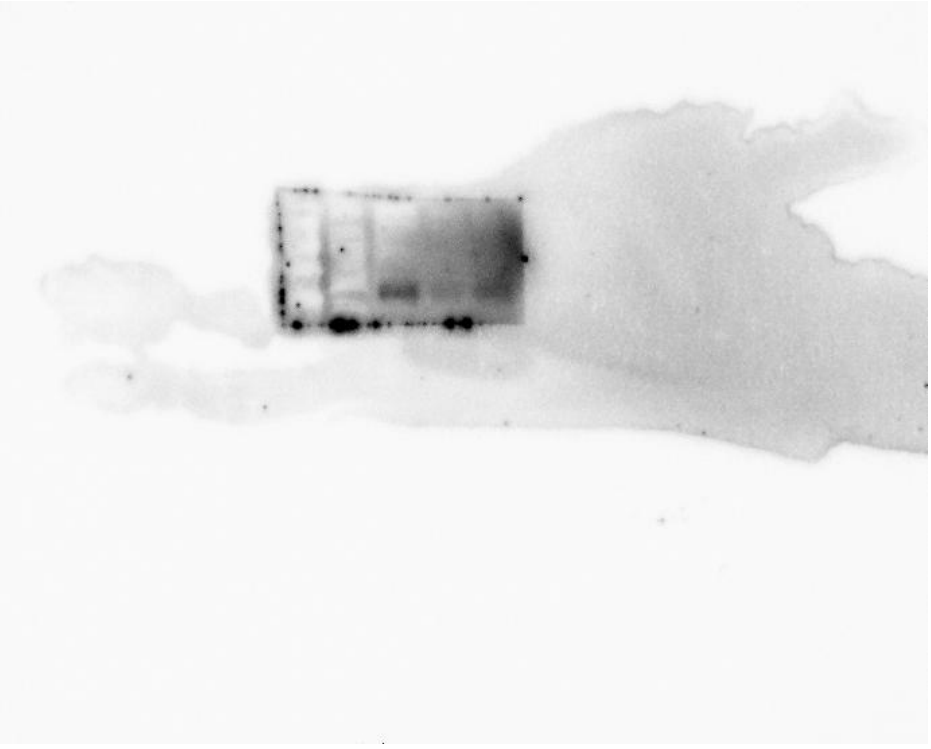

β - actin

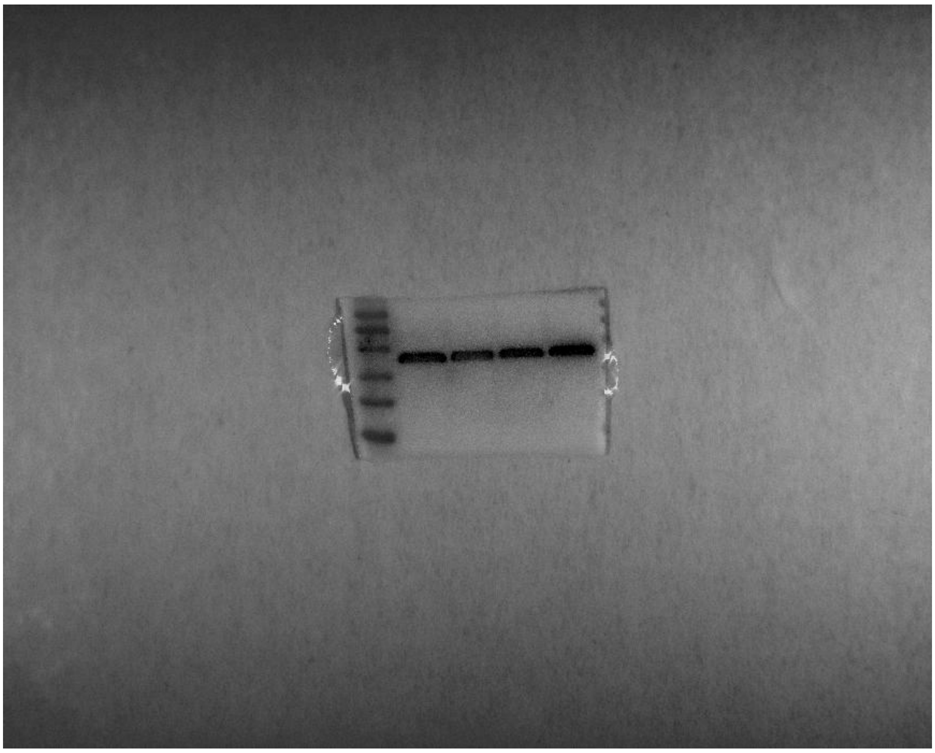

P53 - 3

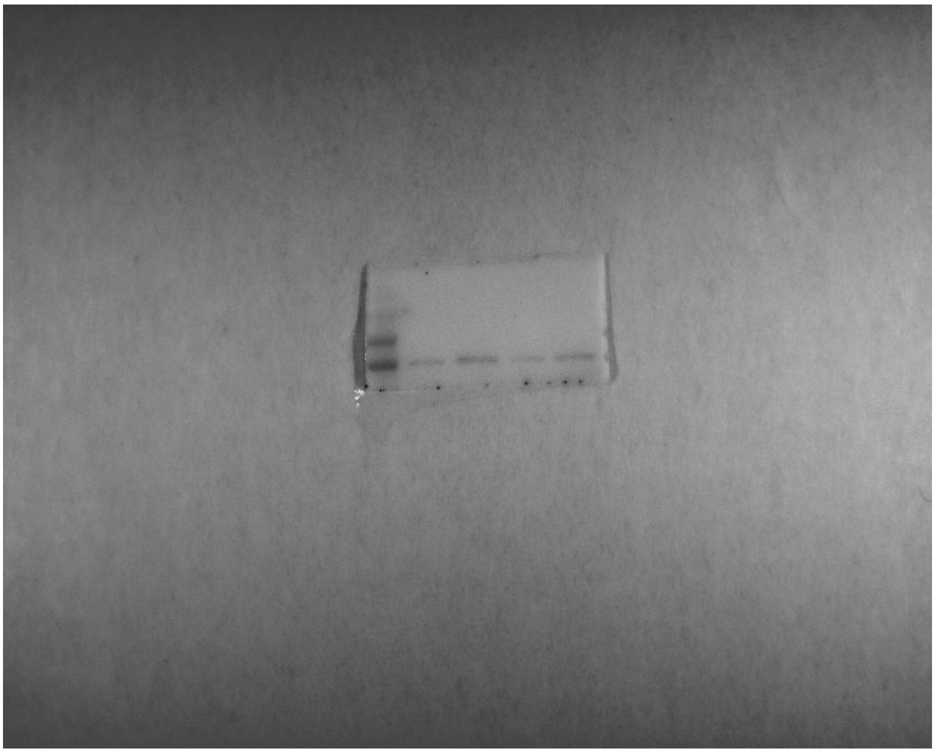

β - actin

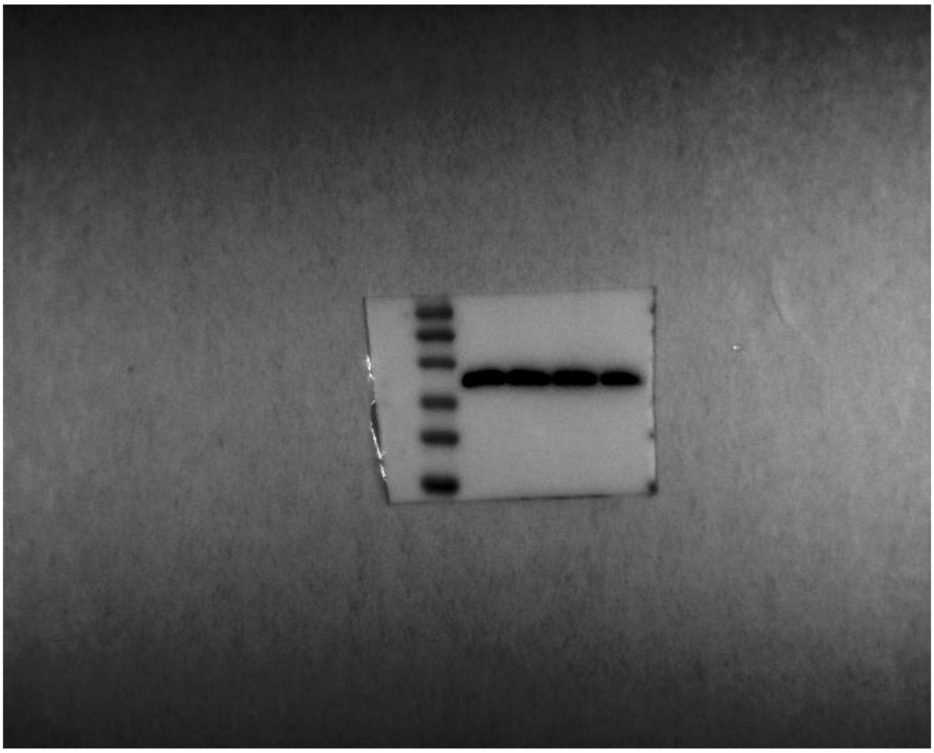

AKT - 1

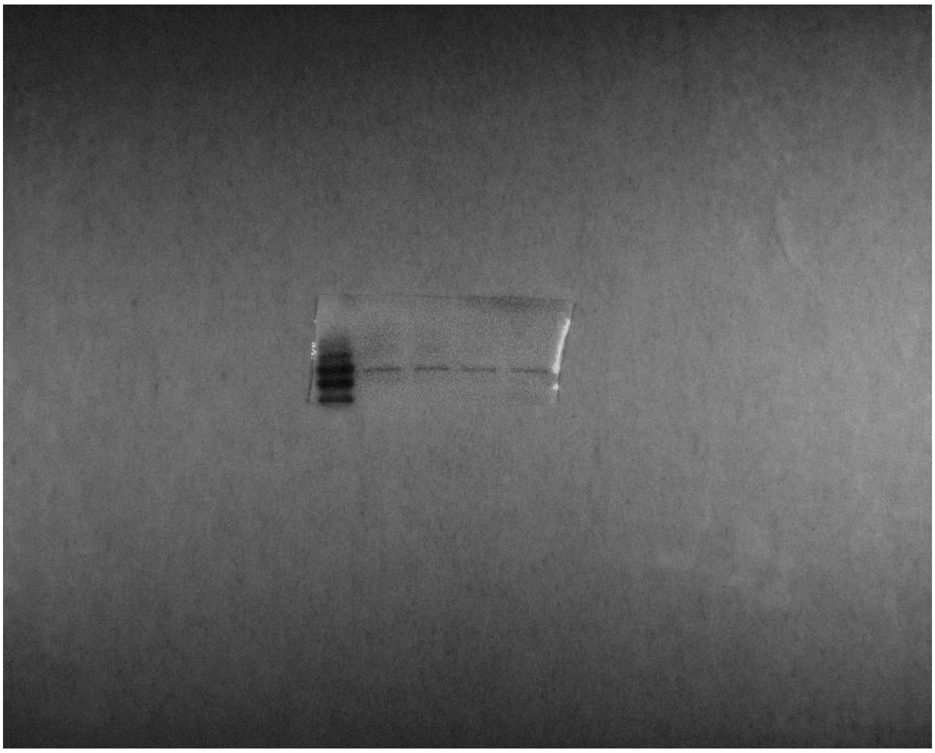

β - actin

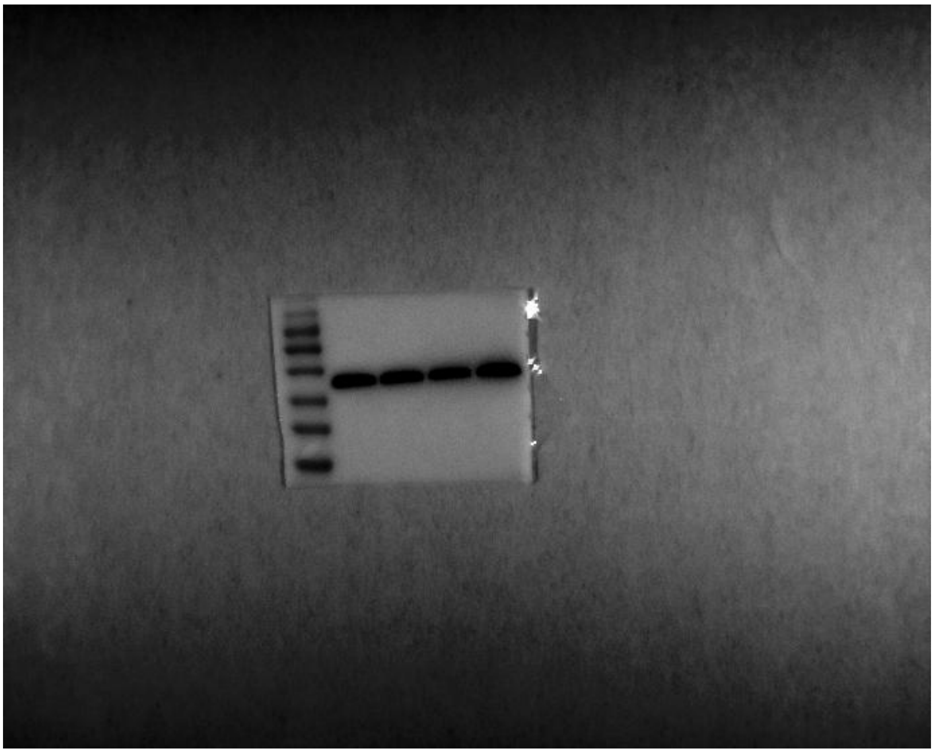

AKT - 2

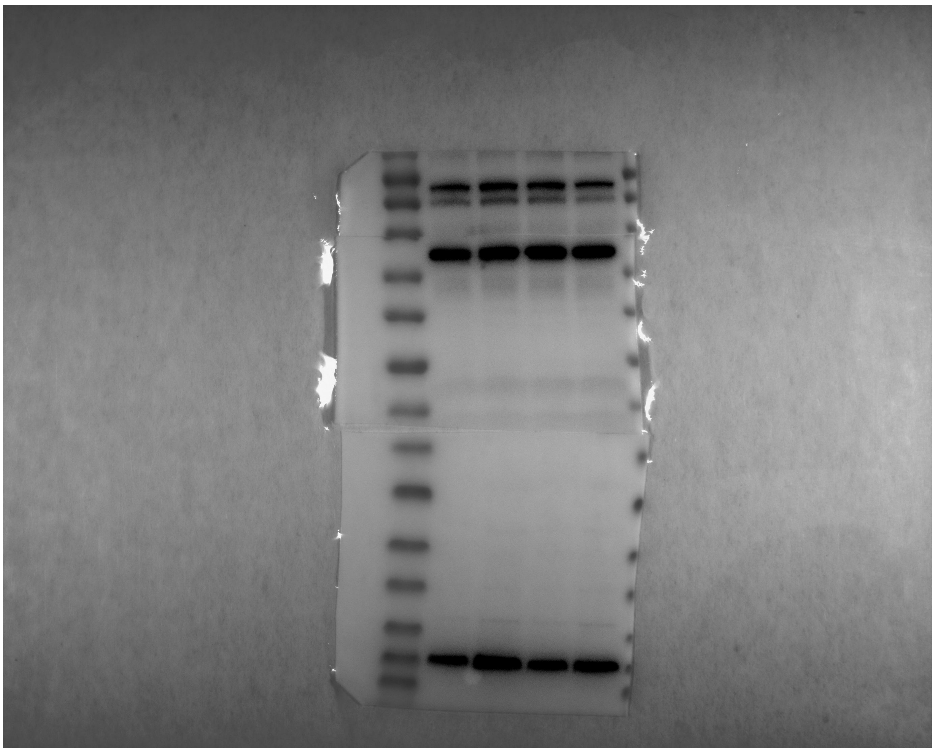

The top blot

β - actin

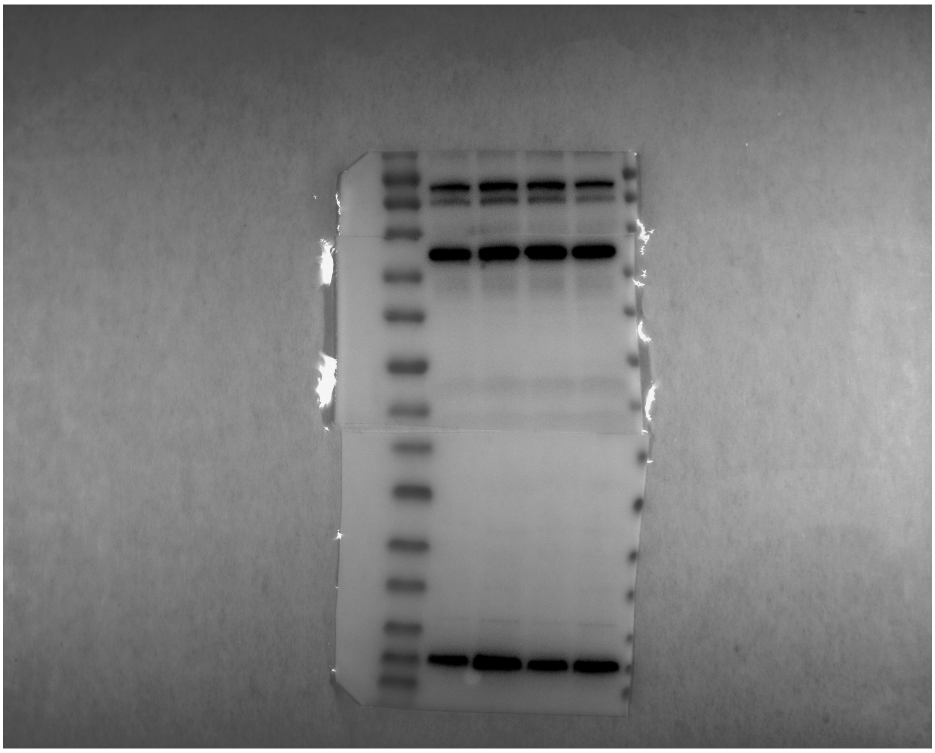

The middle blot

AKT - 3

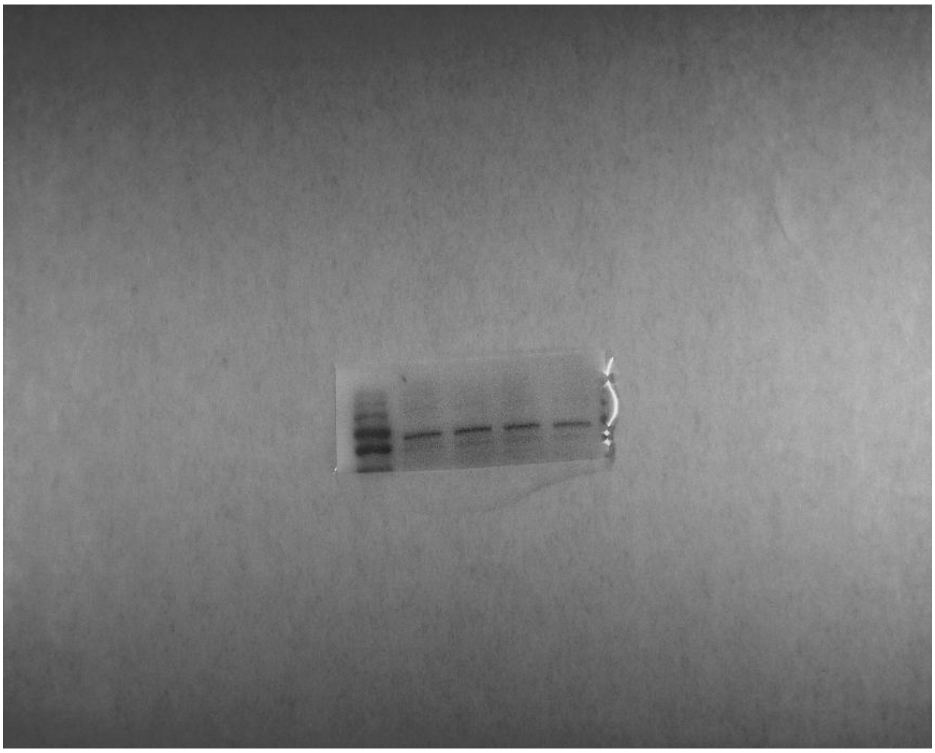

β - actin

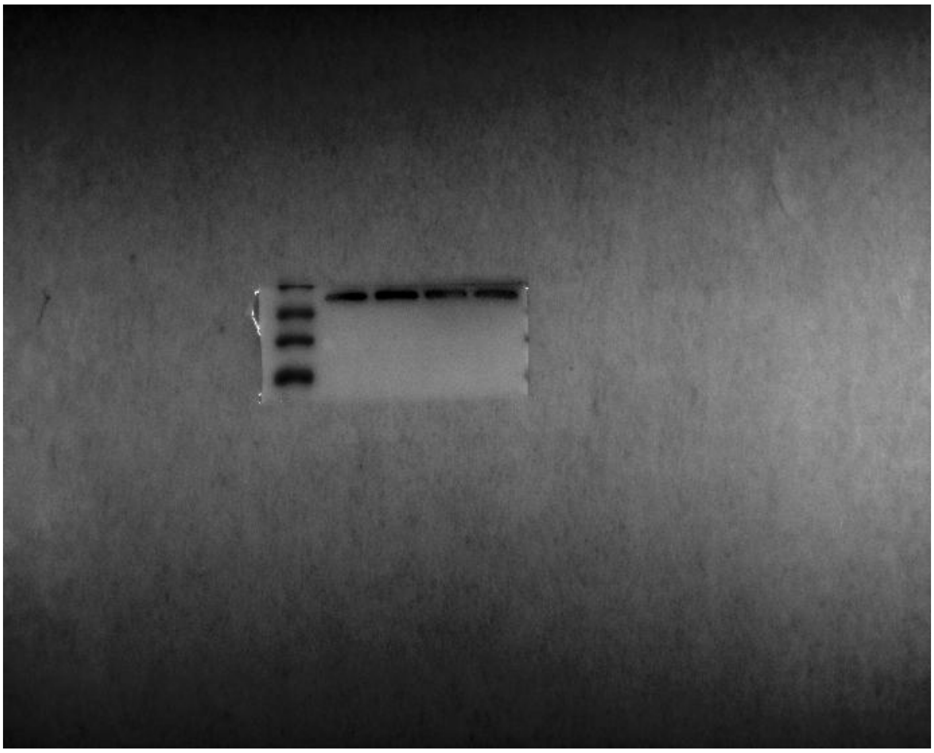

P-AKT - 1

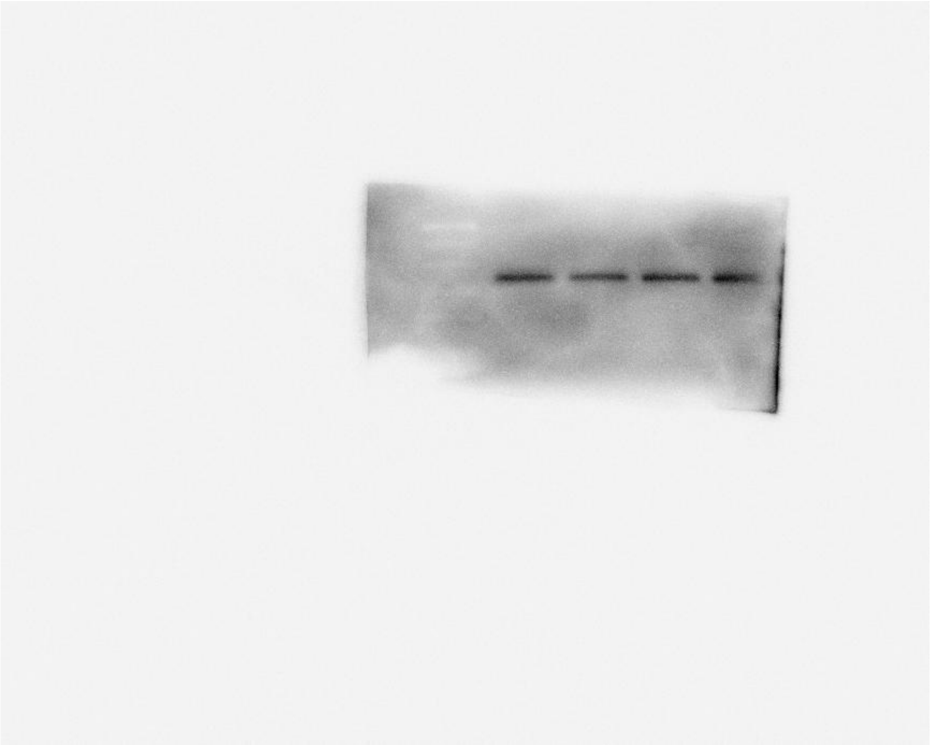

β - actin

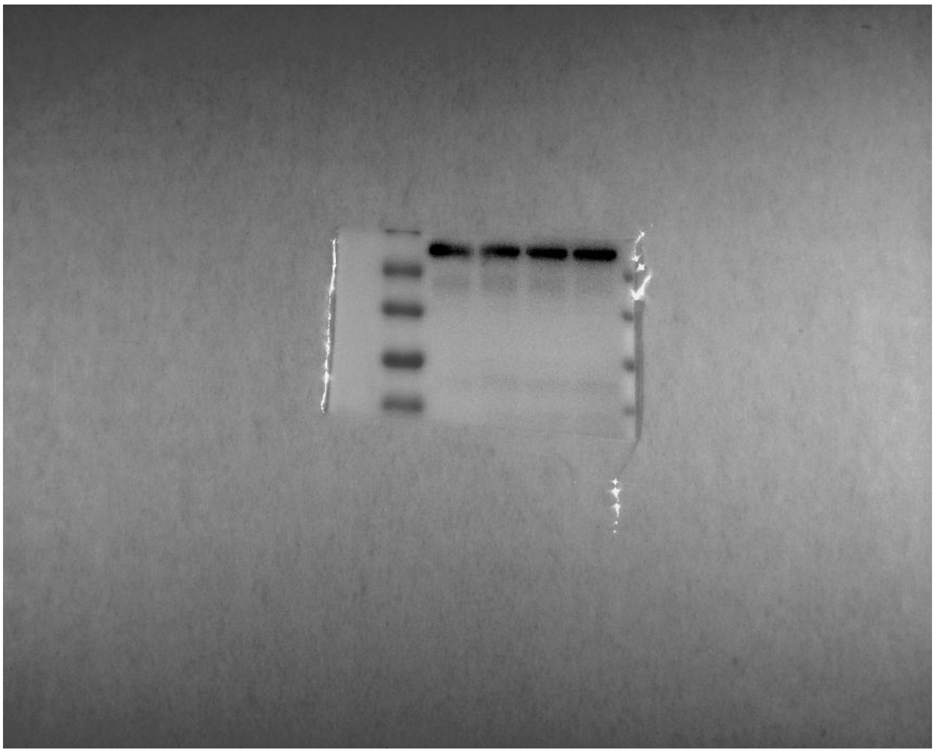

P-AKT - 2

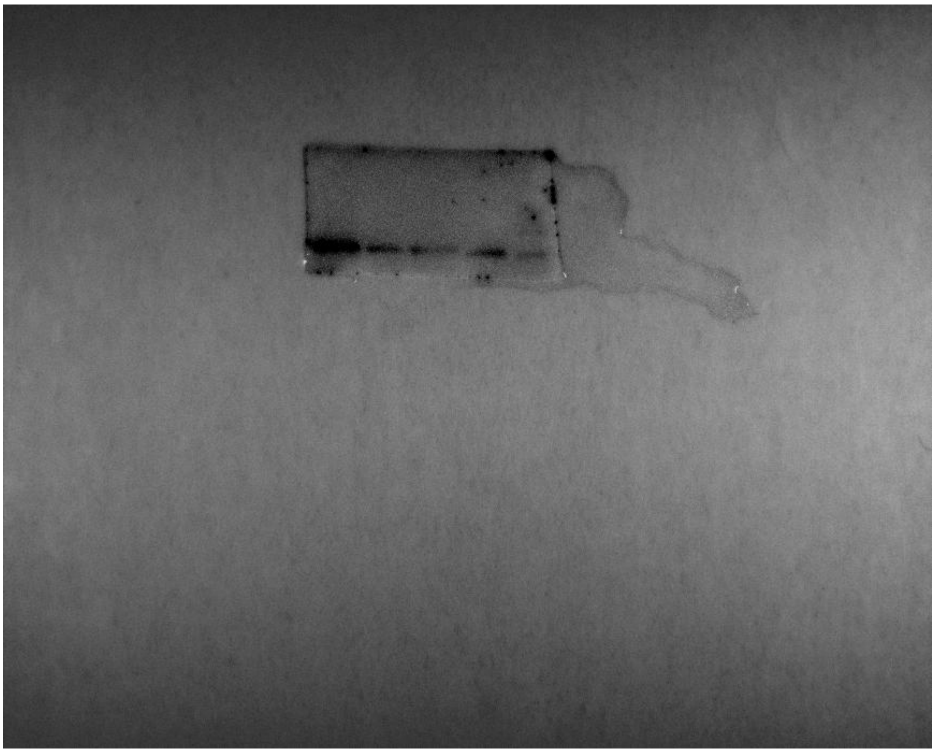

β - actin

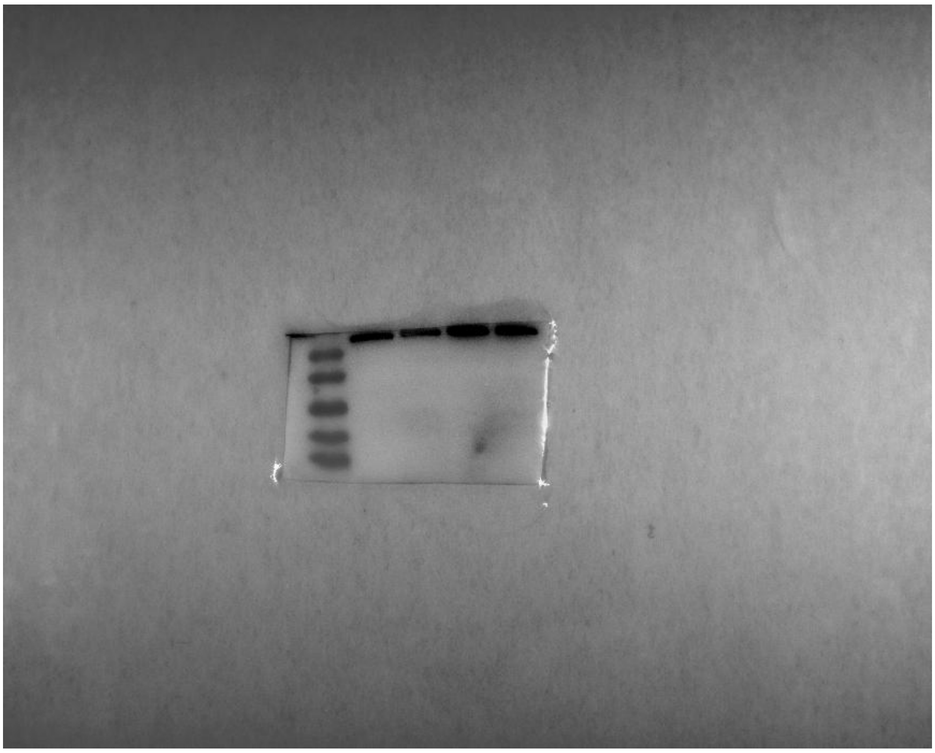

P-AKT - 3

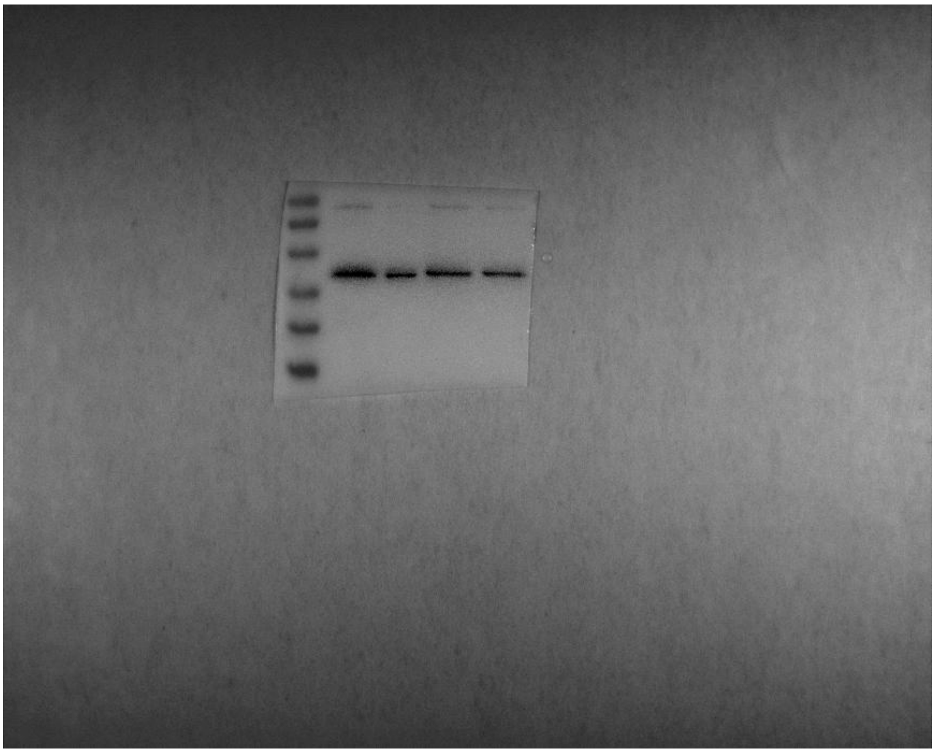

β - actin

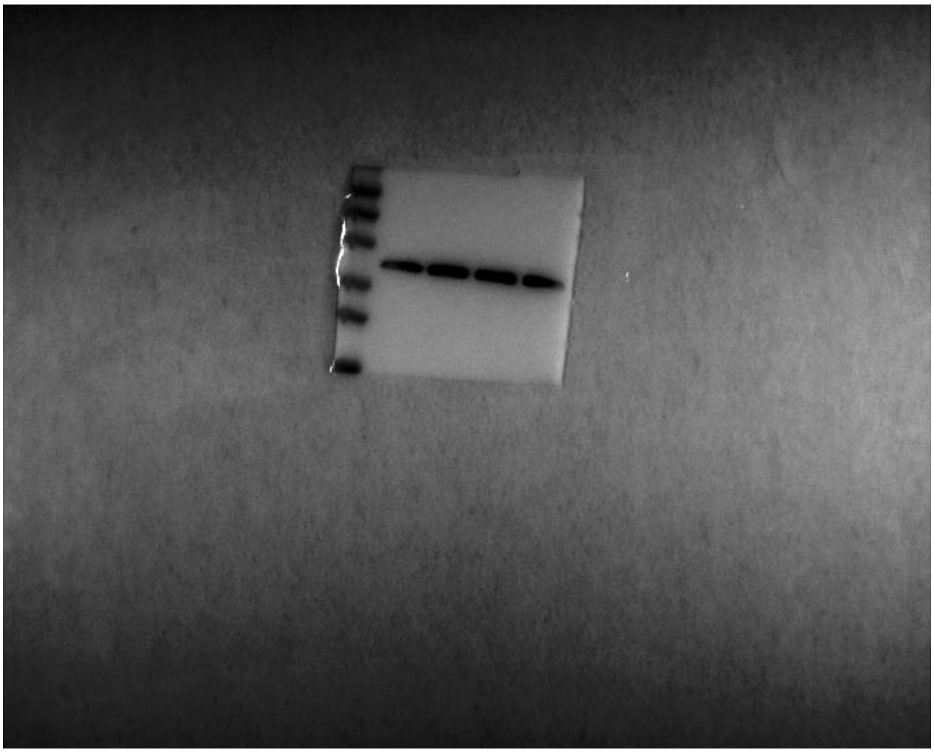

Western blot in cell

From left to right is CTR DOX DB SB
CTR : control
DOX : Doxorubicin
DB : Doxorubicin + Sodium butyrate
SB : Sodium butyrate

HDAC2 - 1

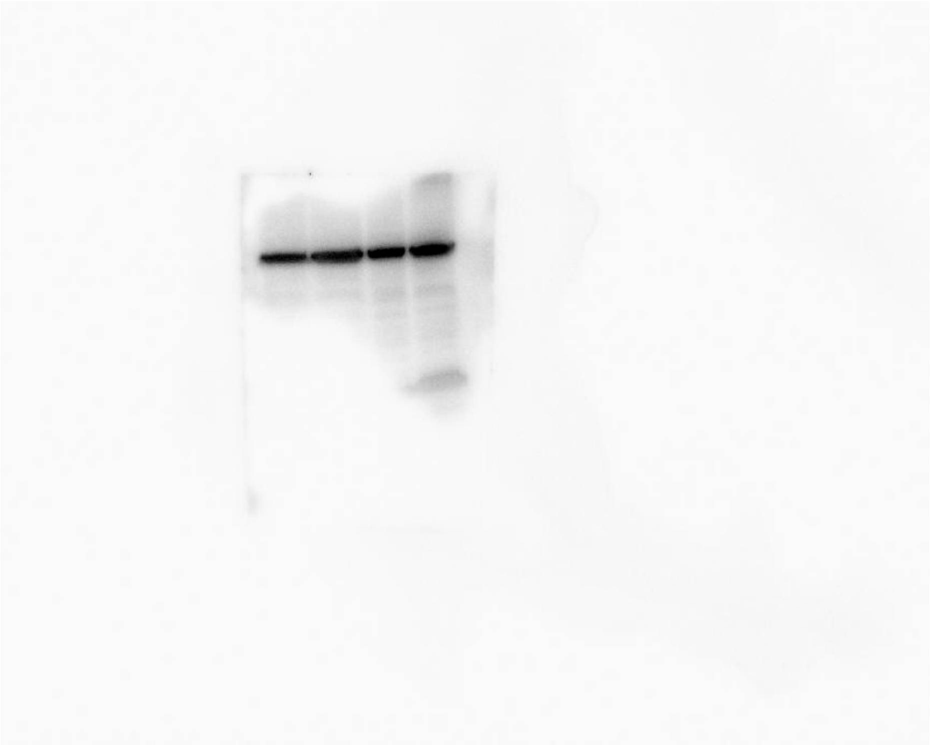

β - actin

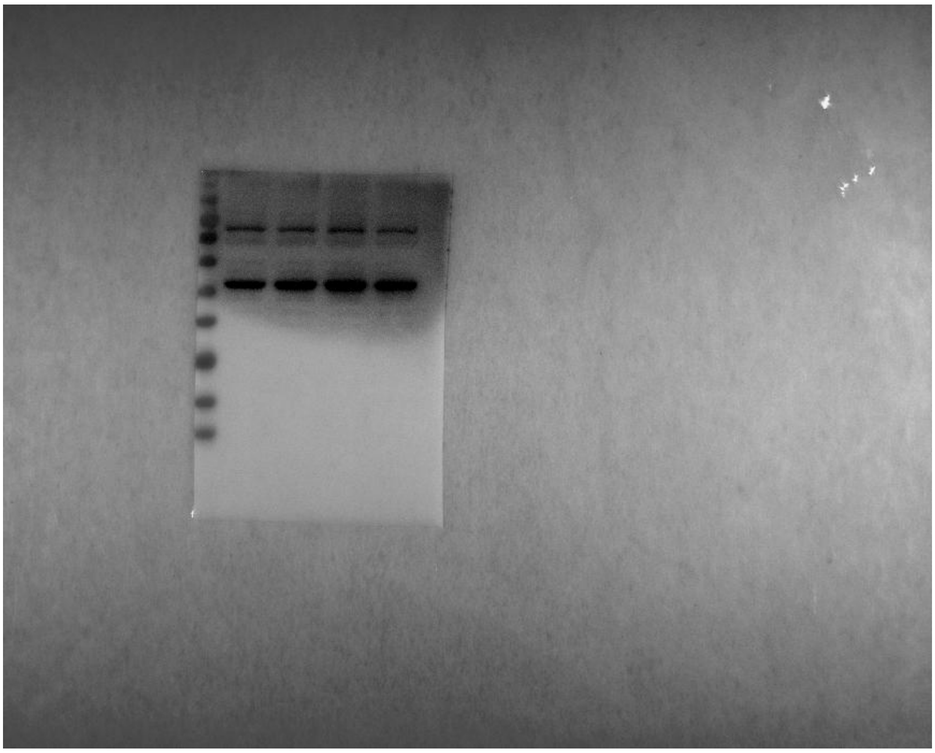

HDAC2 - 2

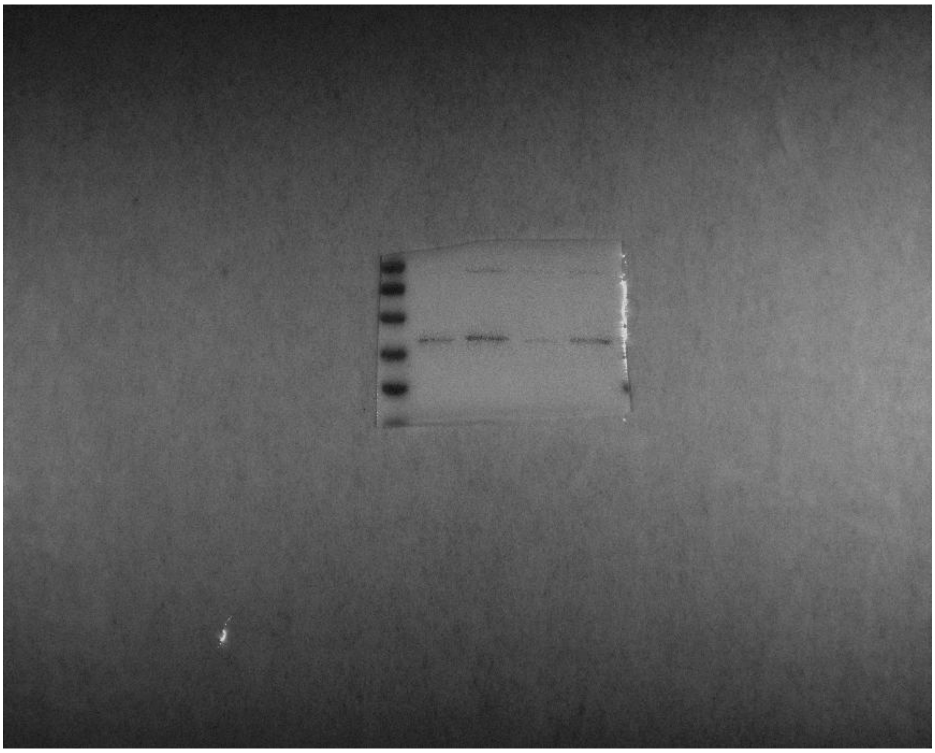

β - actin

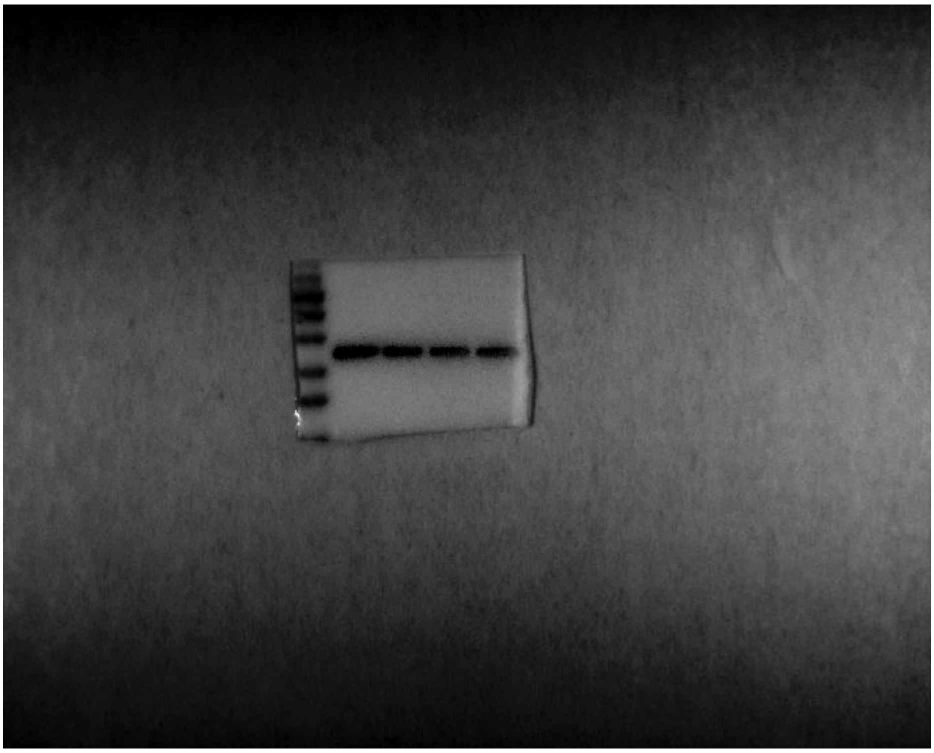

HDAC2 - 3

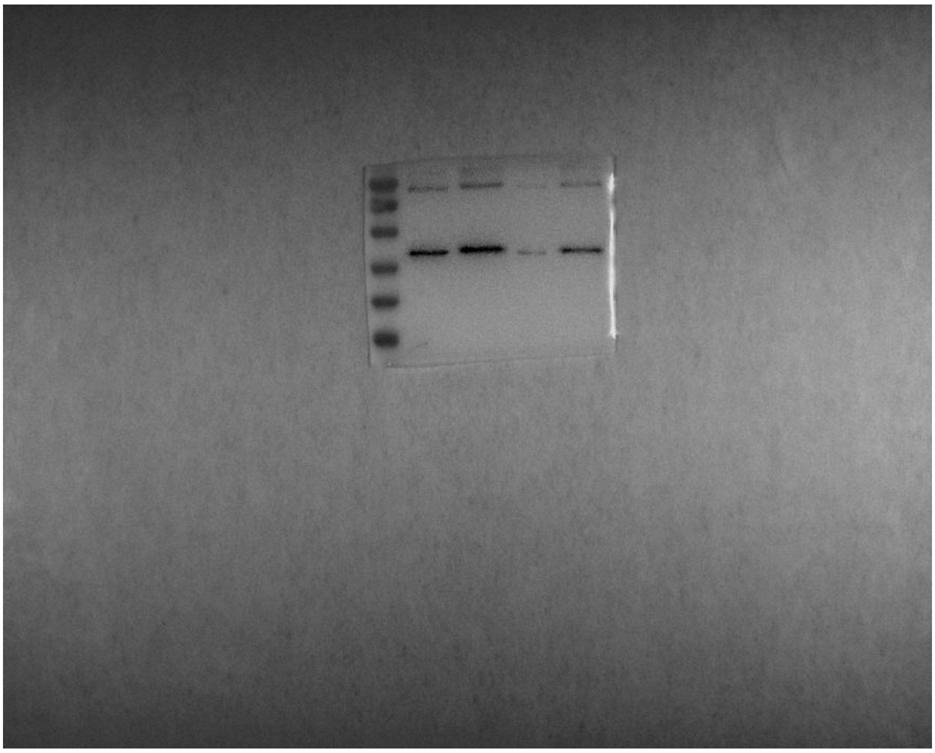

β - actin

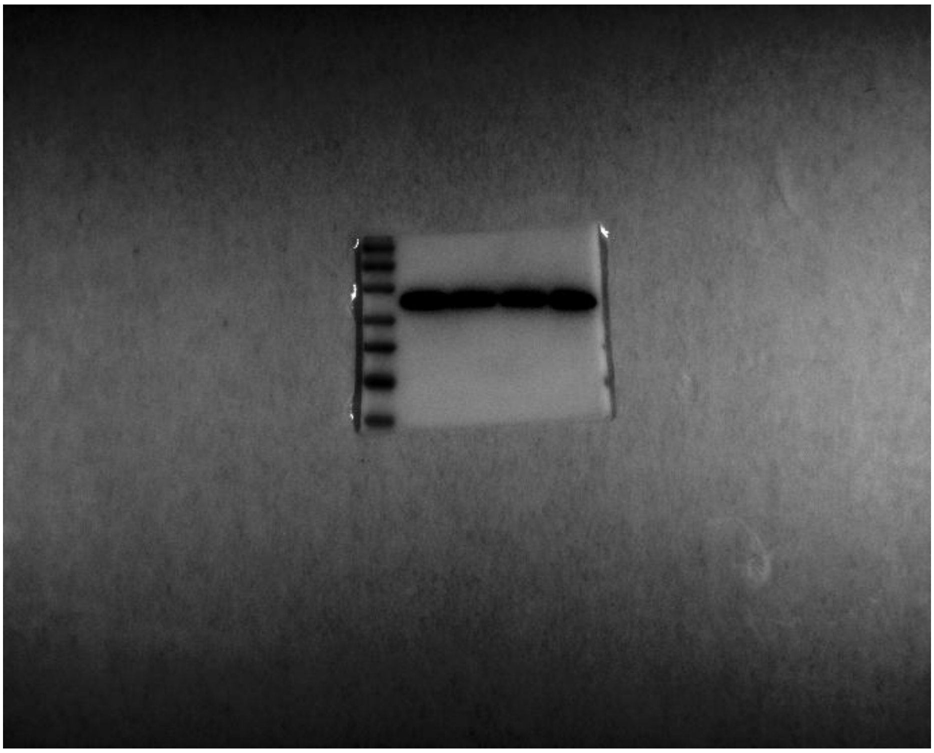

BAX - 1

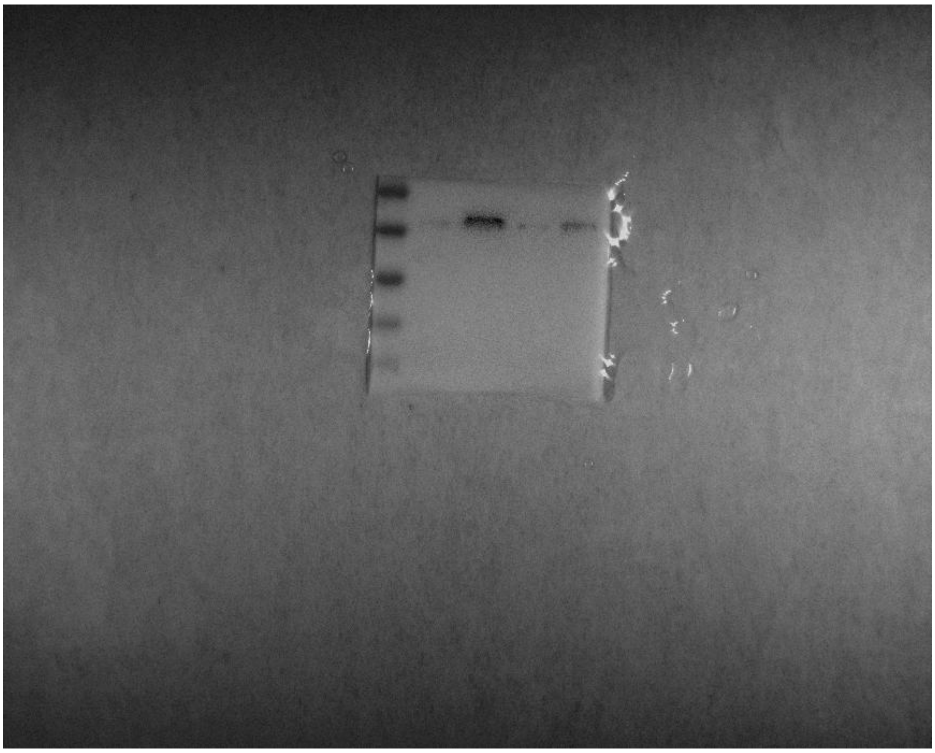

β - actin

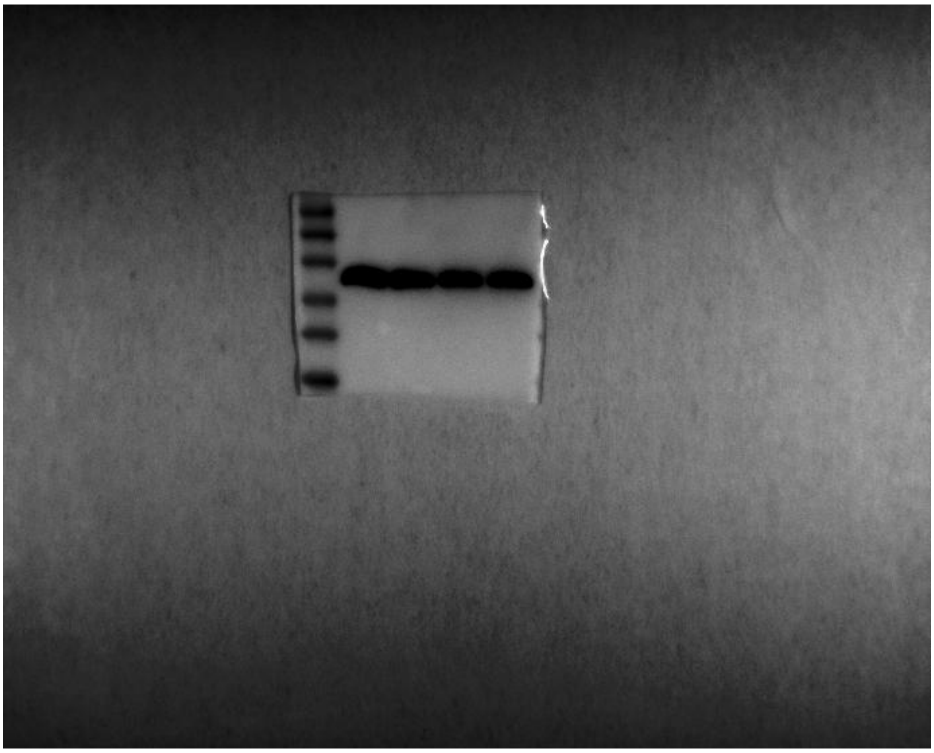

BAX - 2

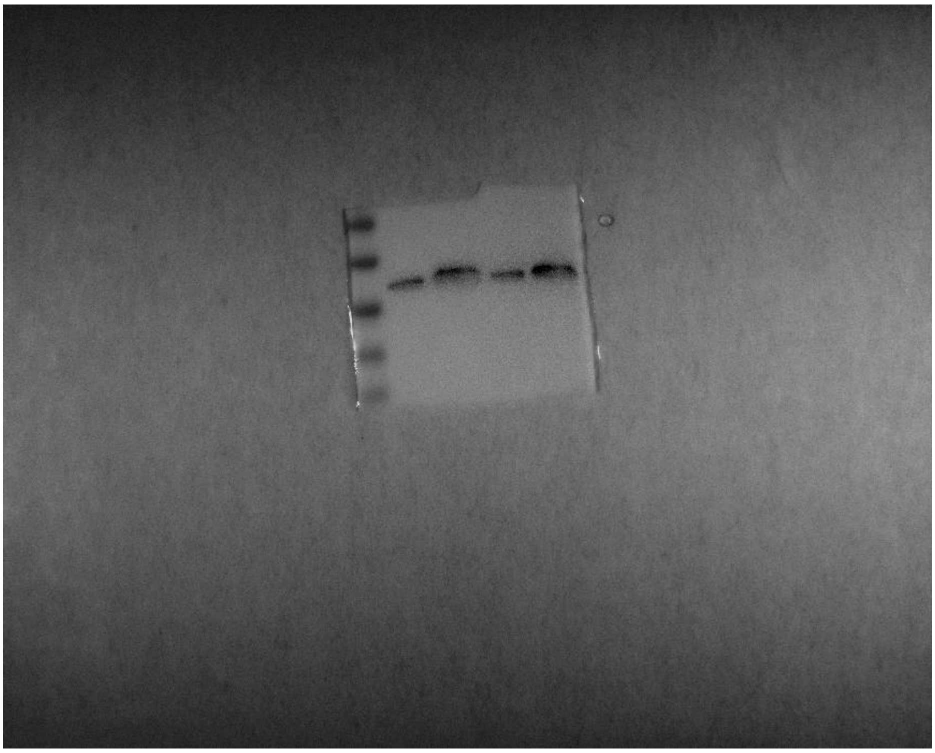

β - actin

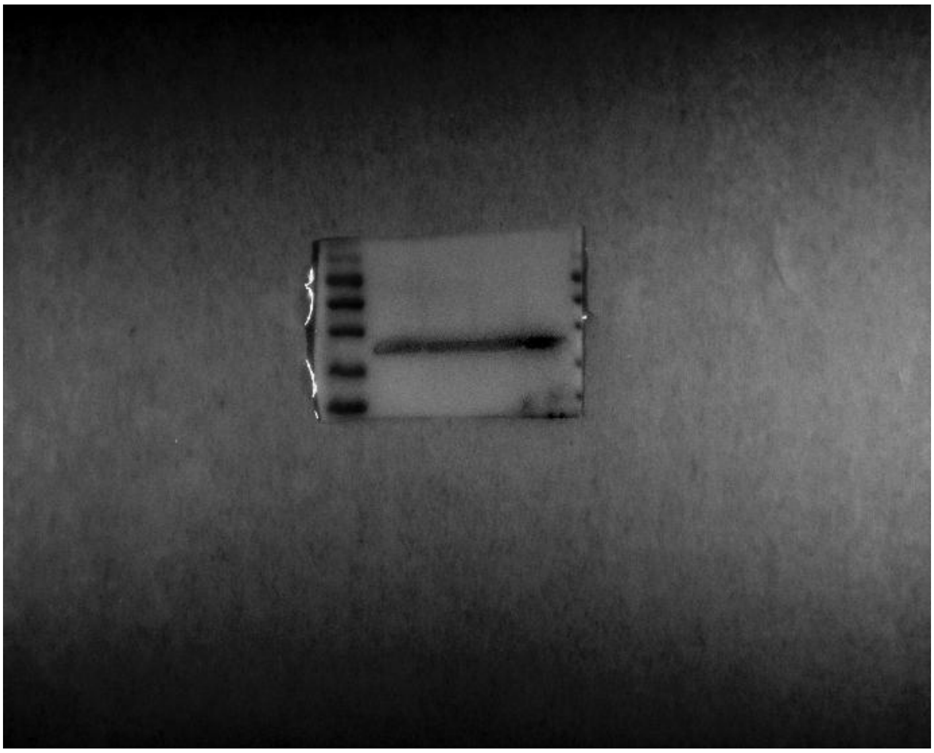

BAX - 3

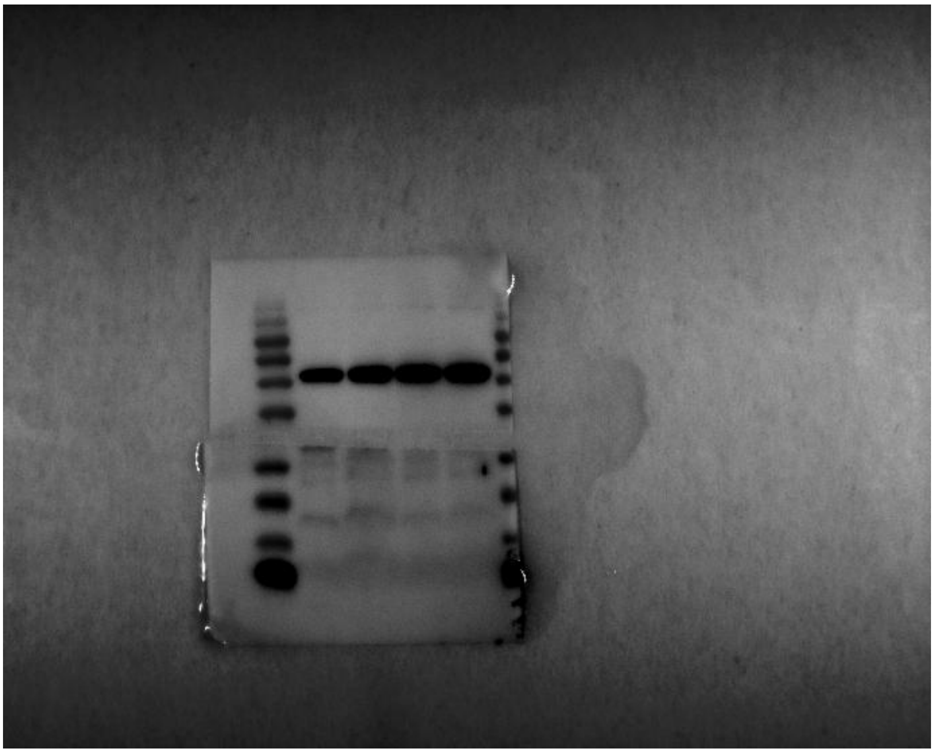

β - actin

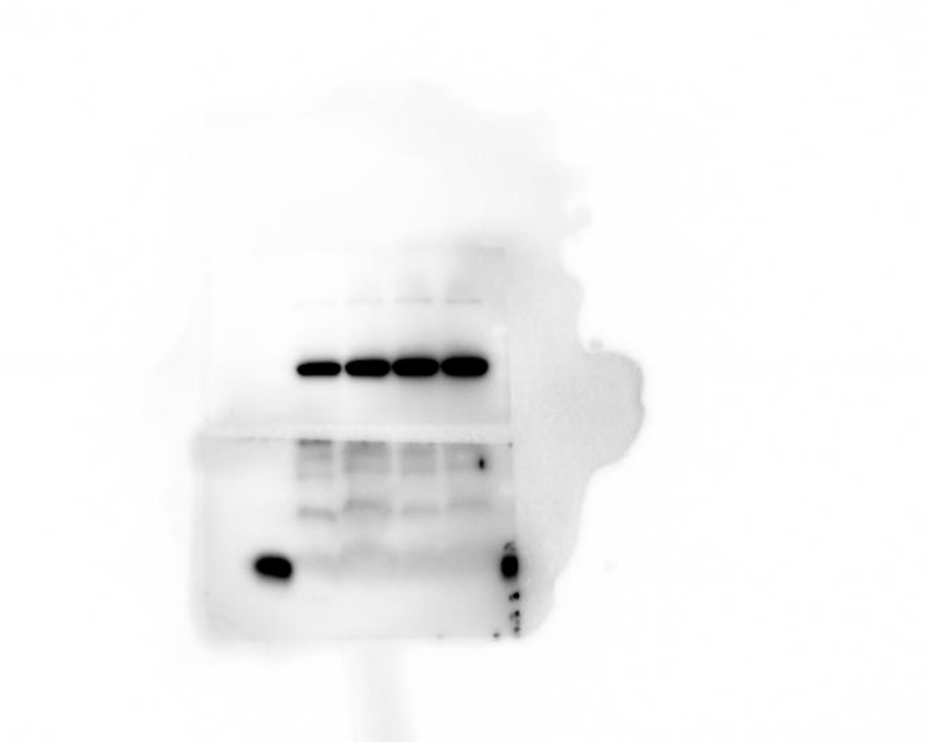

Bcl-2 - 1

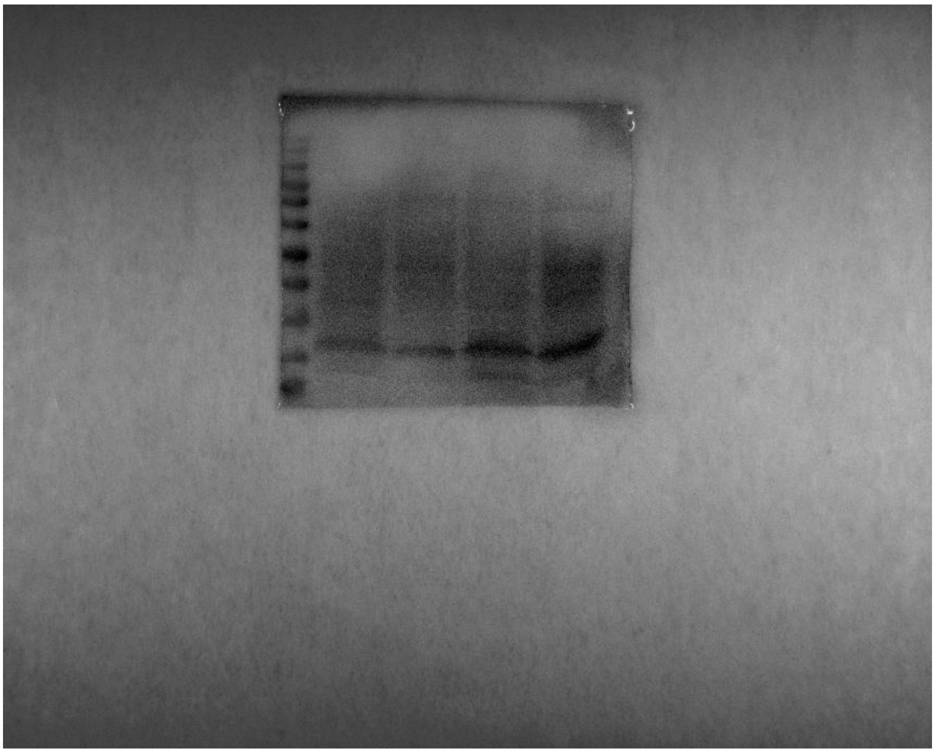

β - actin

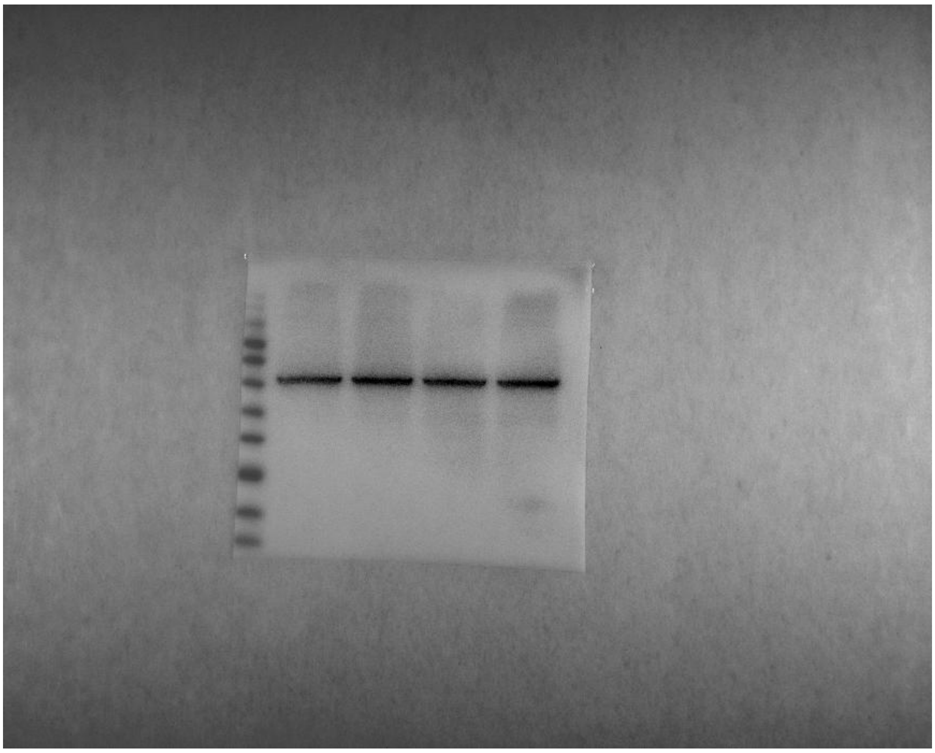

Bcl-2 - 2

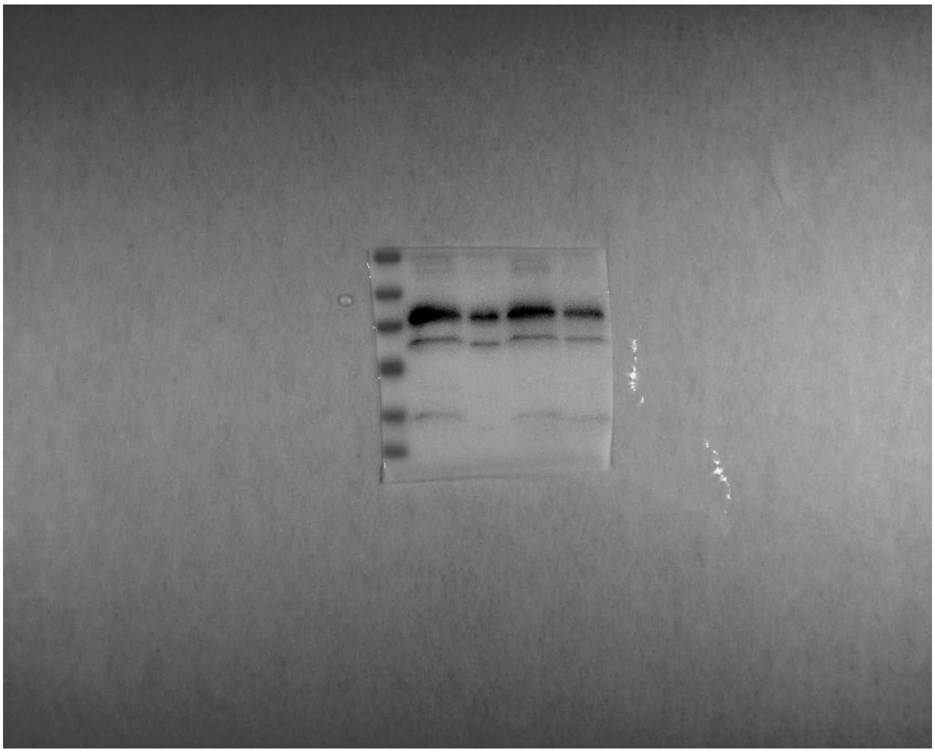

β - actin

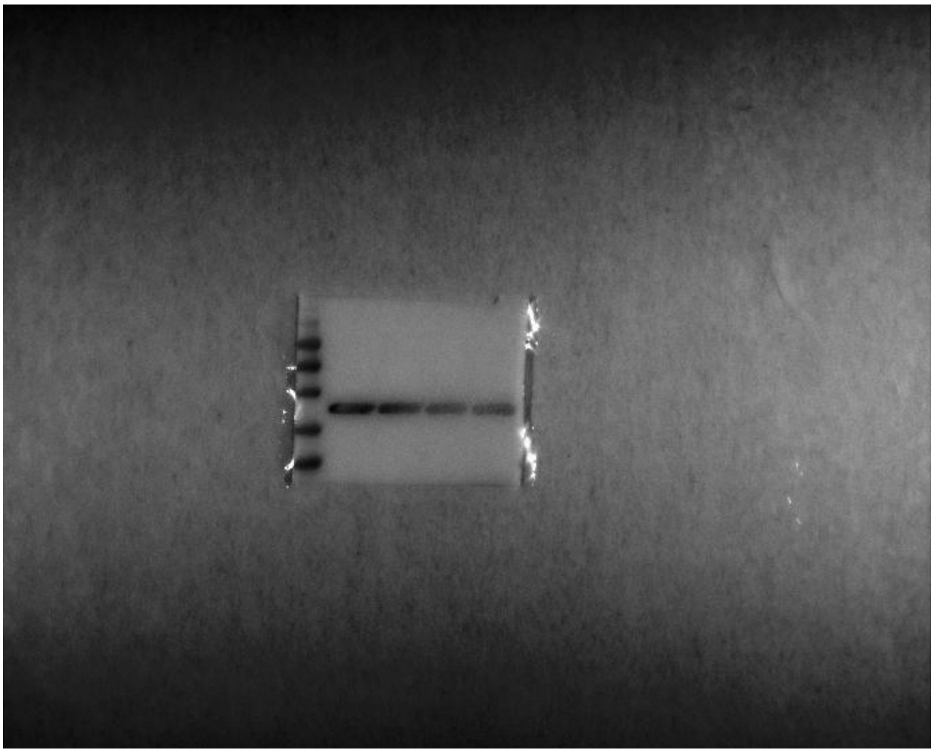

Bcl-2 - 3

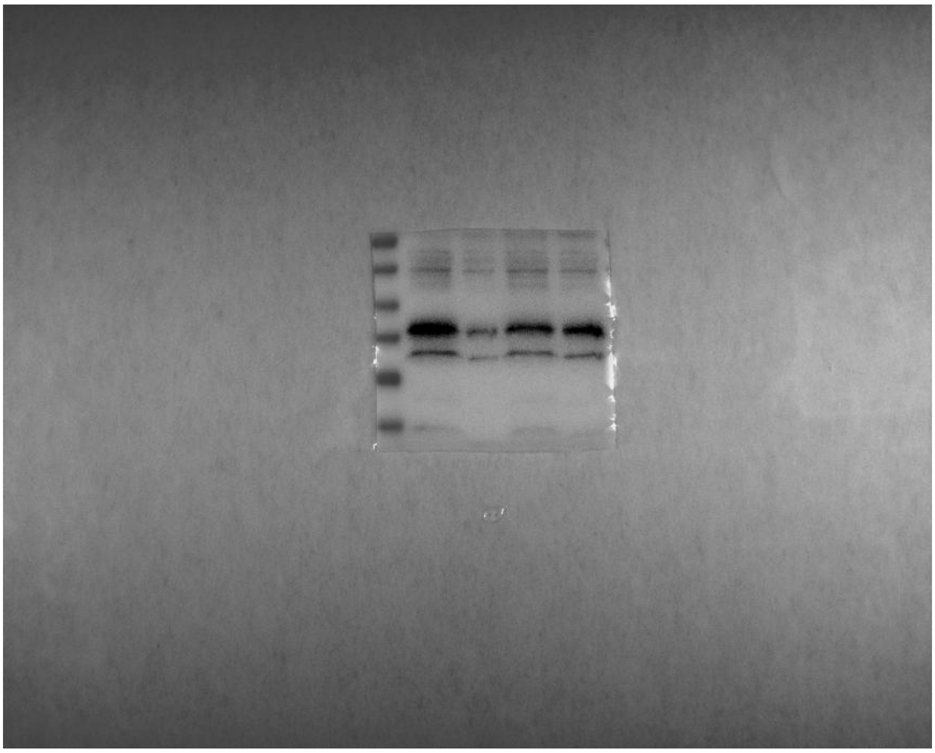

β - actin

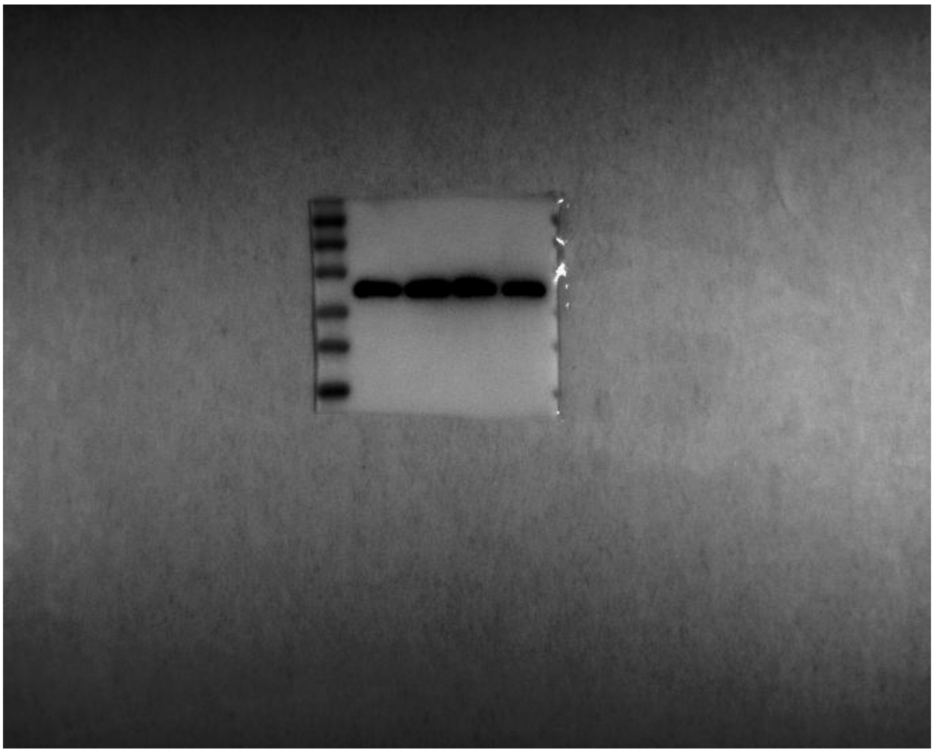

C-cas 3 -1

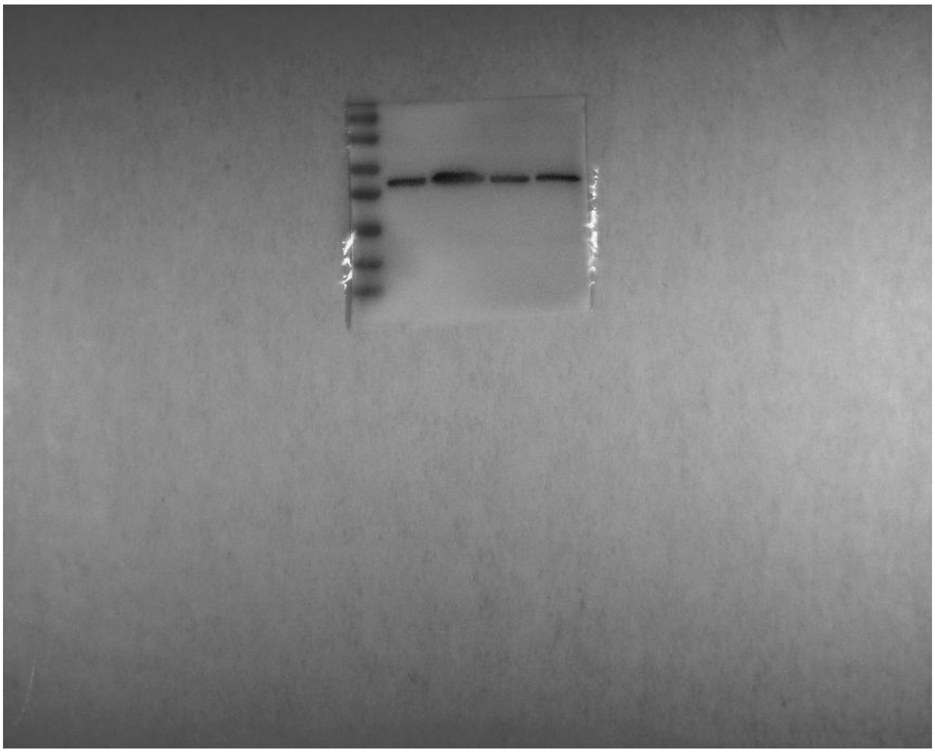

β - actin

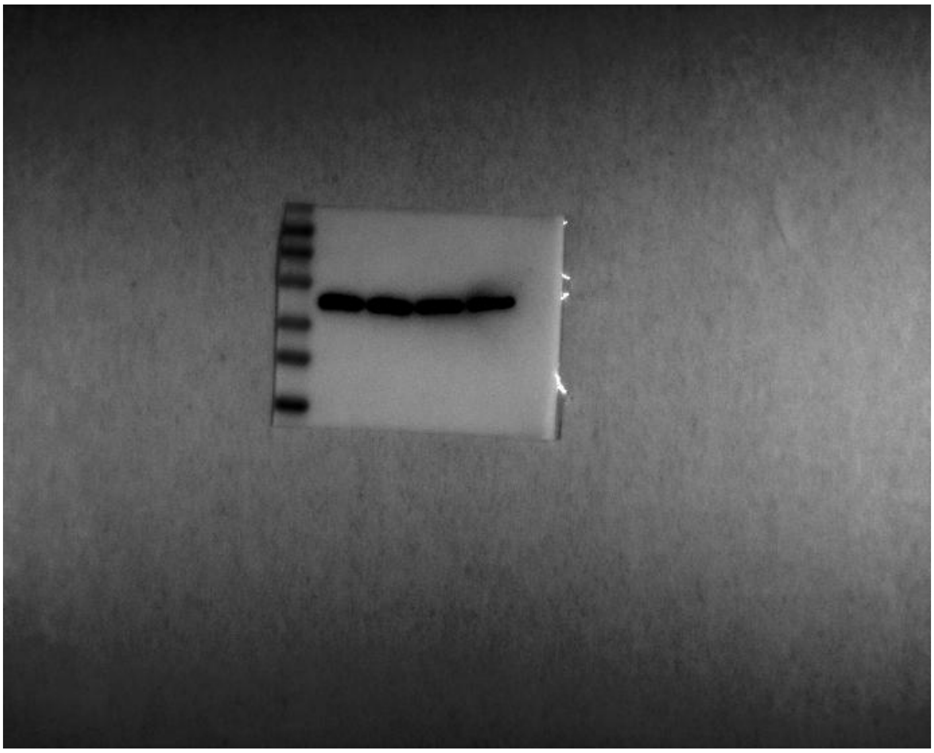

C-cas 3 - 2

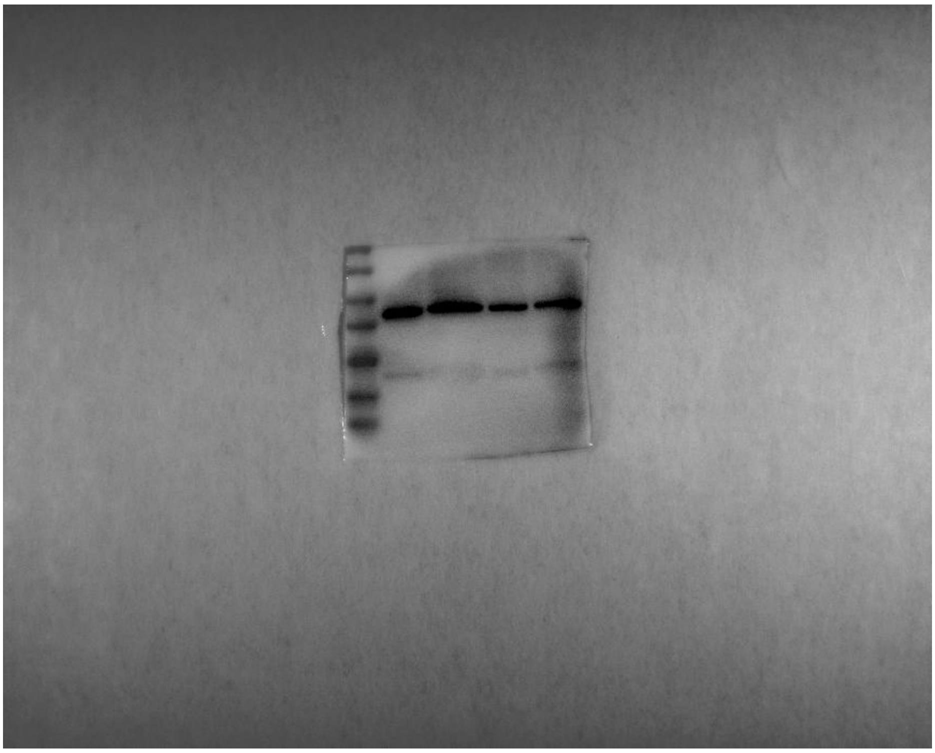

β - actin

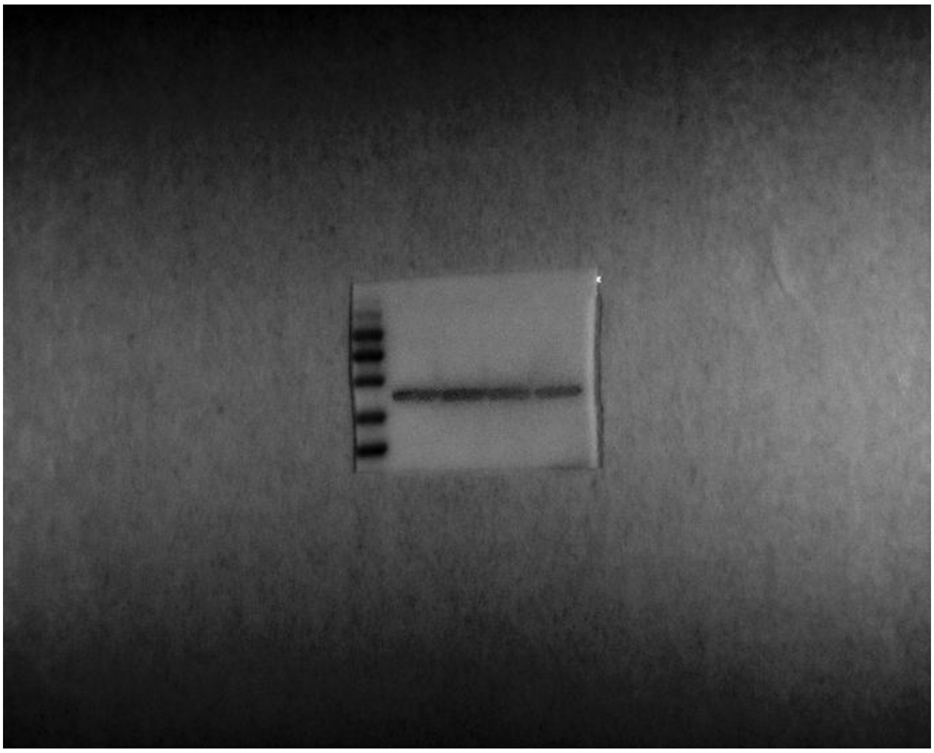

C-cas 3 - 3

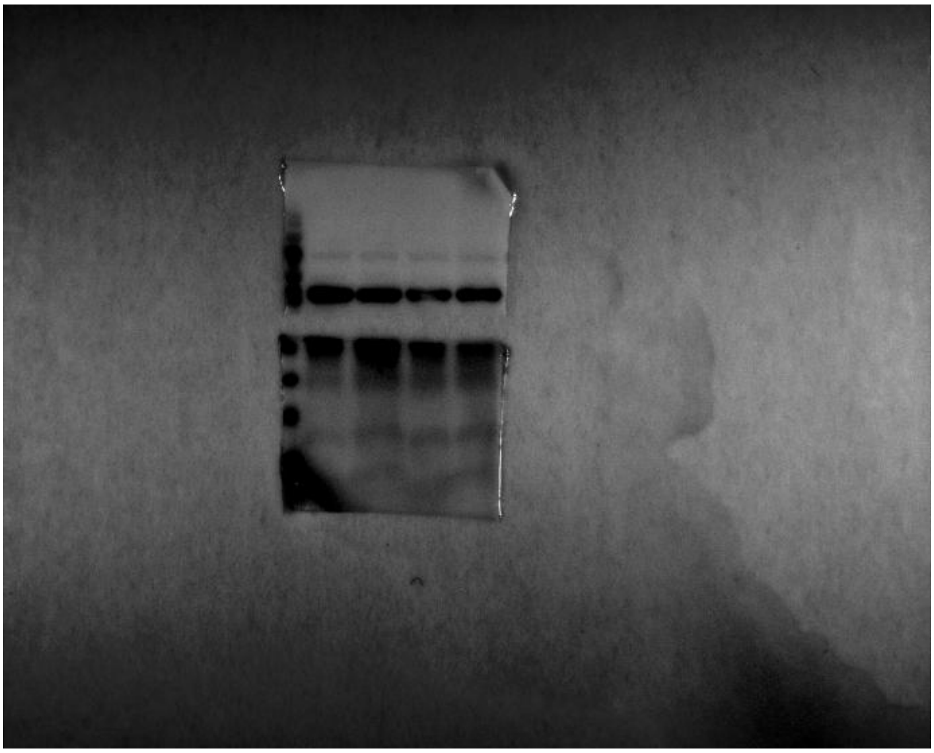

The second below blot

β - actin

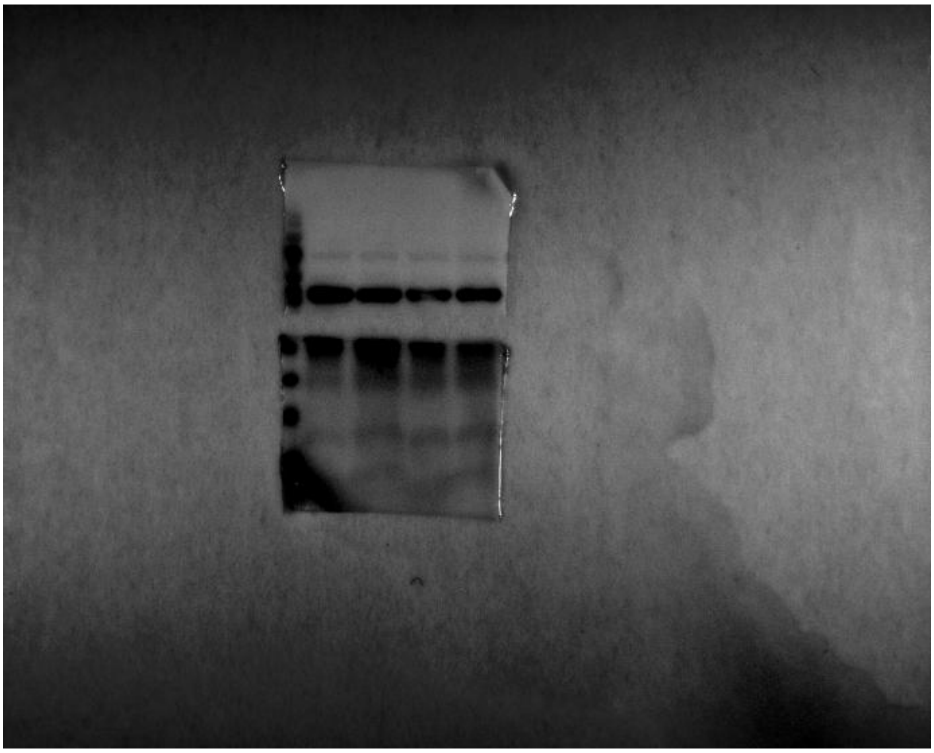

The middle blot

C-cas 9 - 1

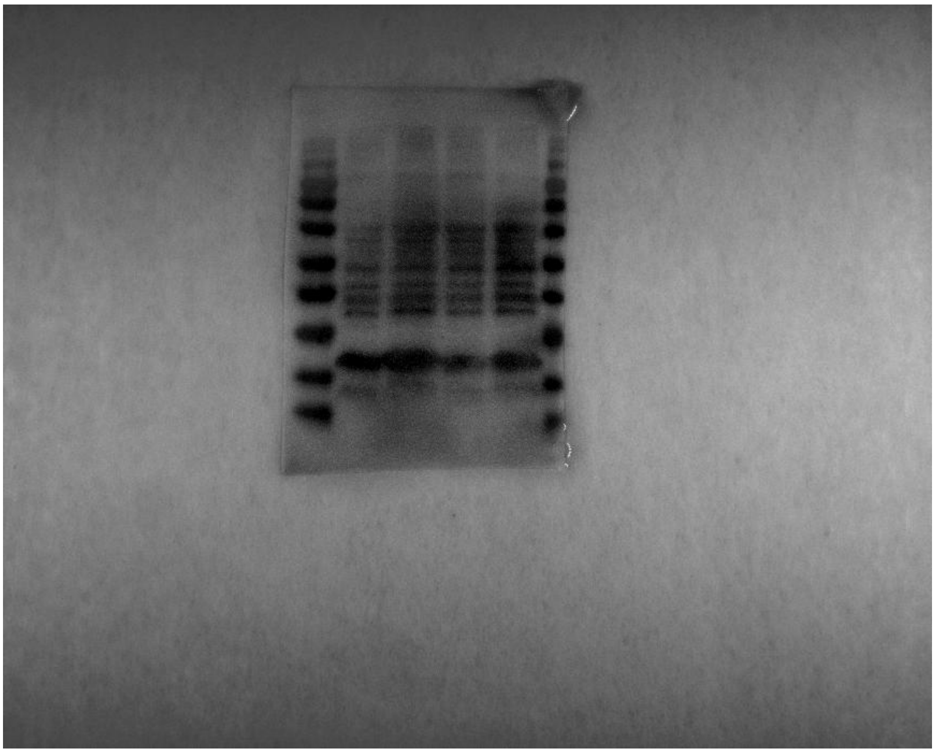

β - actin

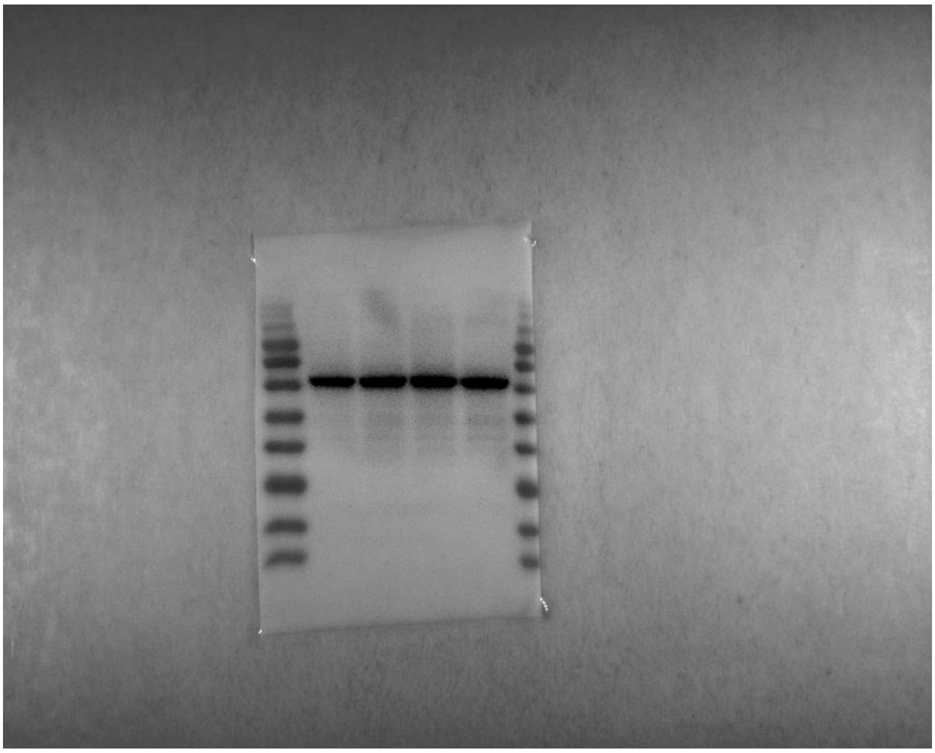

C-cas 9 - 2

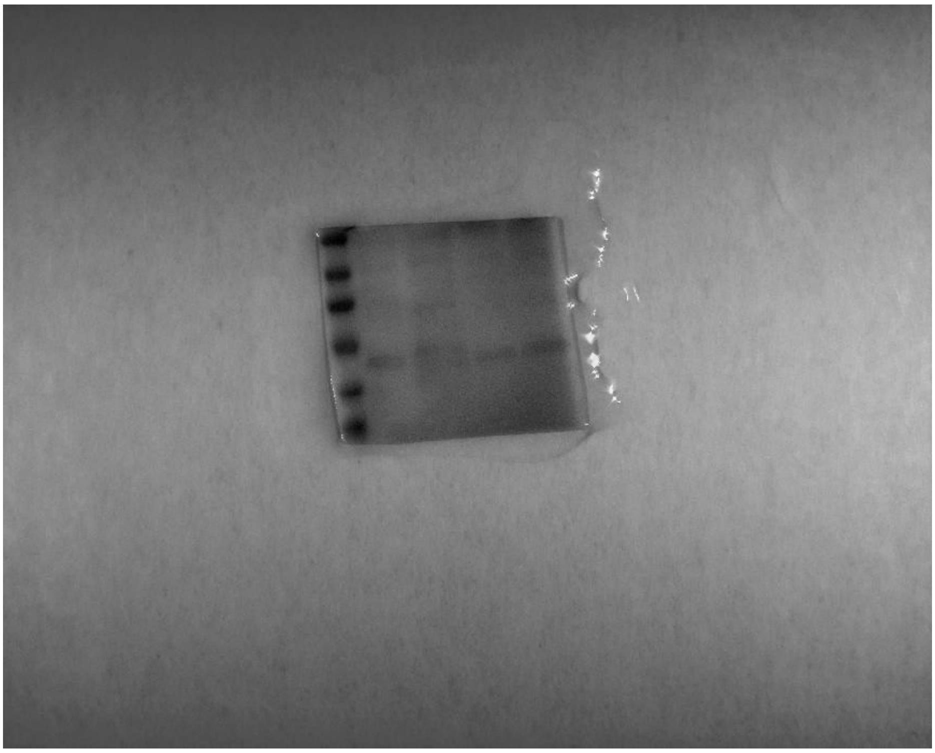

β - actin

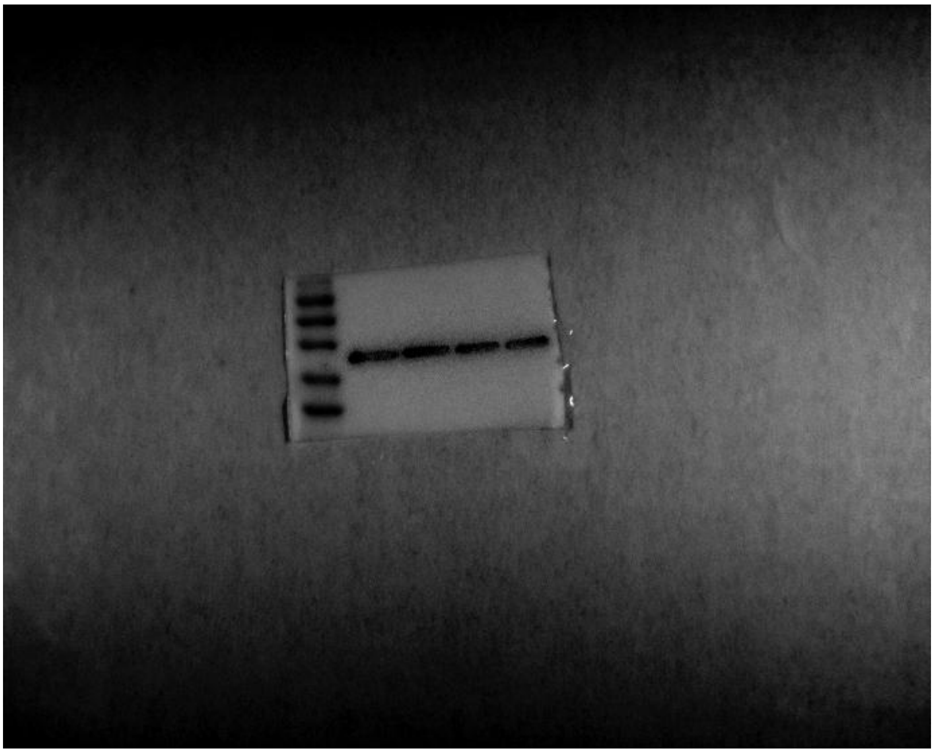

C-cas 9 - 3

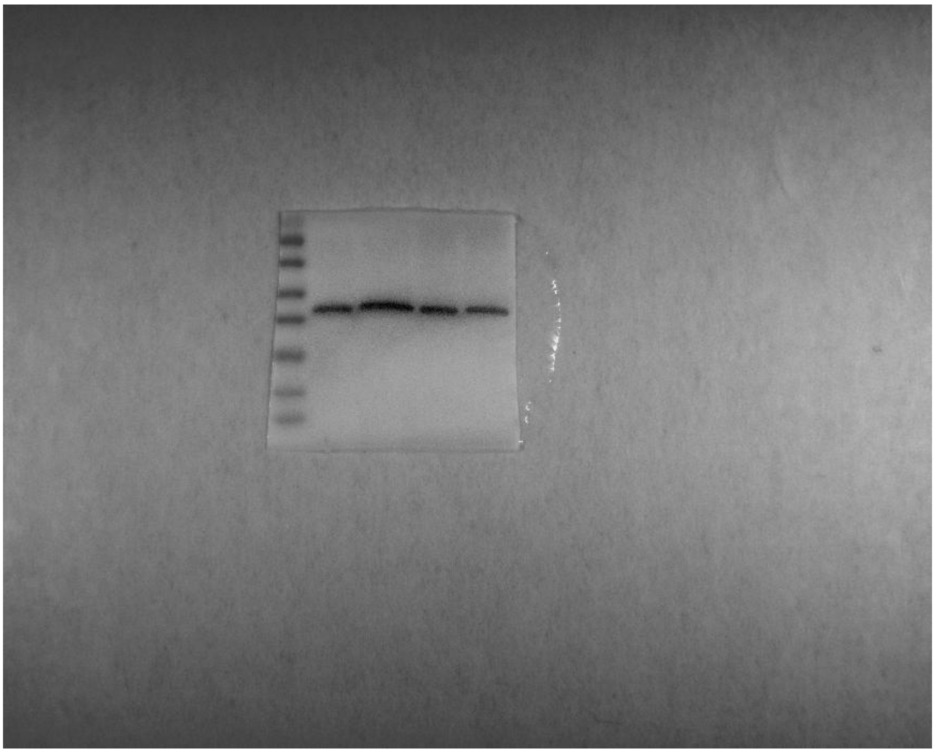

β - actin

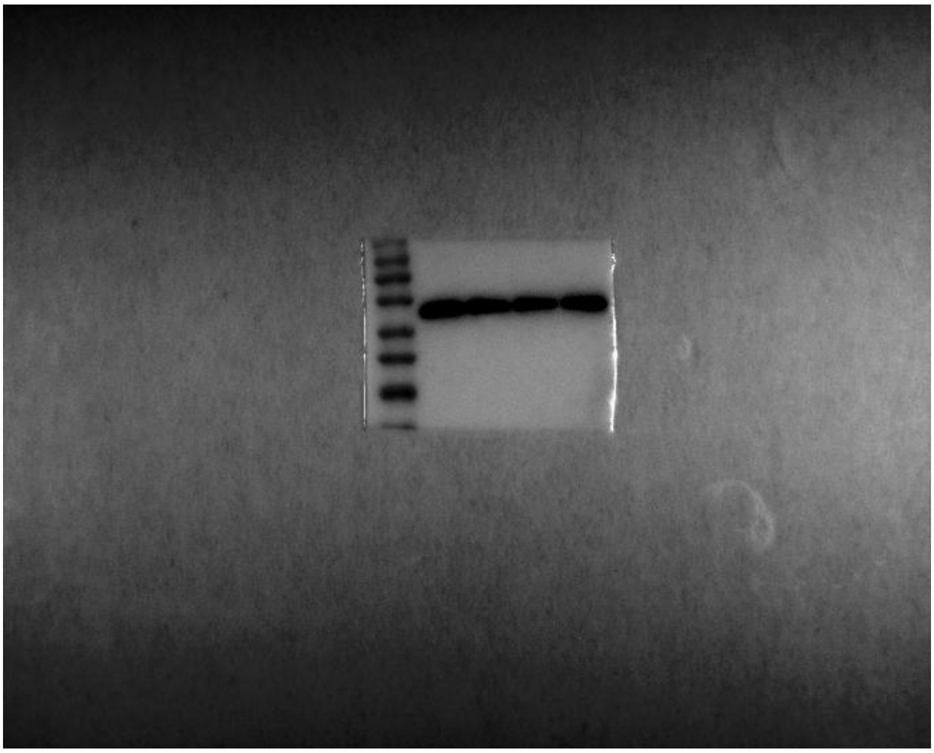

P53 - 1

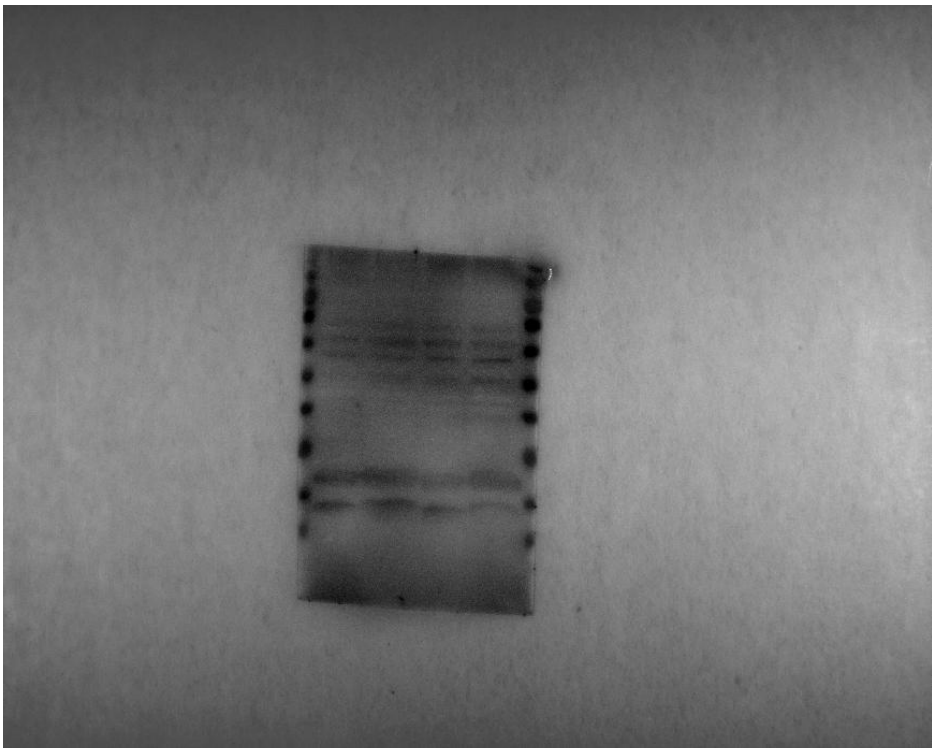

β - actin

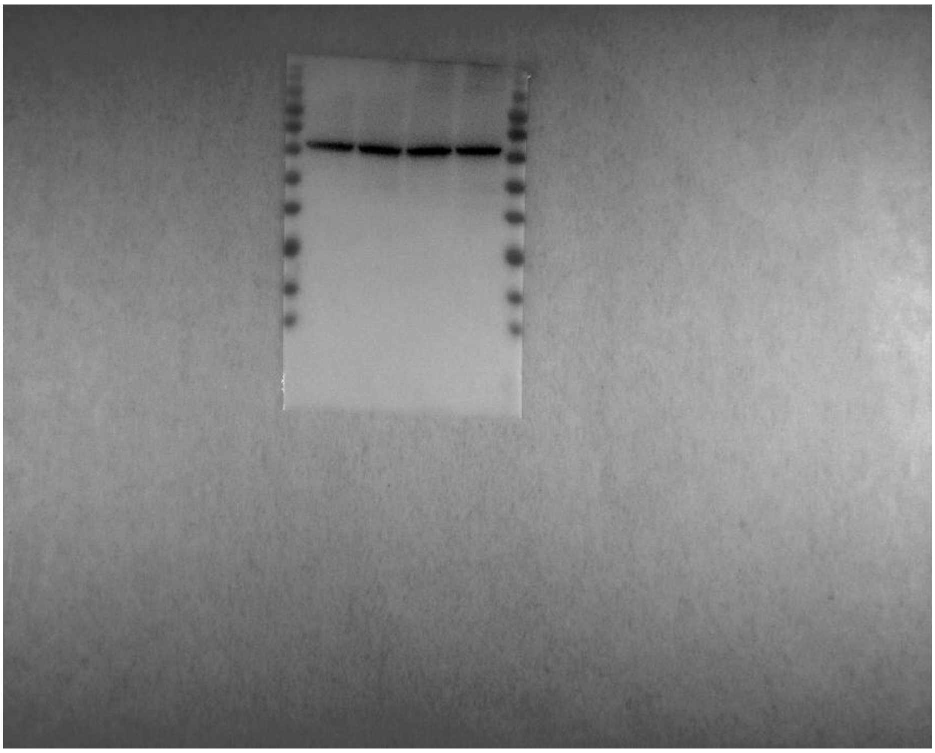

P53 - 2

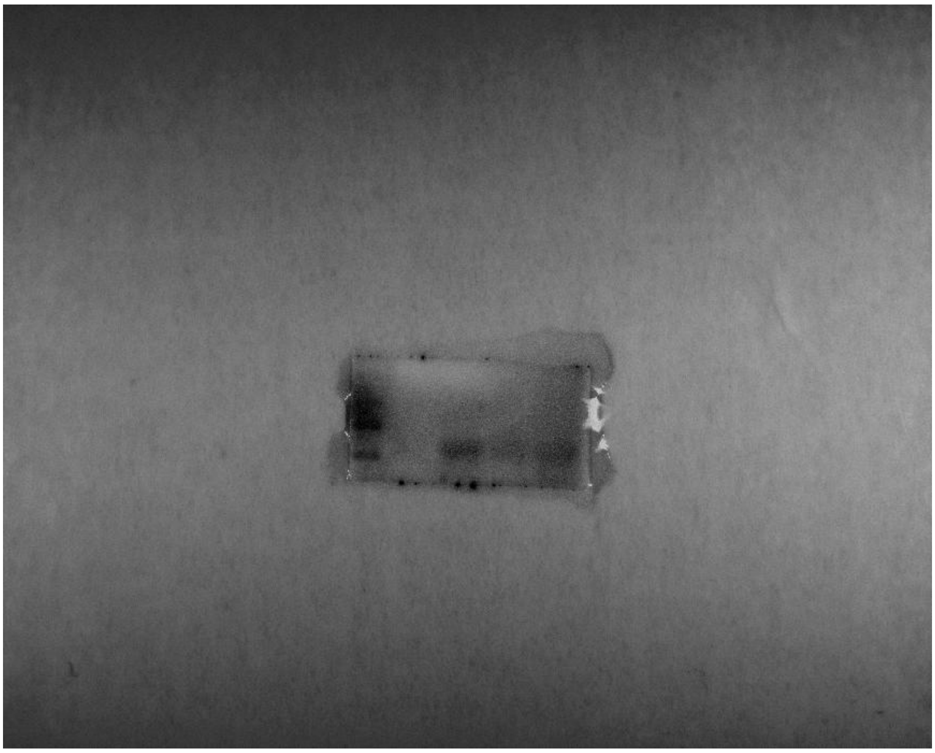

β - actin

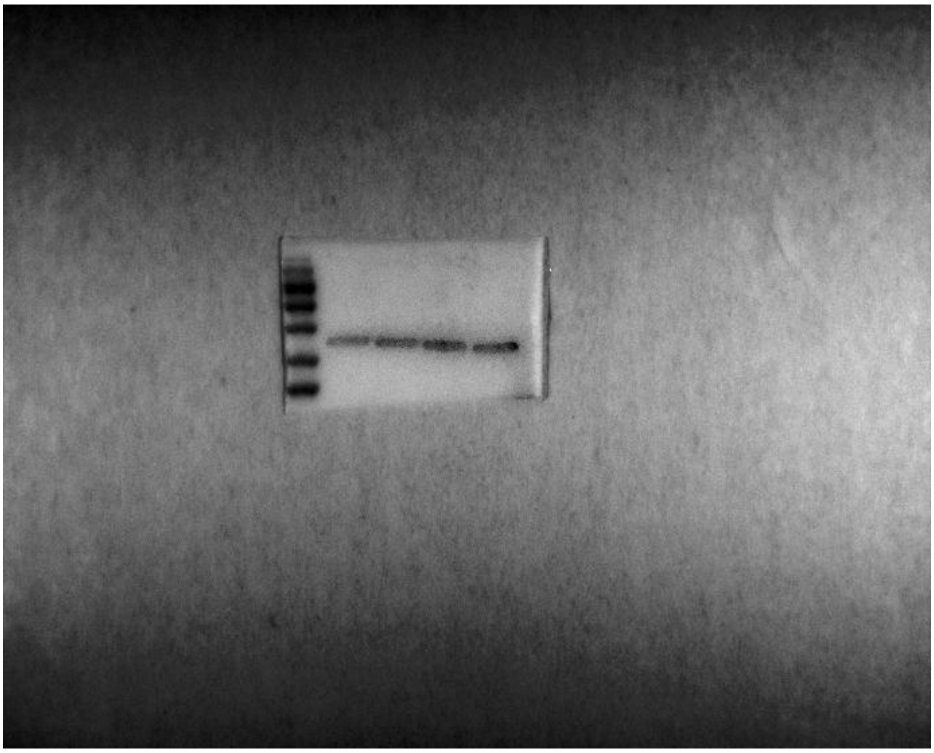

P53 - 3

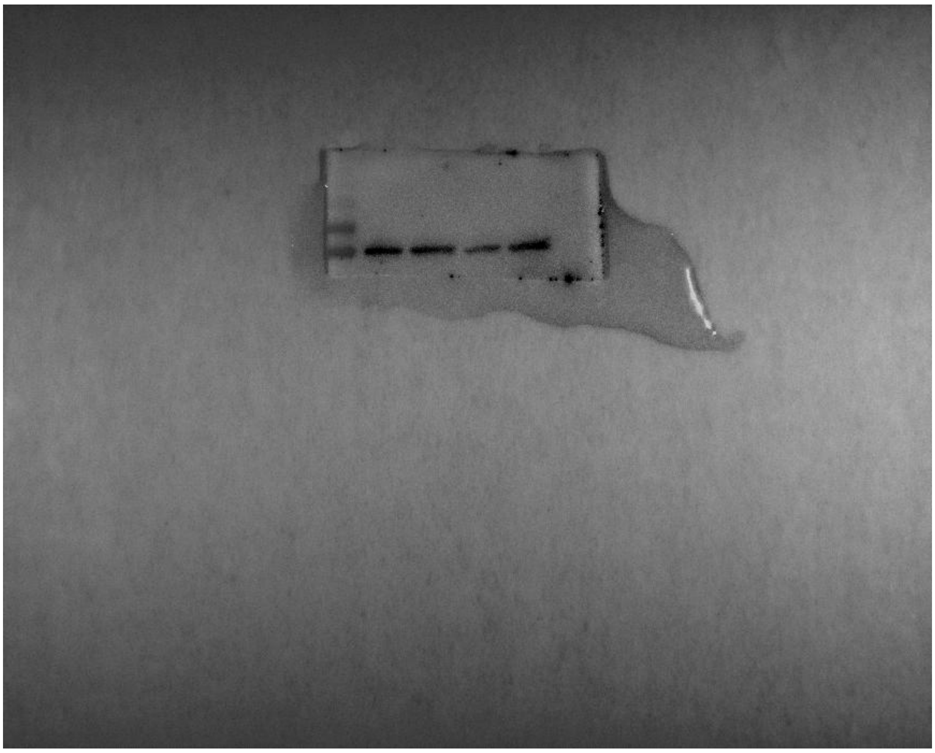

β - actin

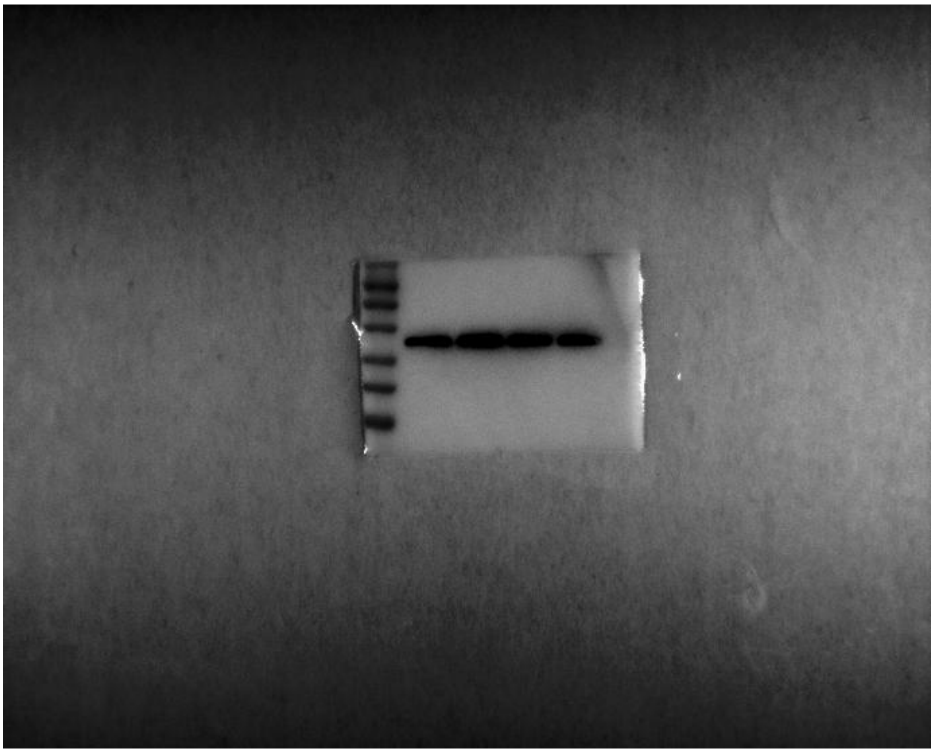

AKT - 1

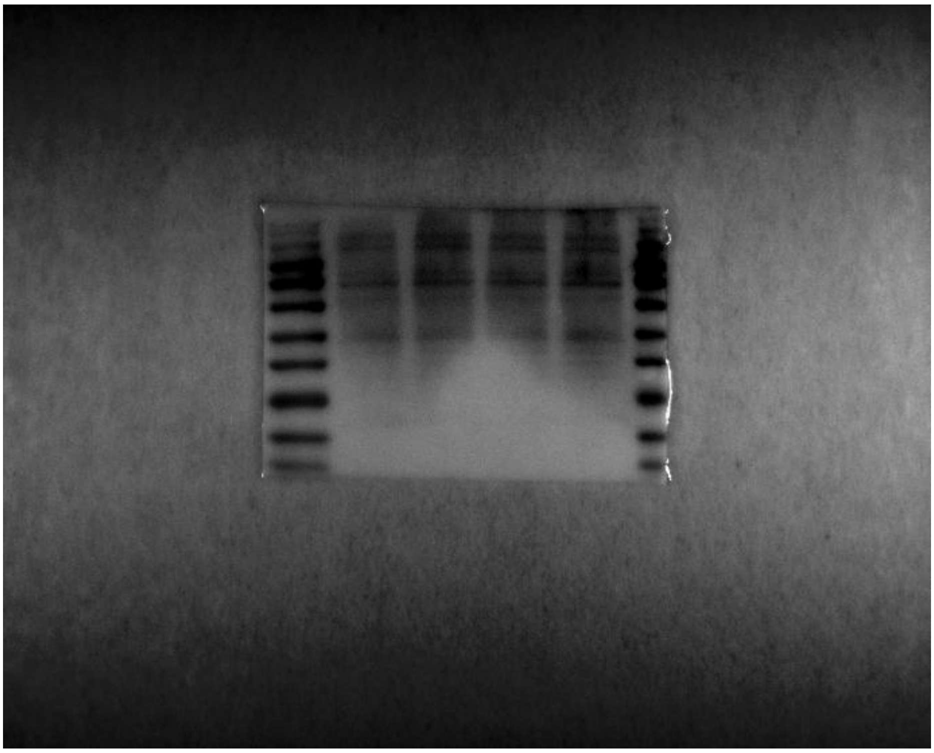

β - actin

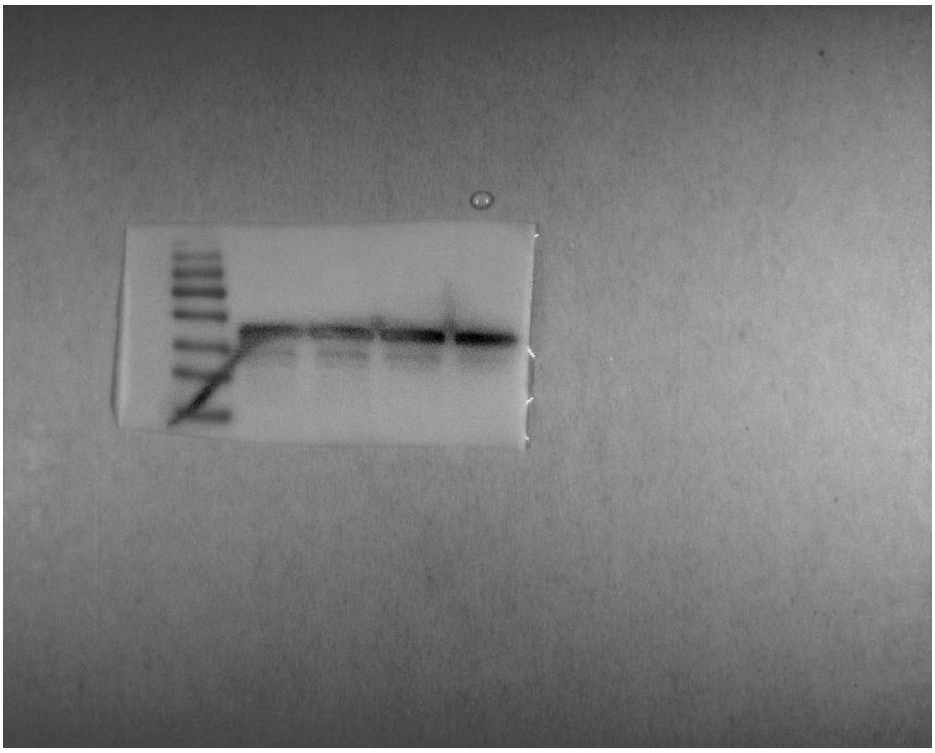

AKT - 2

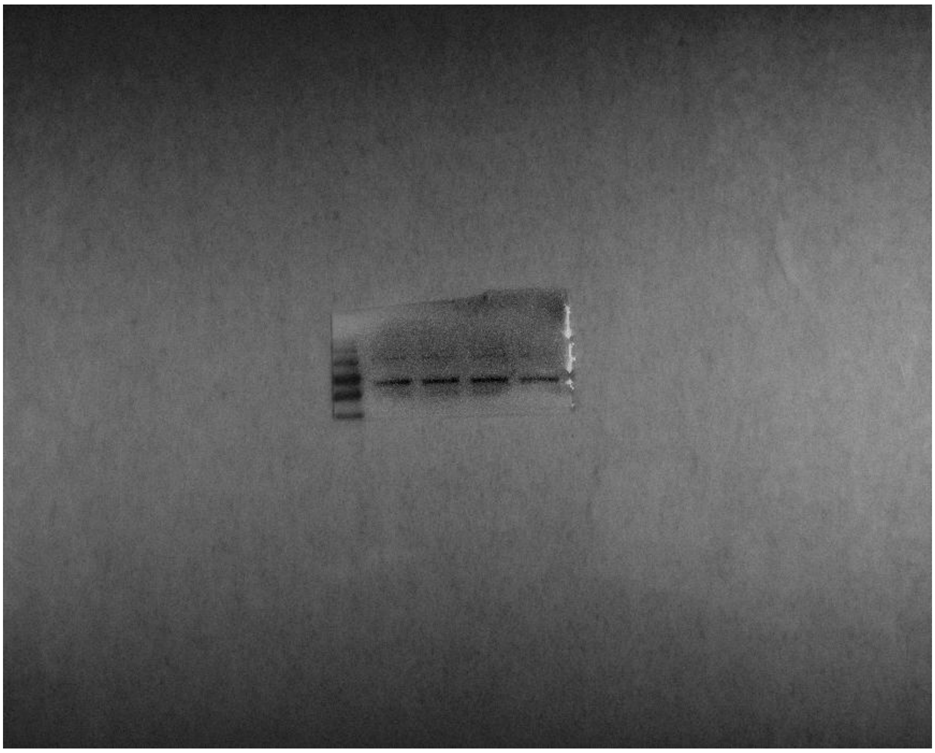

β - actin

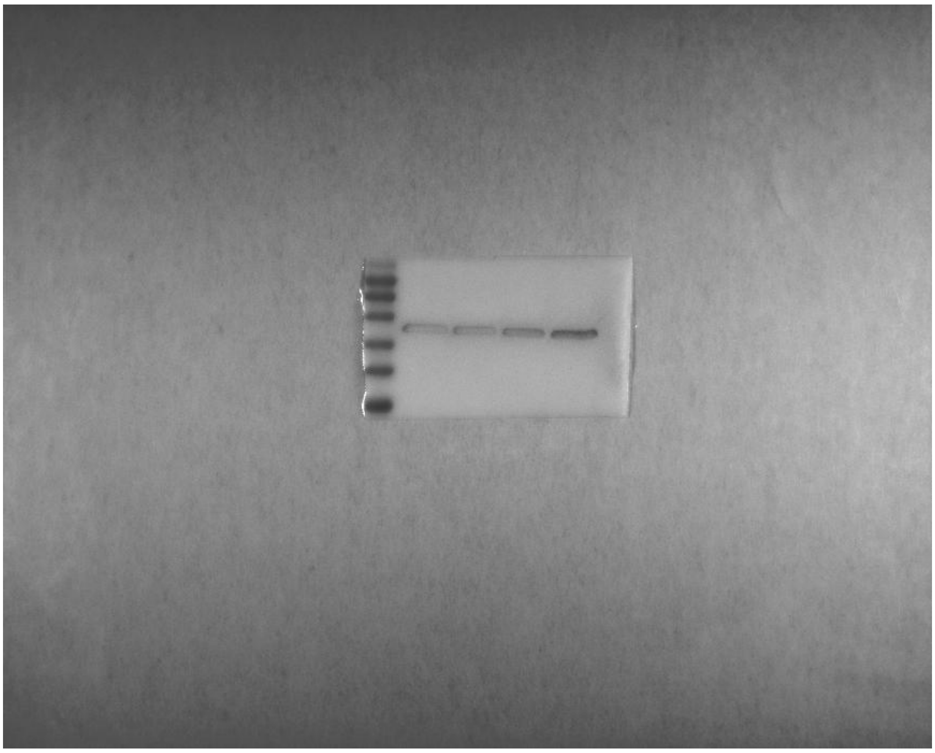

AKT - 3

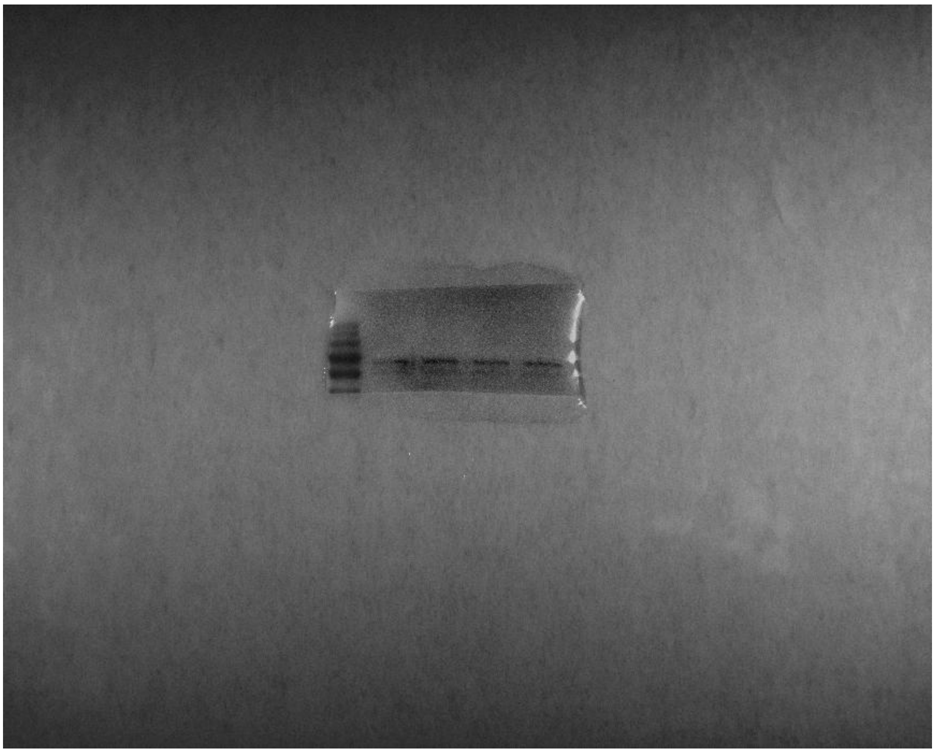

β - actin

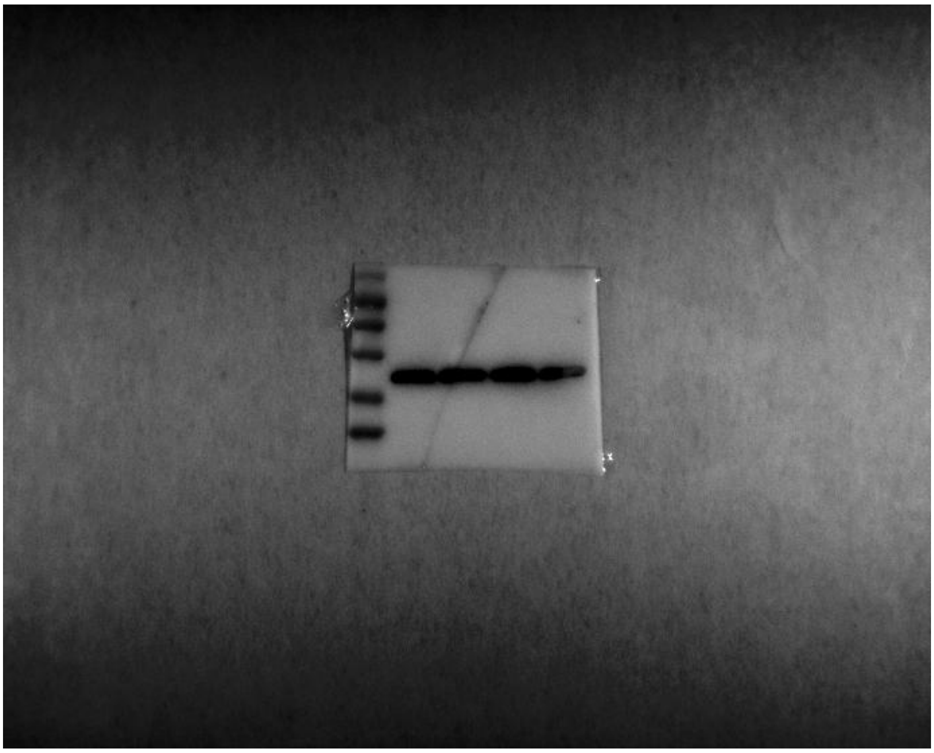

P-AKT - 1

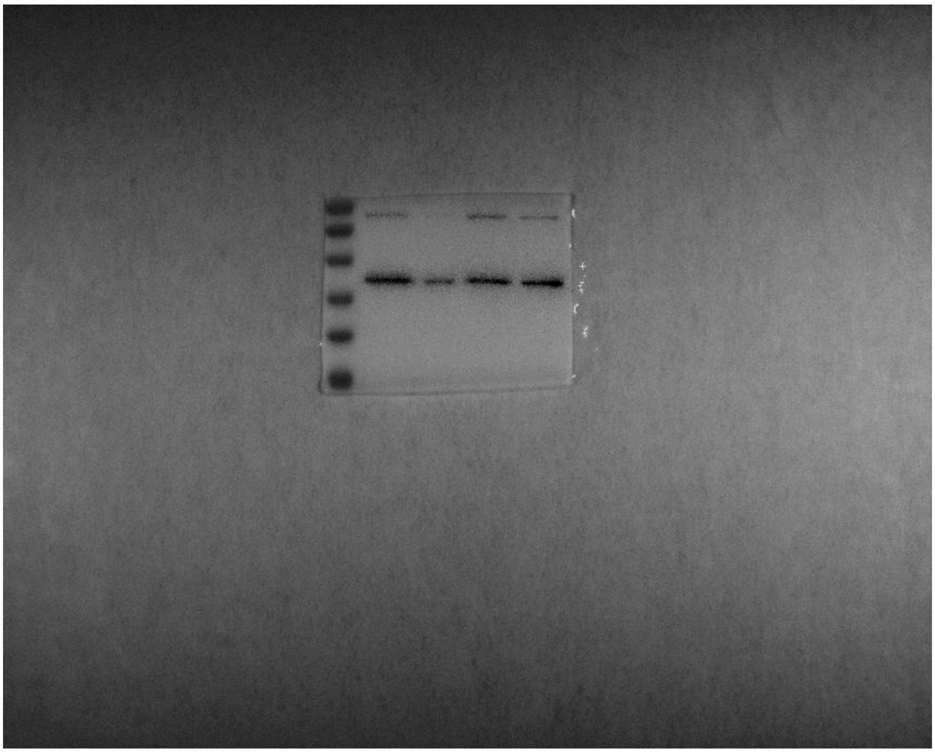

β - actin

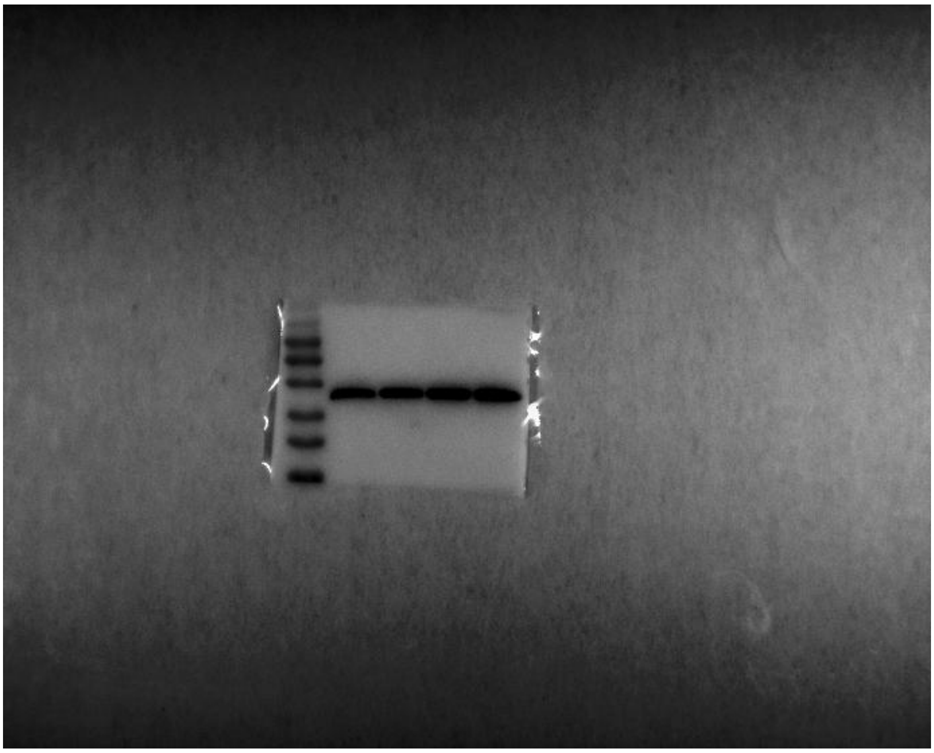

P-AKT - 2

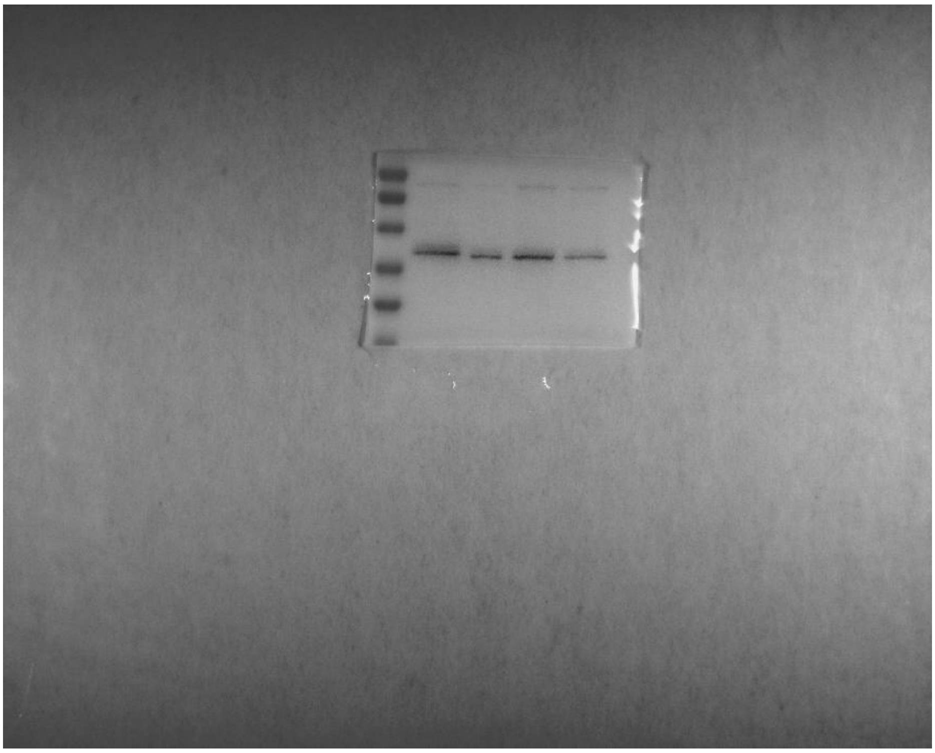

β - actin

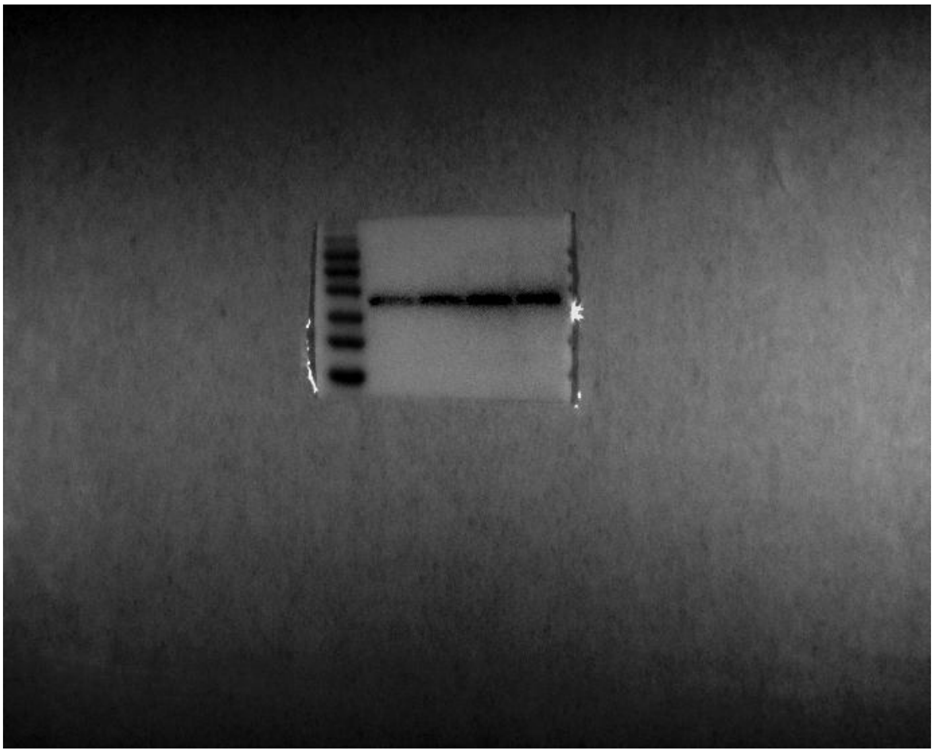

P-AKT - 3

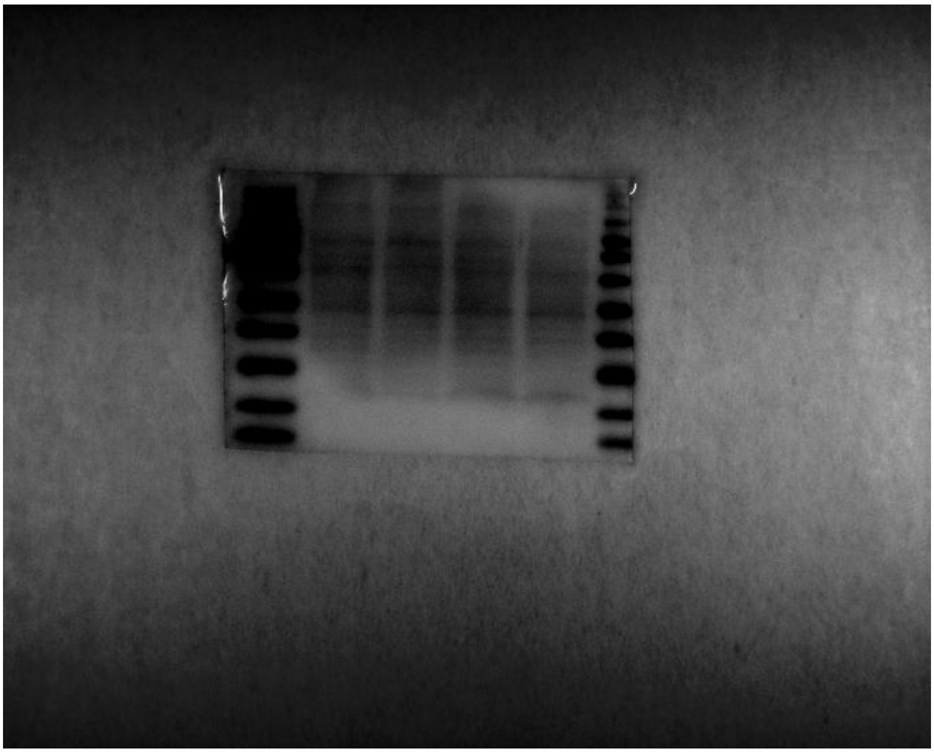

β - actin

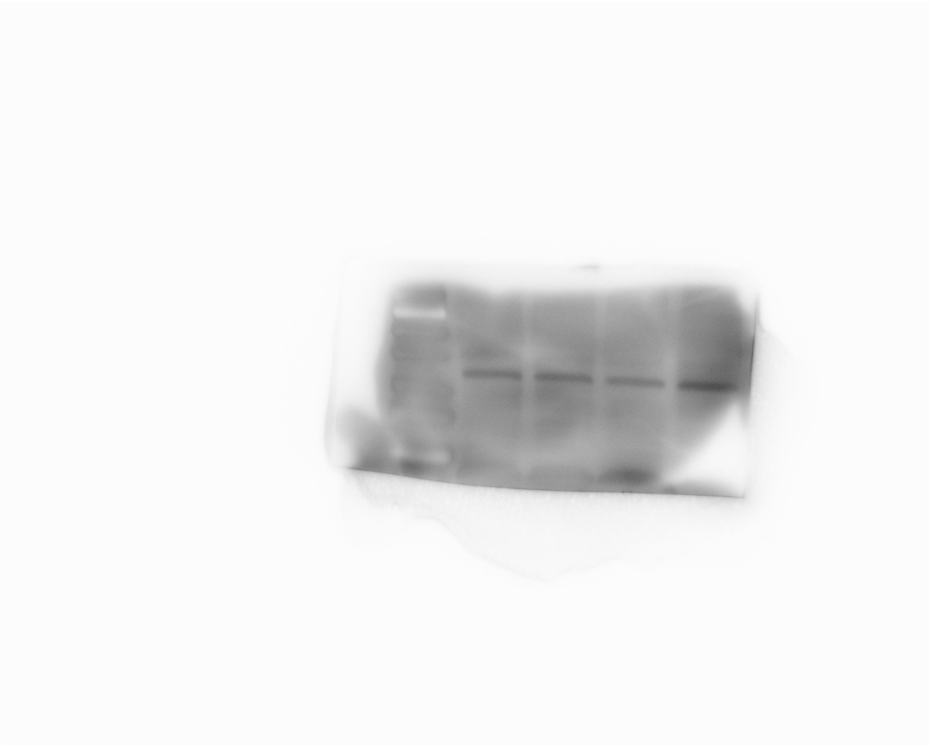

Western blot in cell

From left to right is CTR DOX DOX + shHDAC2 shHDAC2
CTR : control
DOX : Doxorubicin

HDAC2 - 1

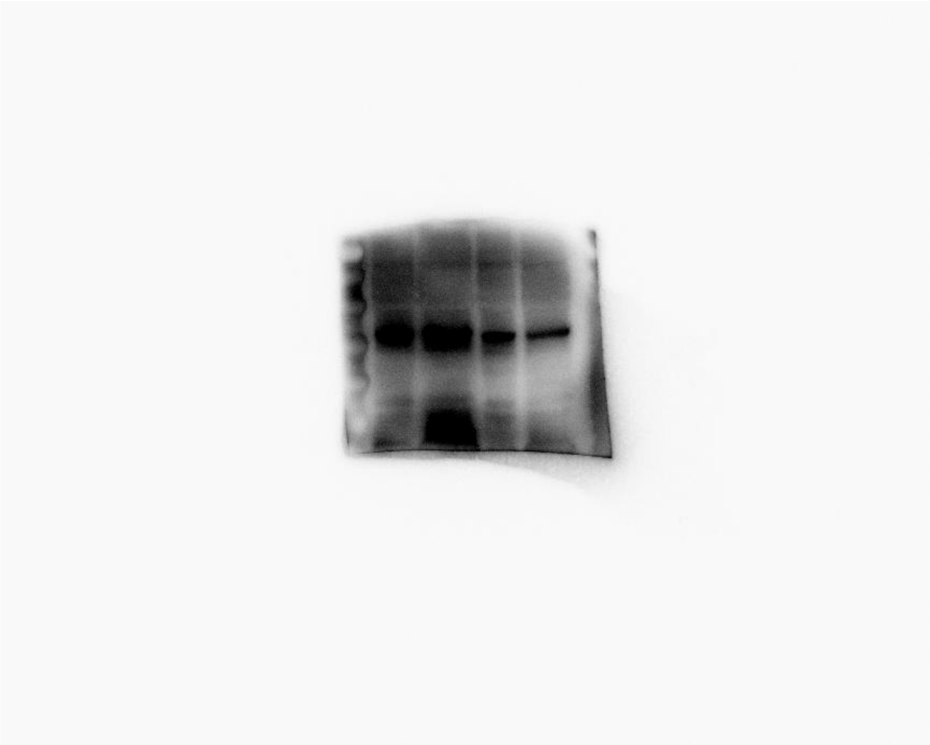

β -actin

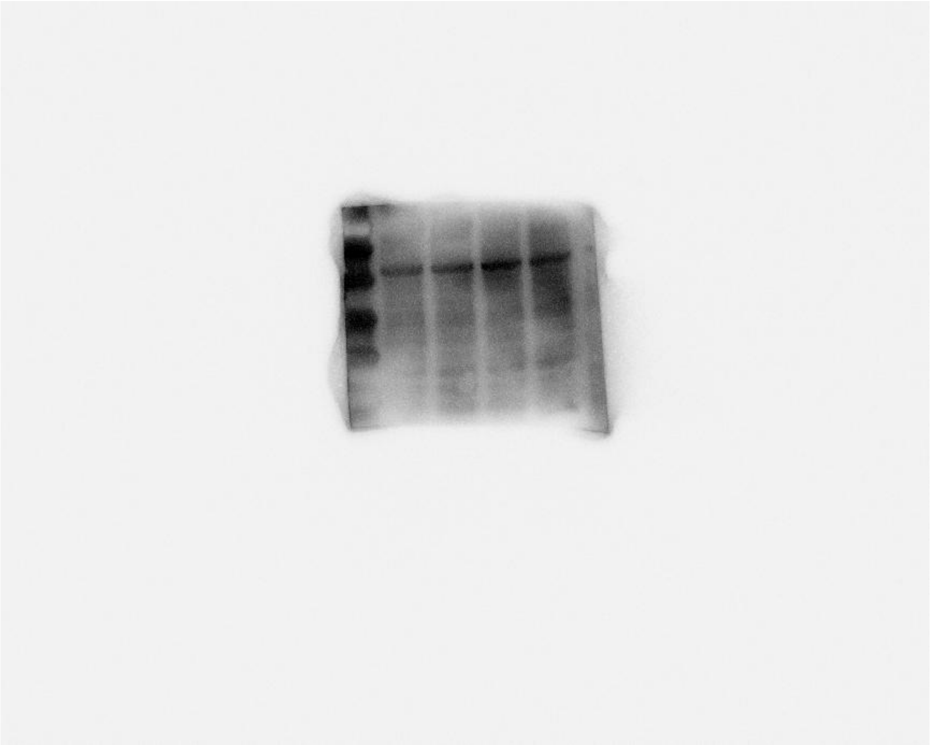

HDAC2 - 2

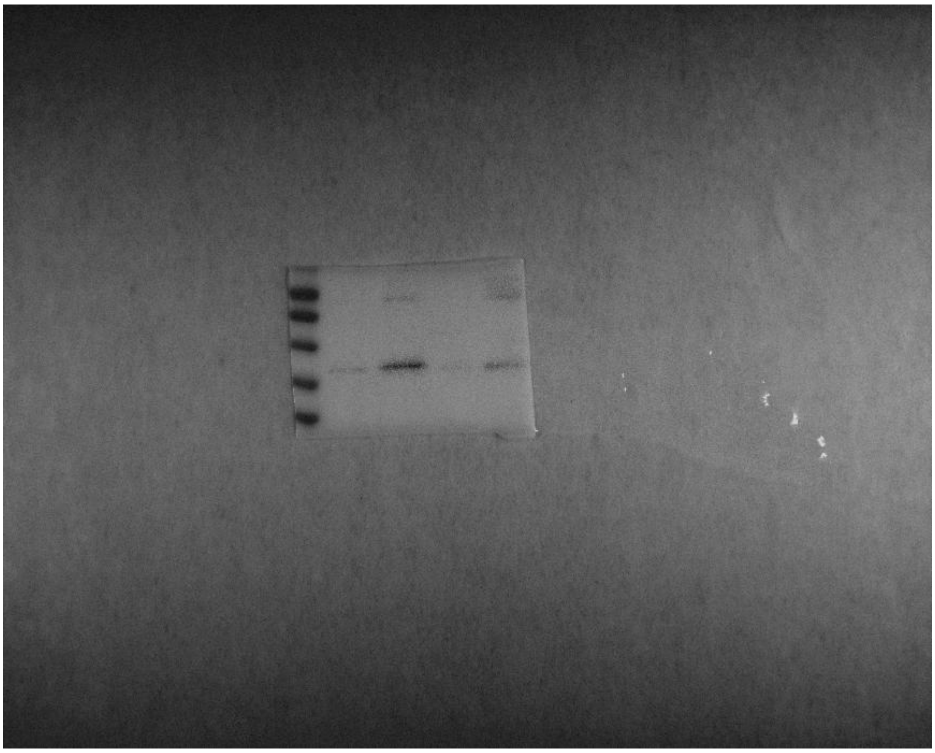

β - actin

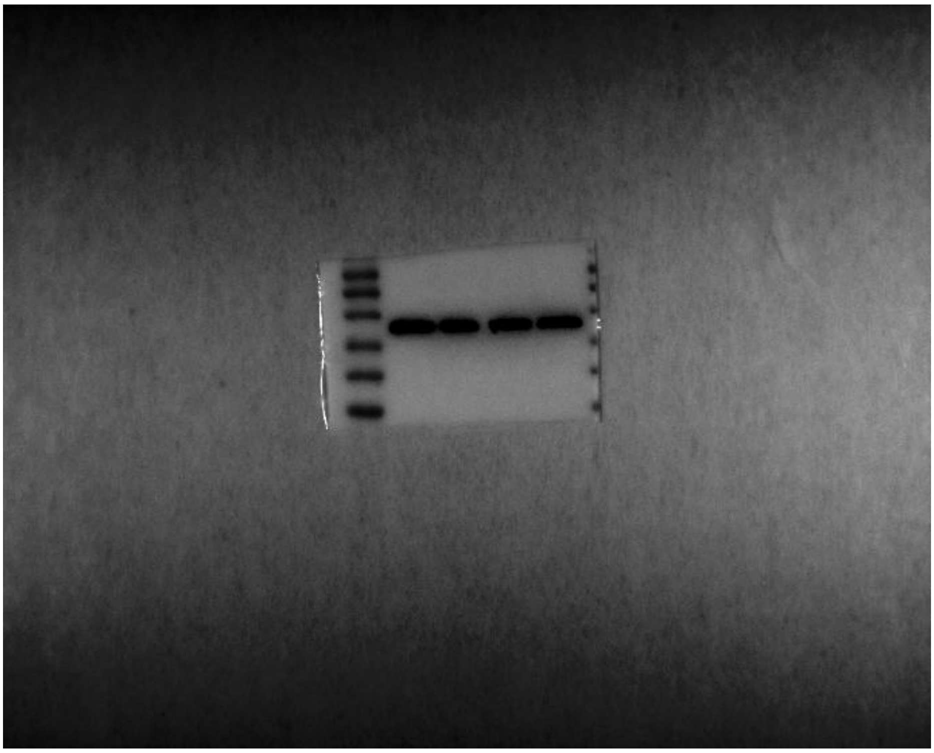

HDAC2 - 3

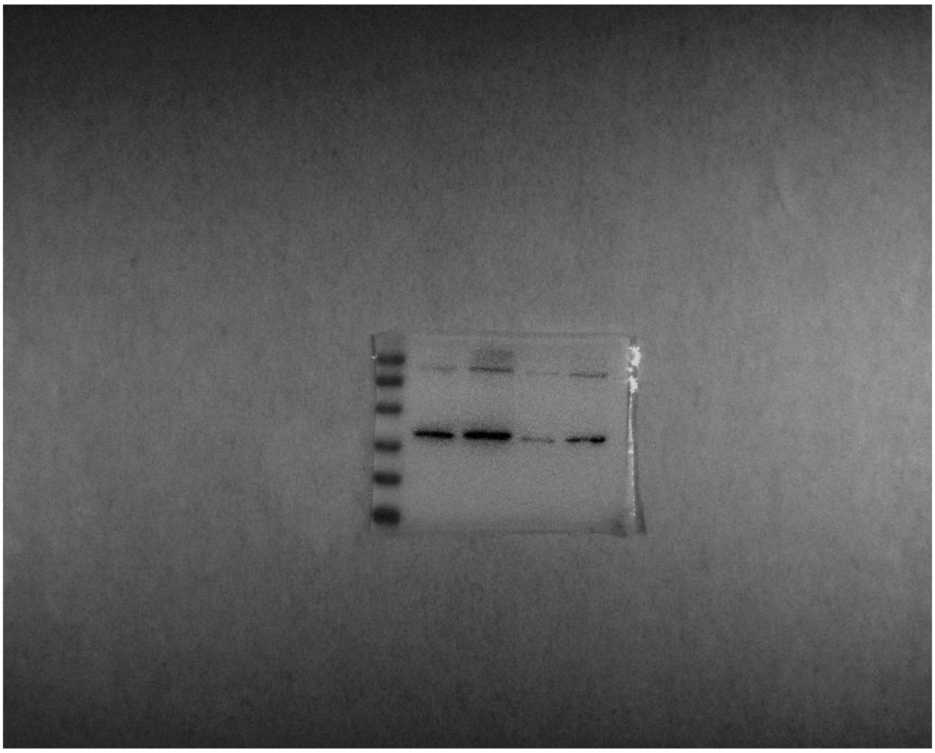

β - actin

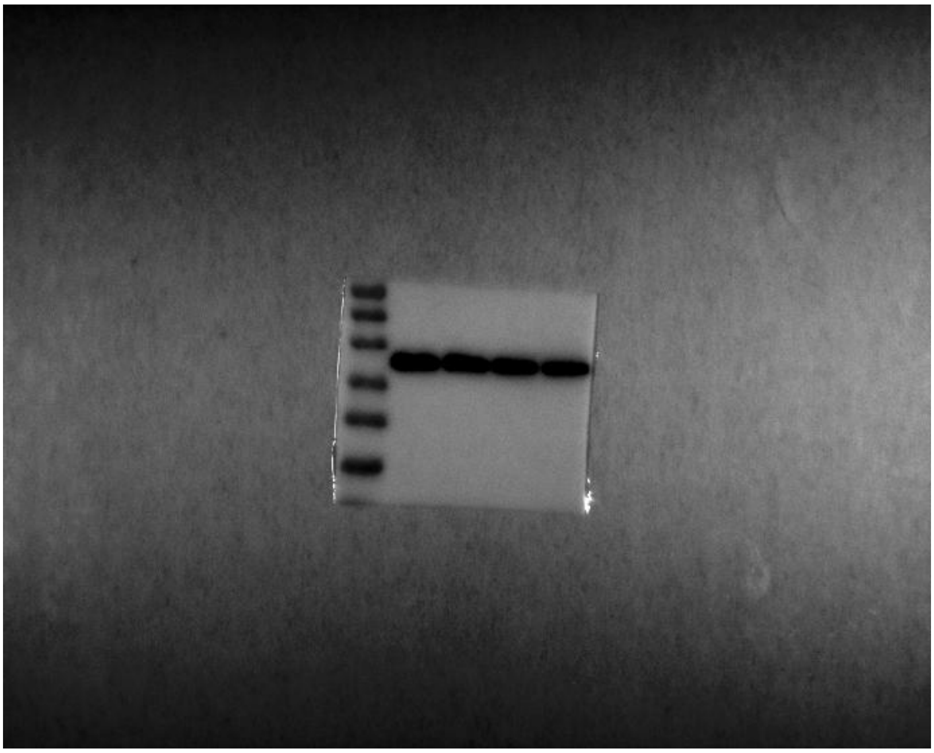

BAX - 1

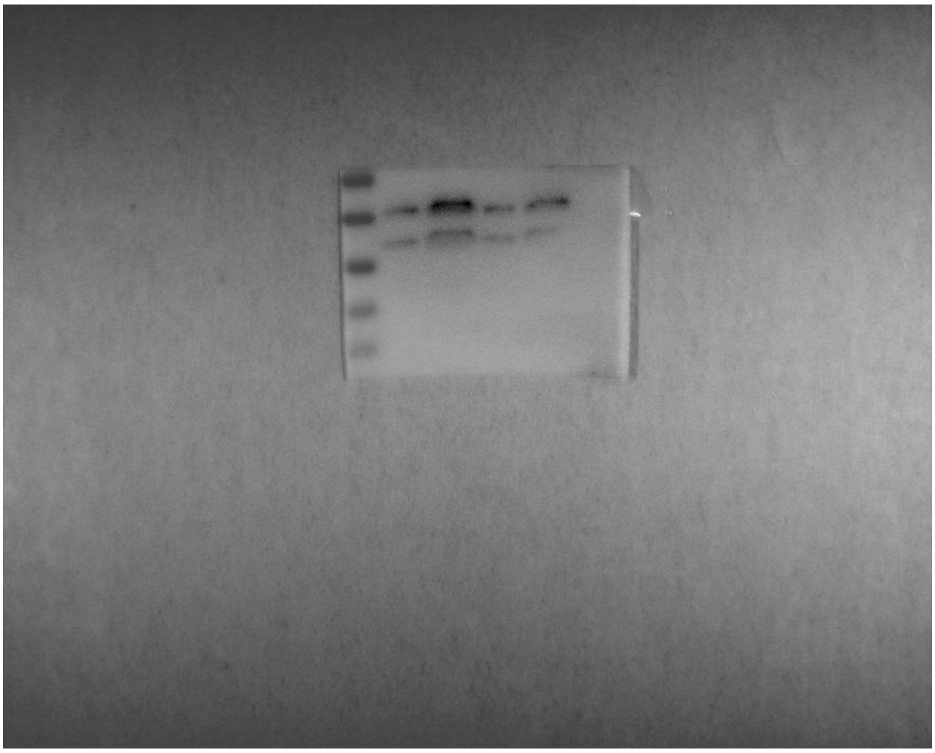

β - actin

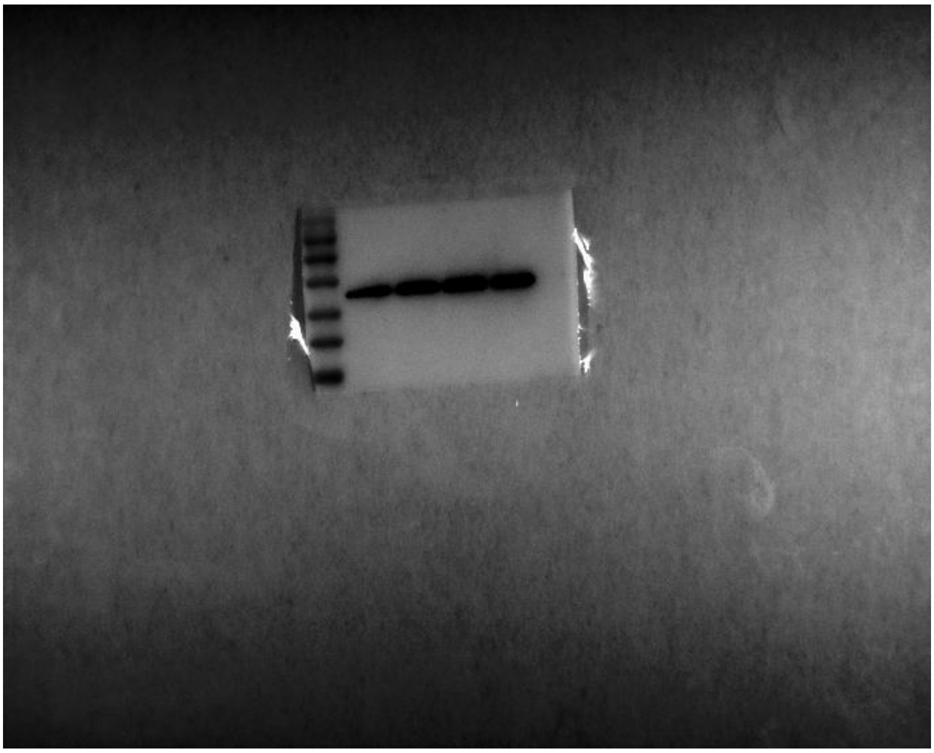

BAX - 2

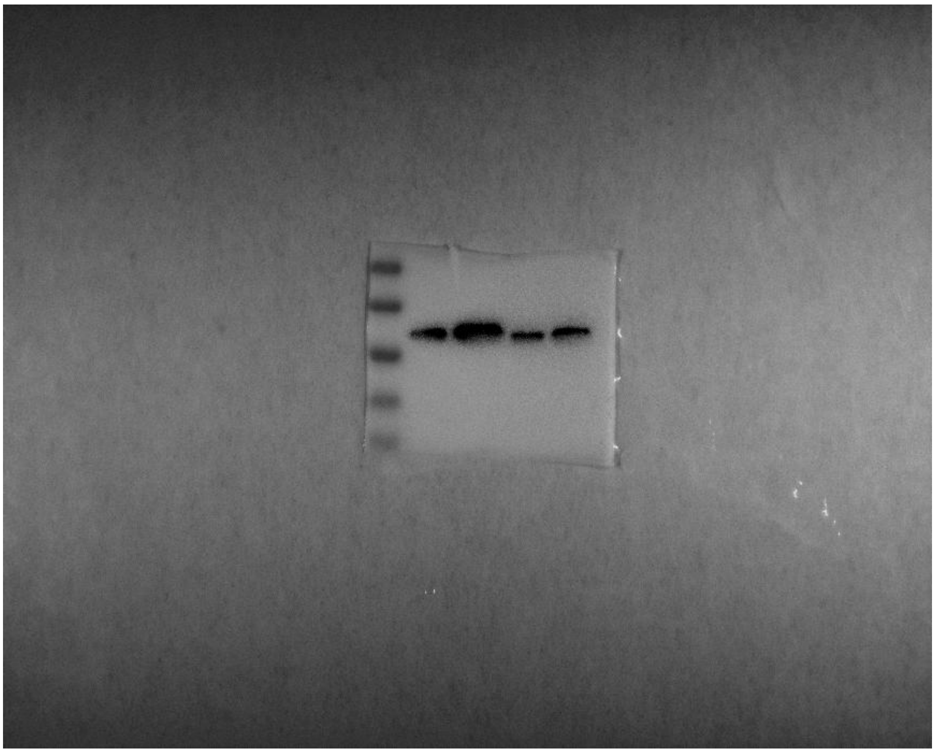

β - actin

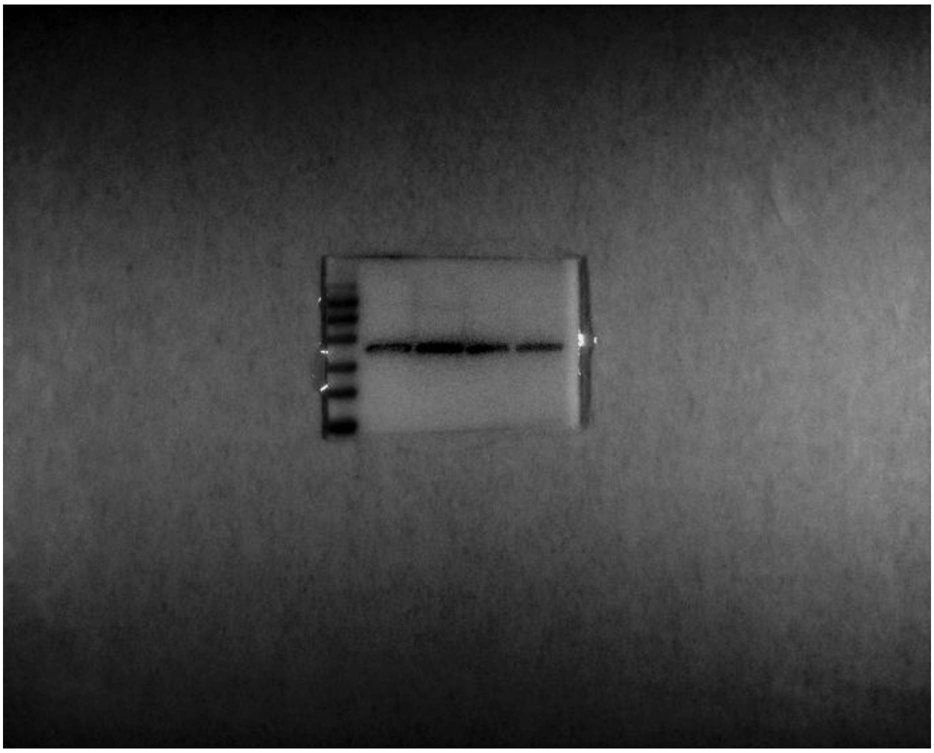

BAX - 3

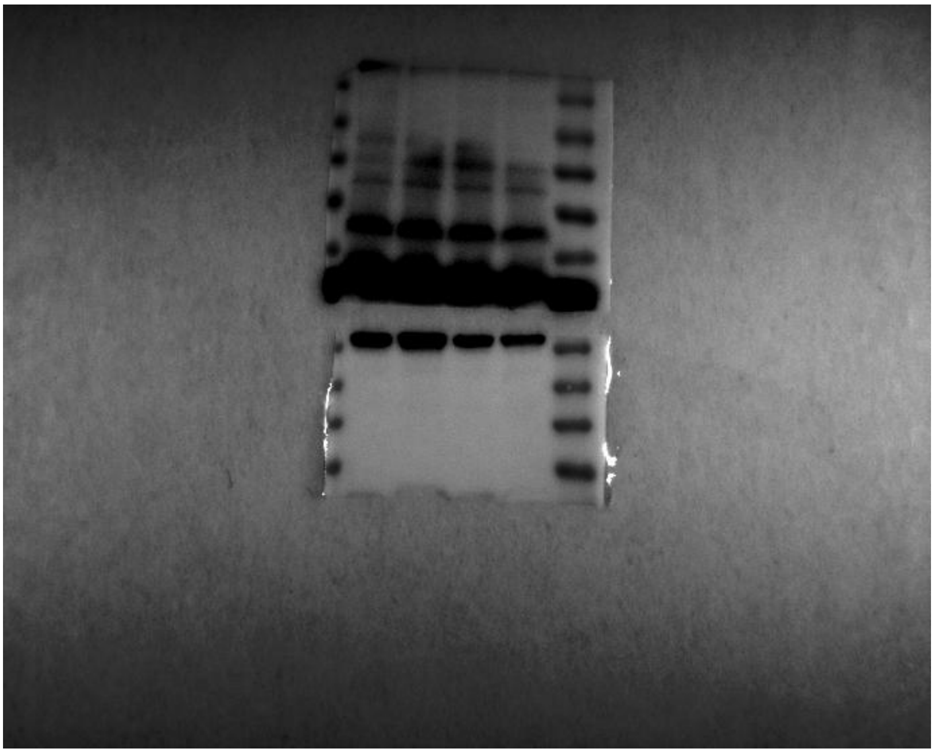

The below blot

β - actin

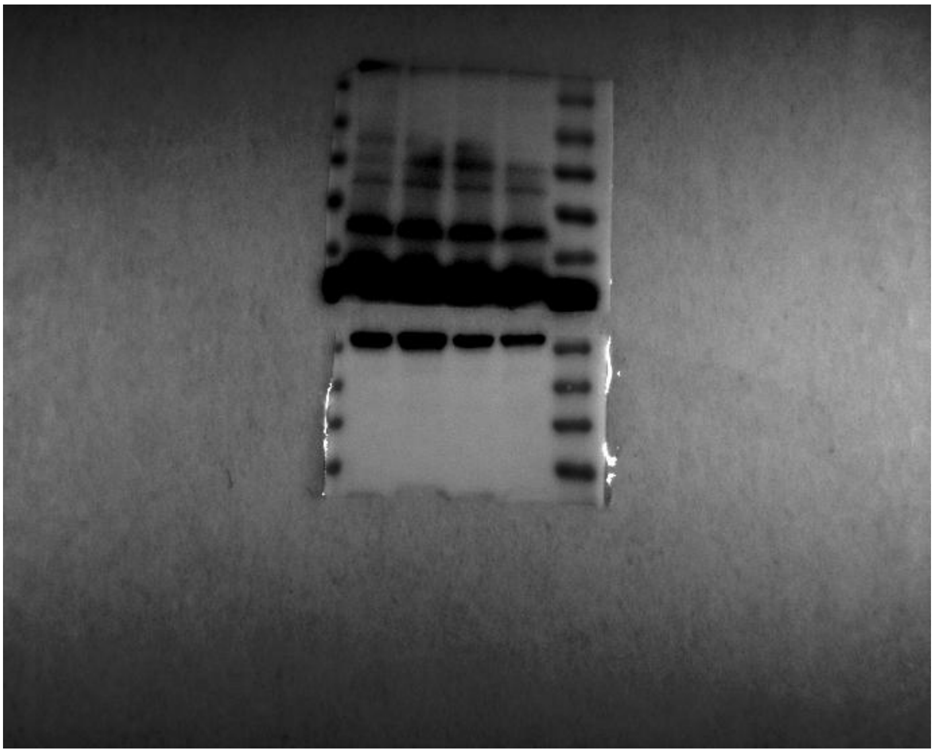

The third blot from top

Bcl-2 - 1

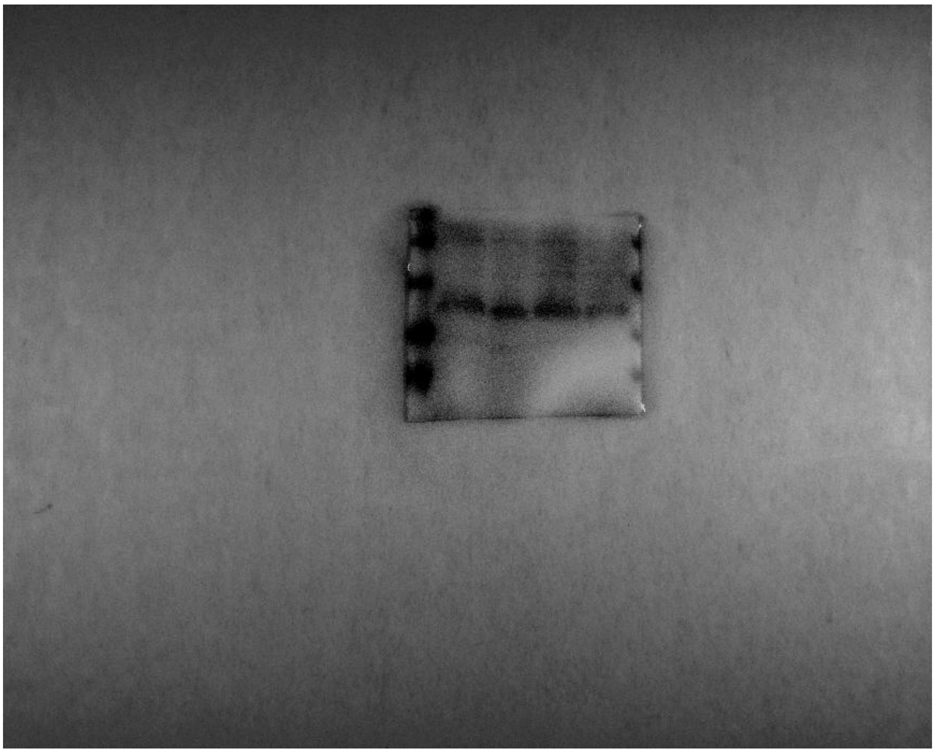

β - actin

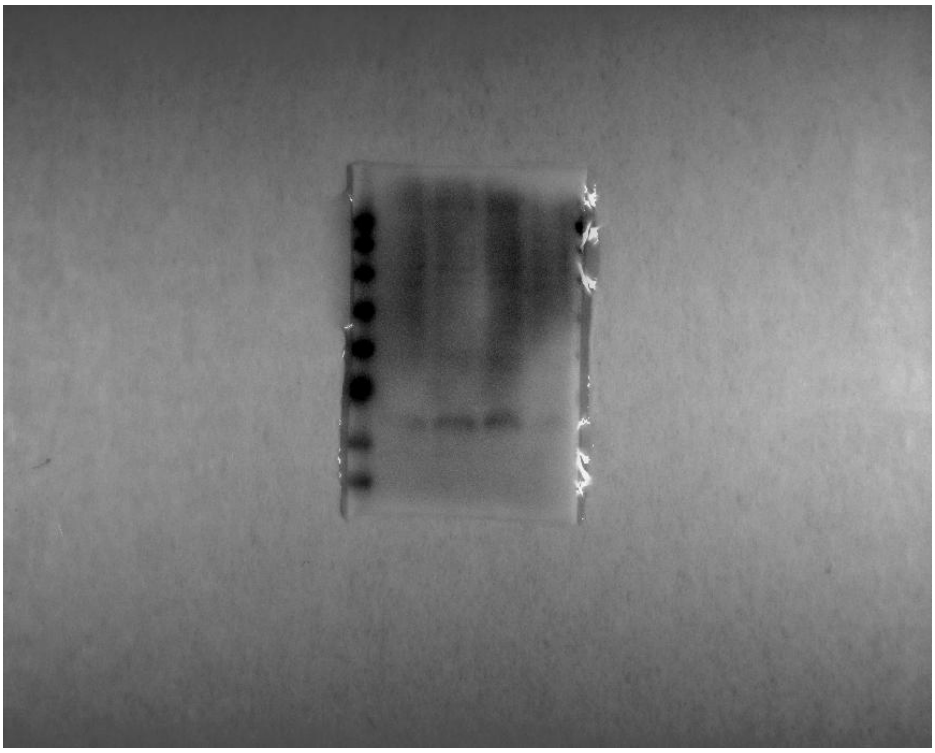

Bcl-2 - 2

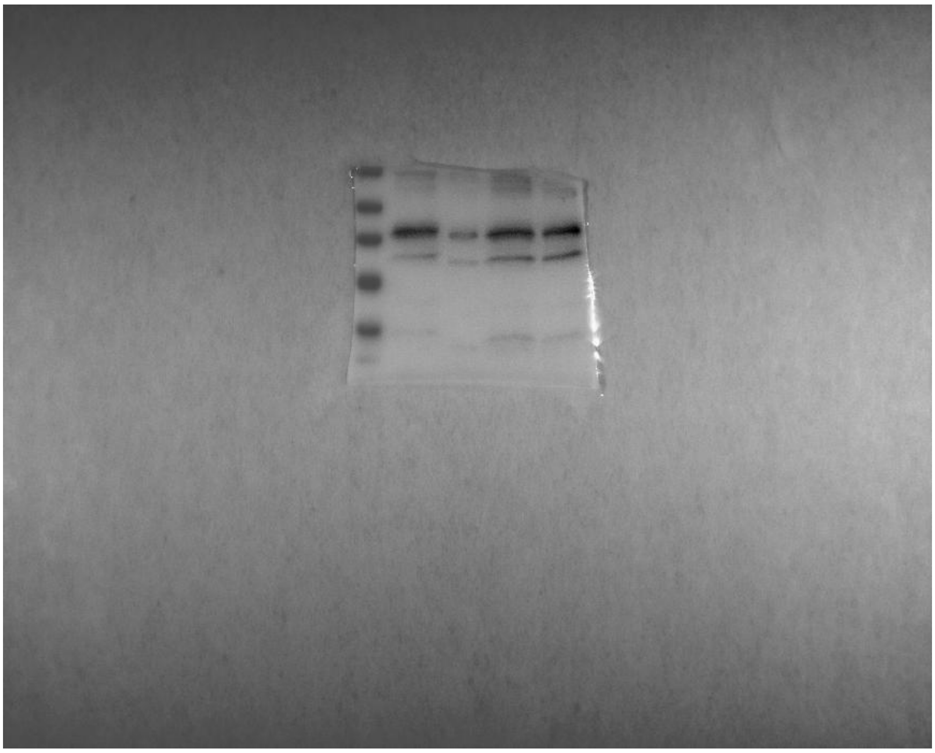

β - actin

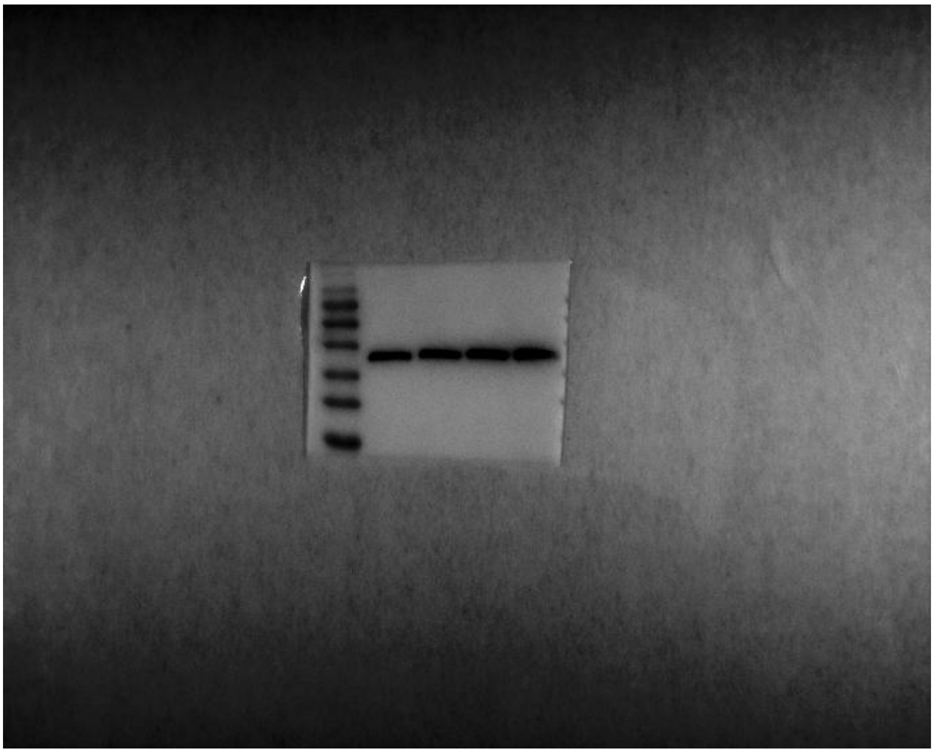

Bcl-2 - 3

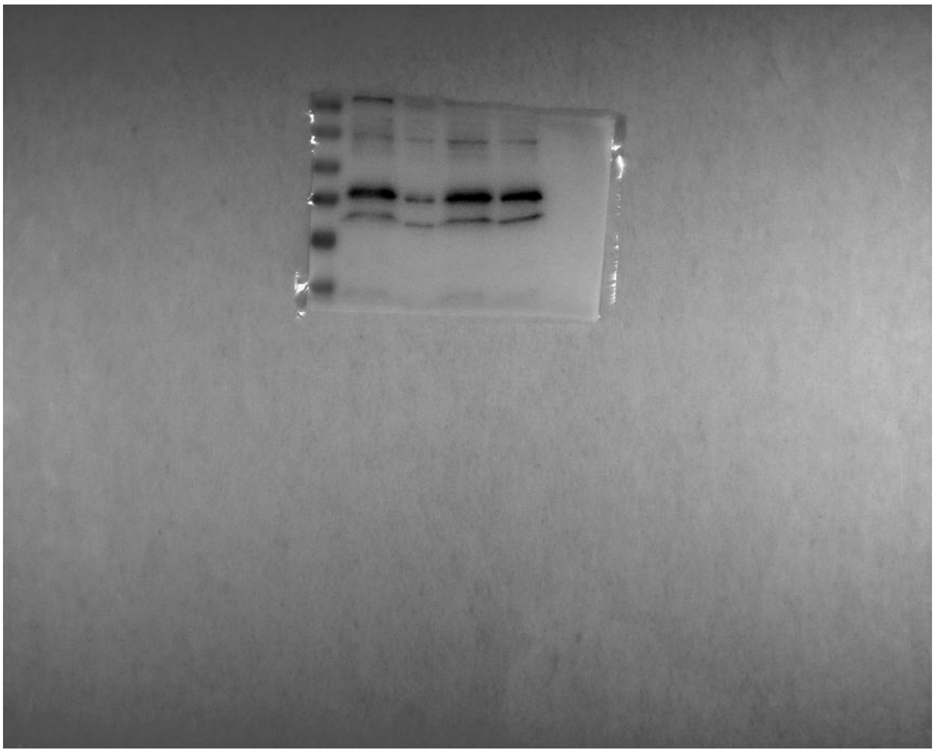

β - actin

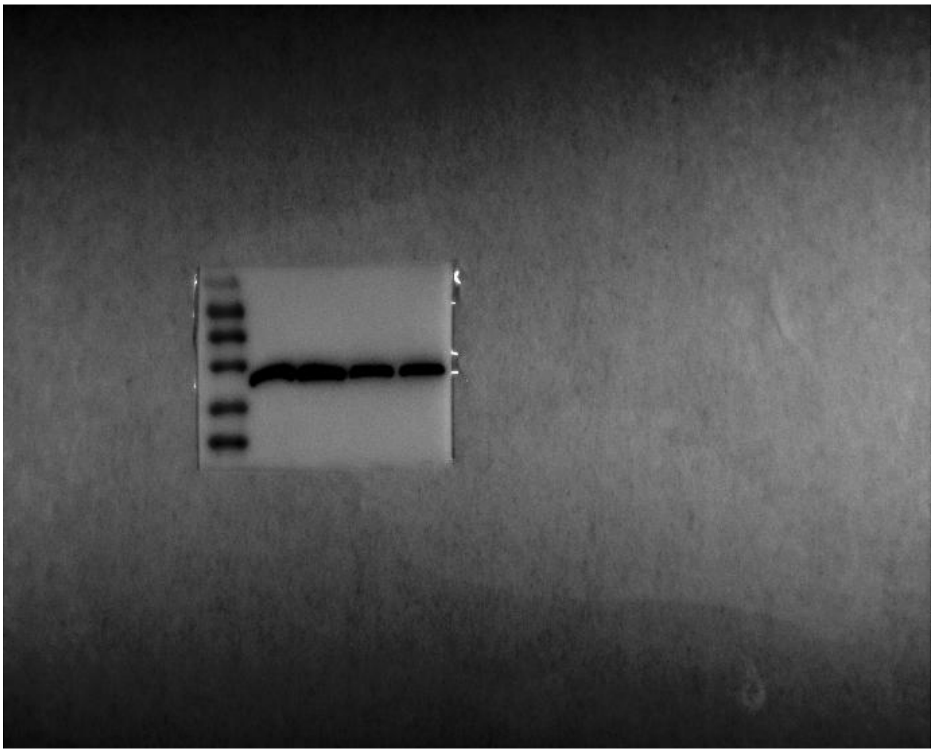

C-cas 3 - 1

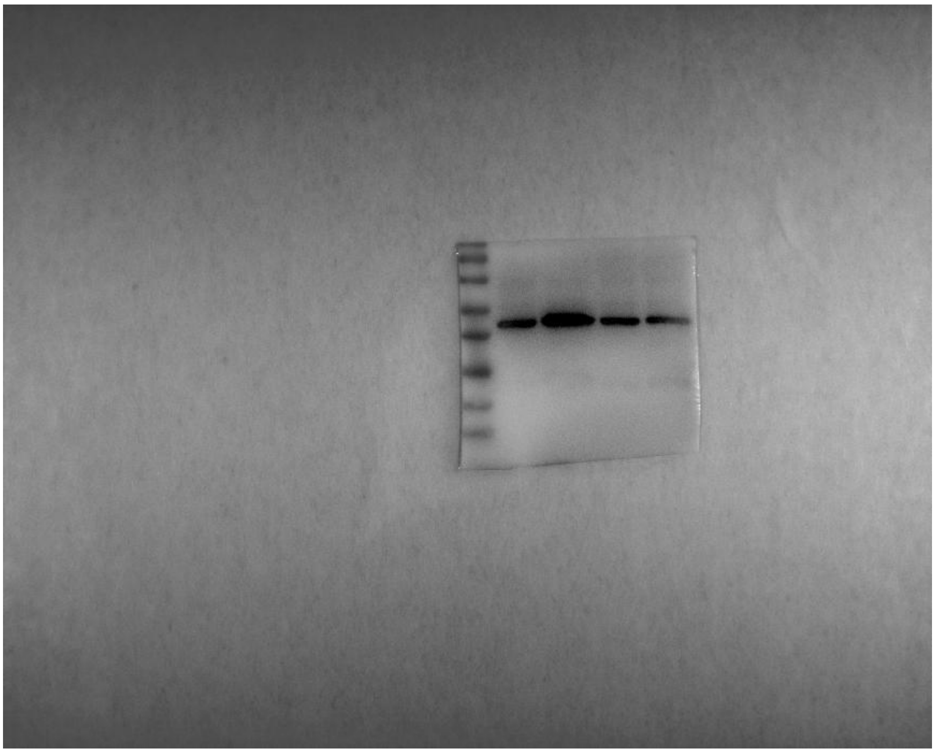

β - actin

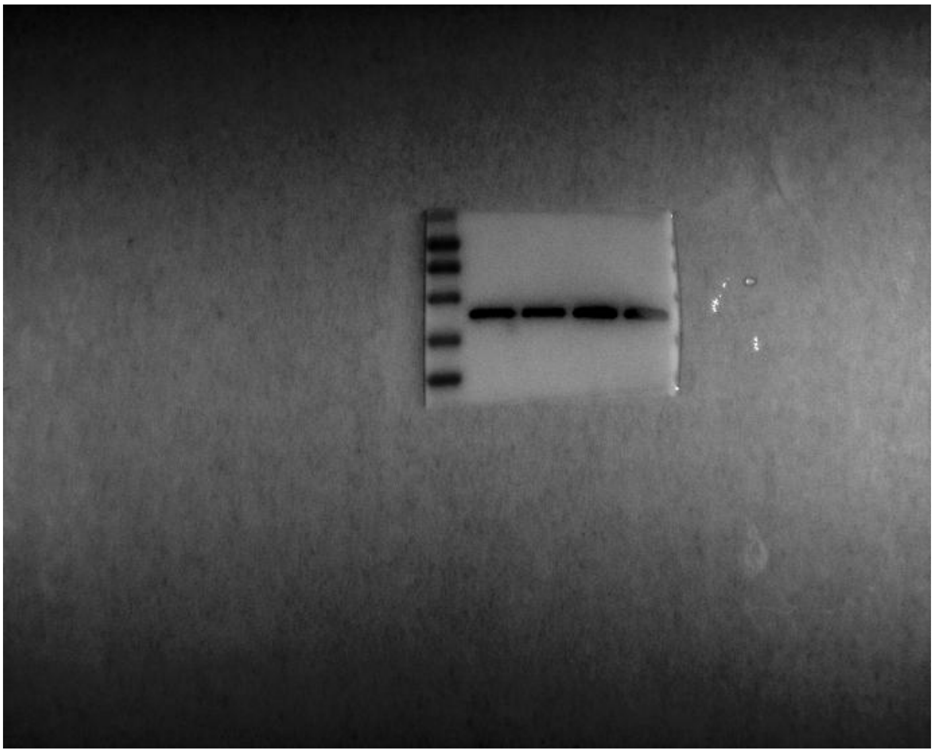

C-cas 3 - 2

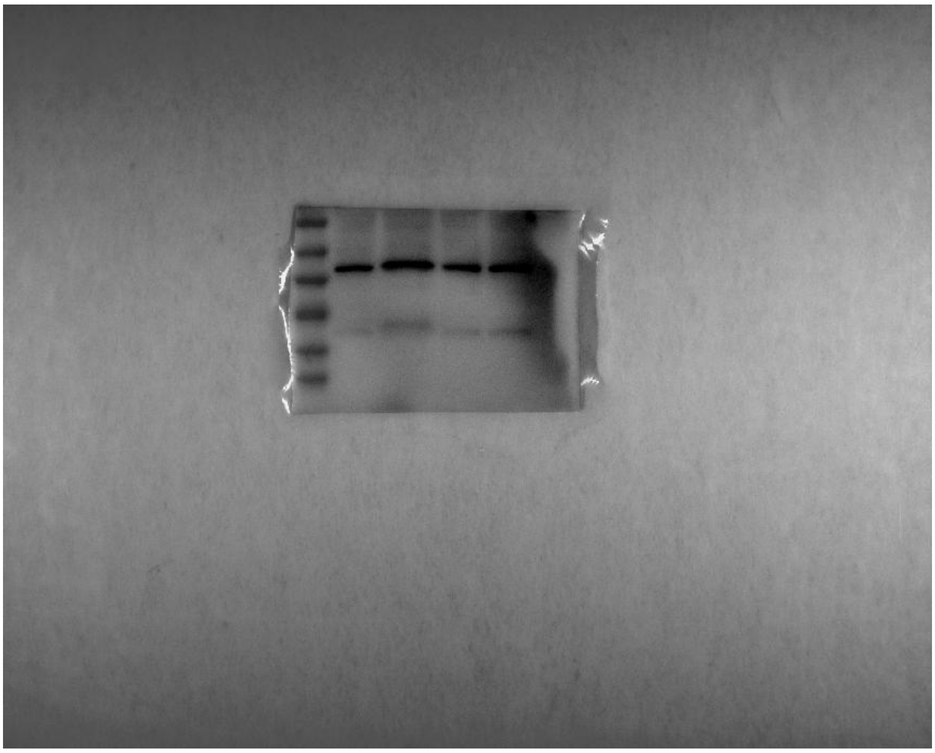

β - actin

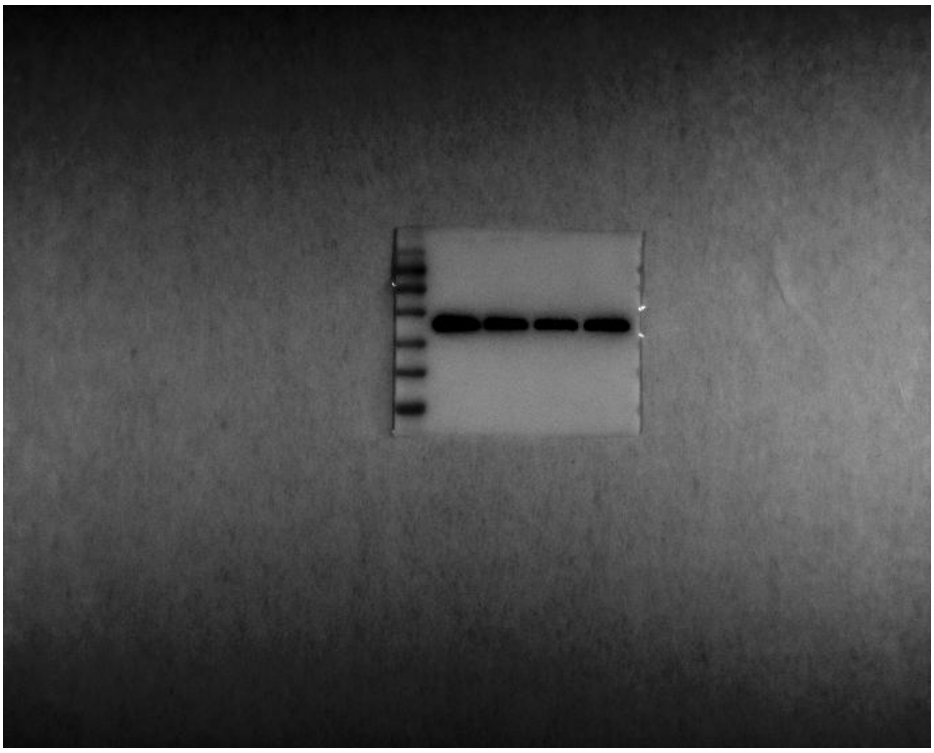

C-cas 3 - 3

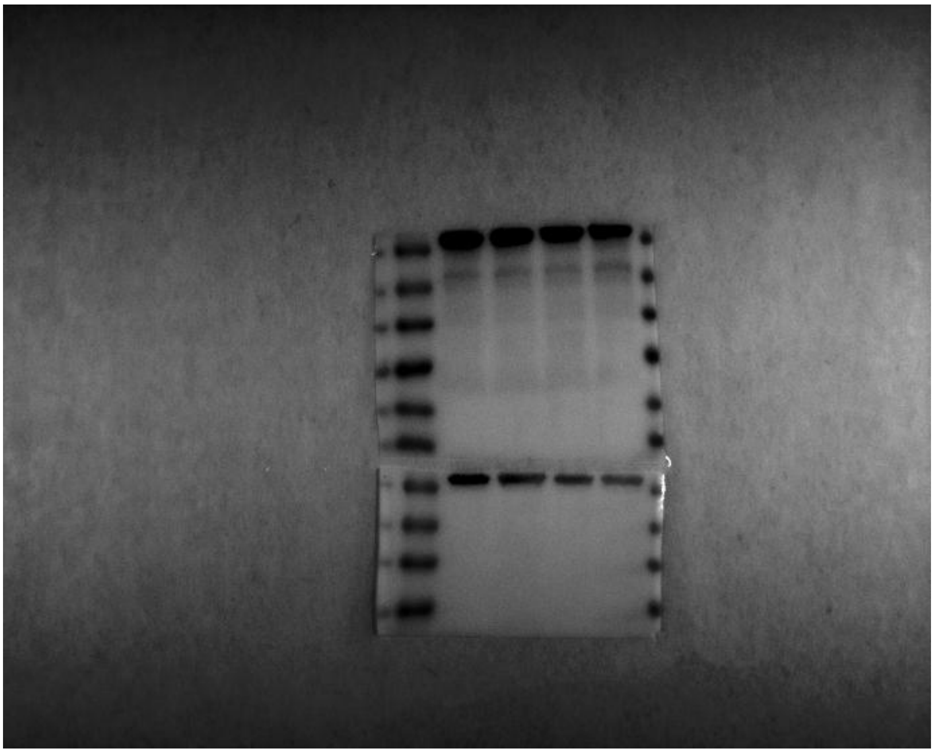

The below blot

β - actin

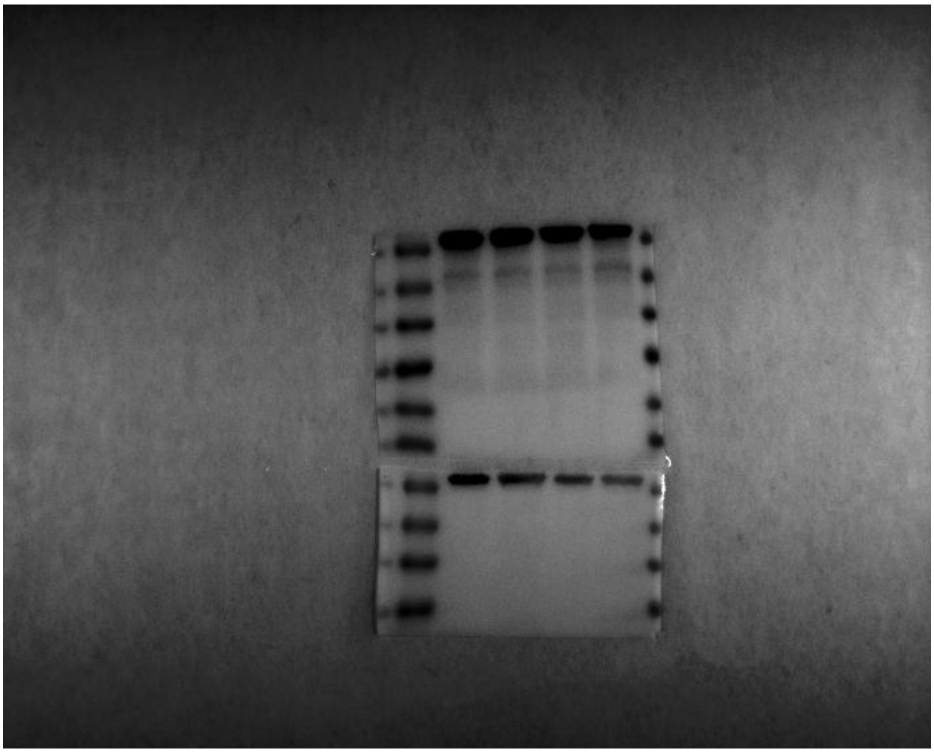

The top blot

C-cas 9 - 1

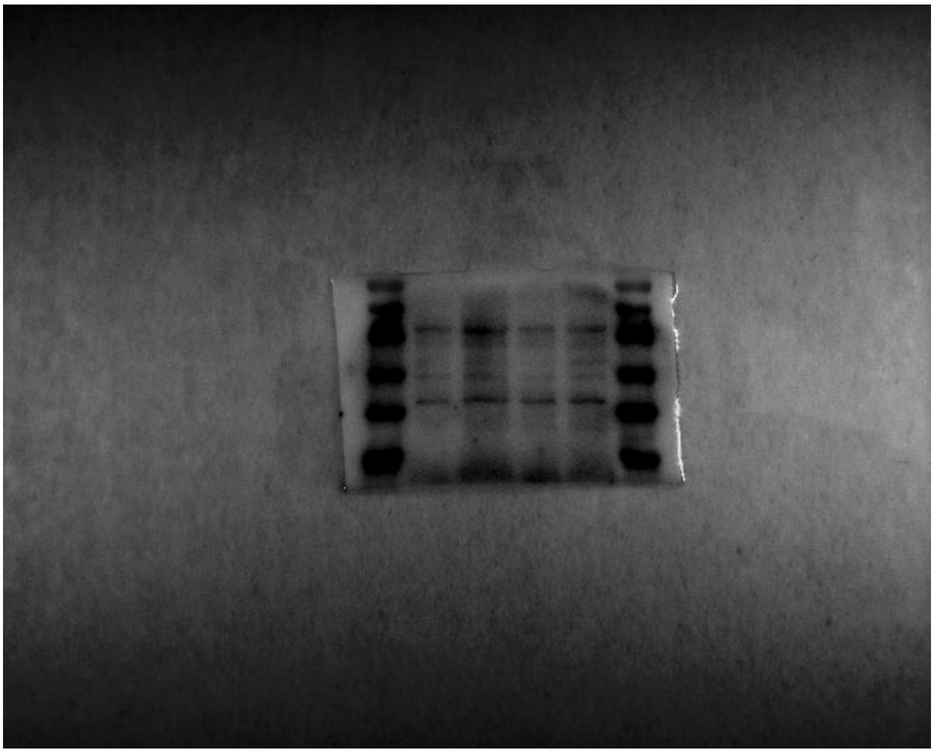

The top blot

β - actin

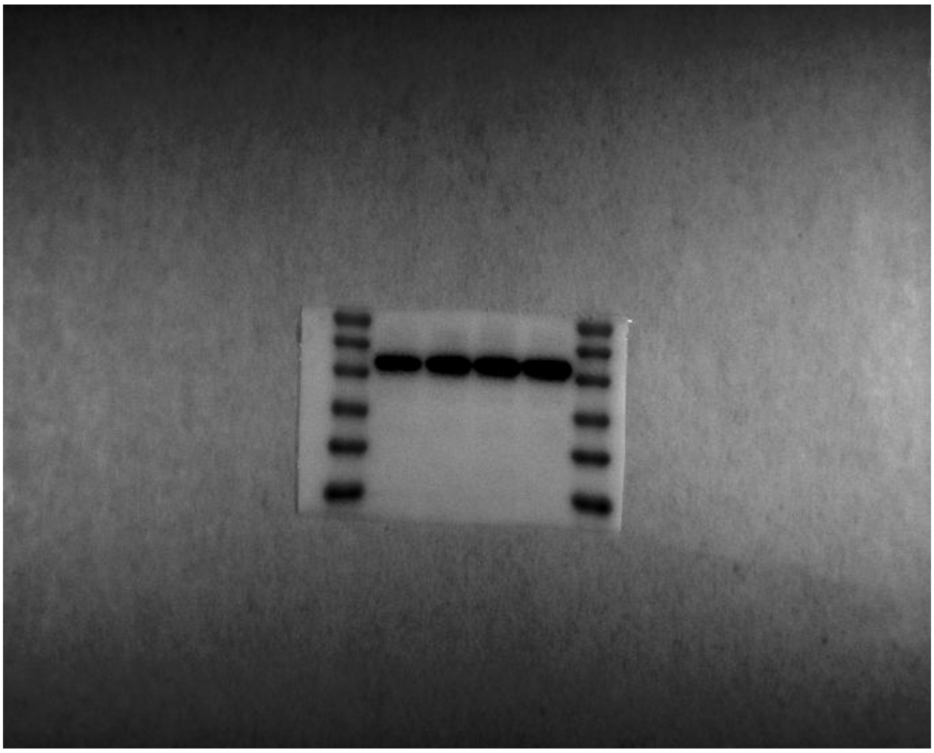

C-cas 9 - 2

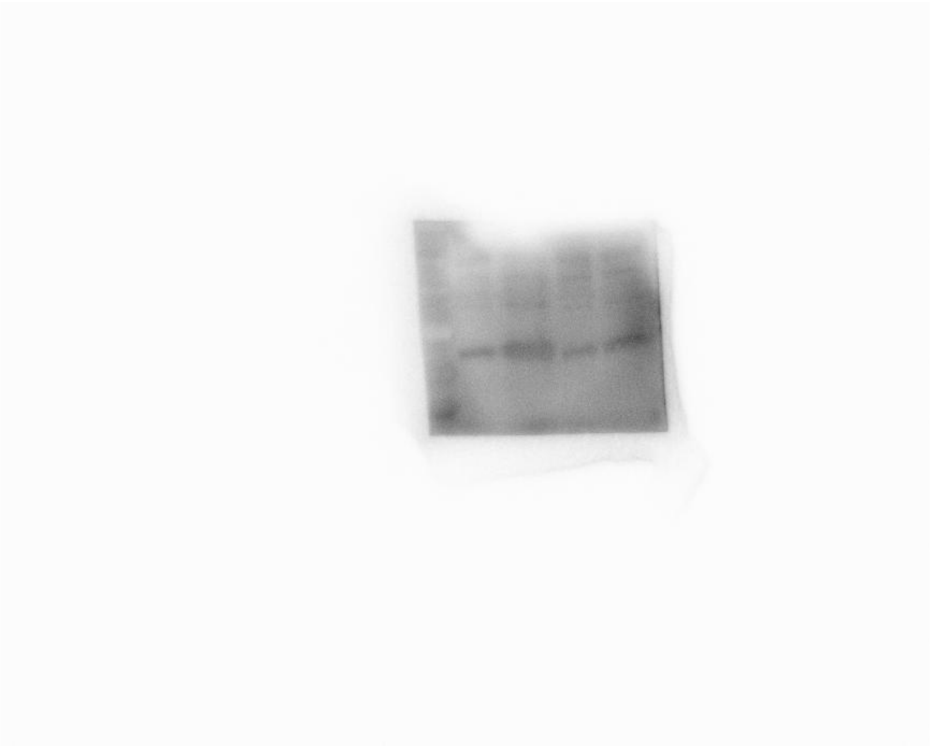

β - actin

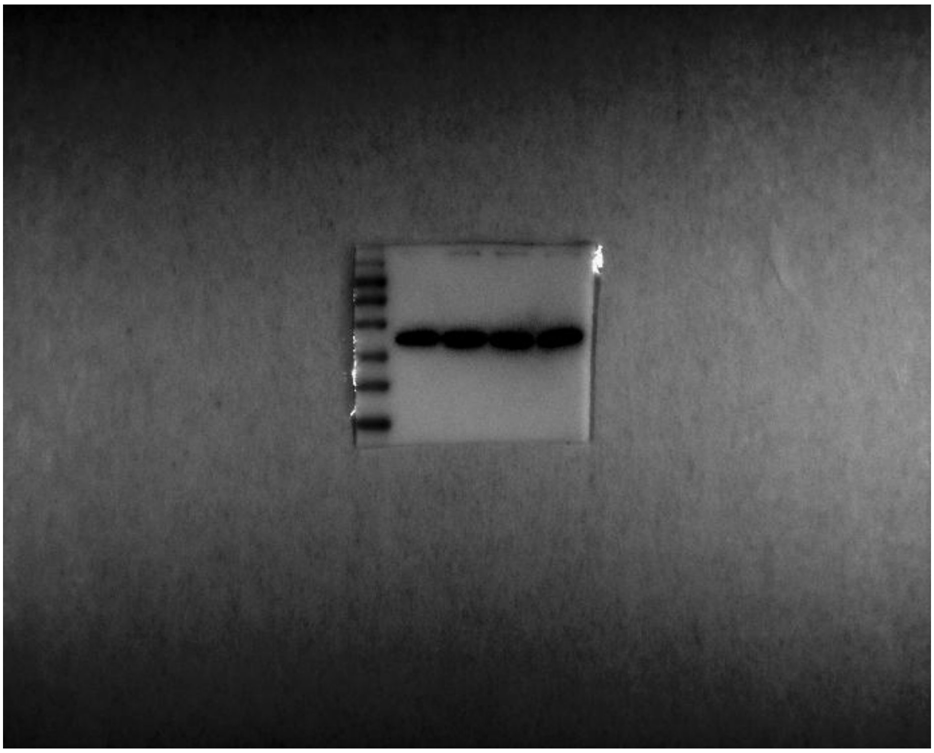

C-cas 9 - 3

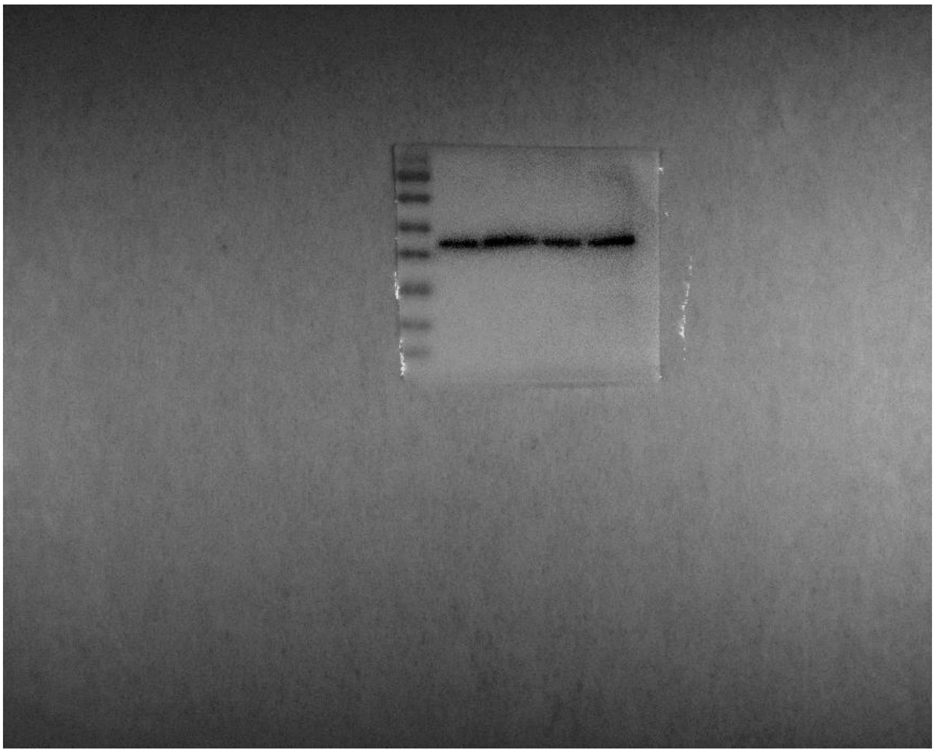

β - actin

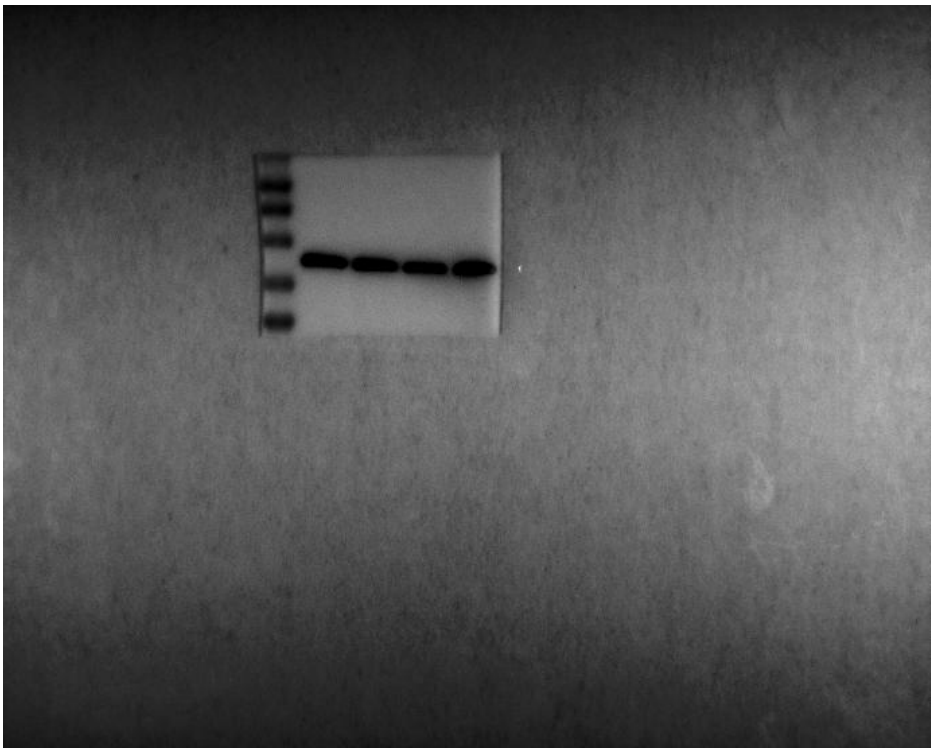

P-53 - 1

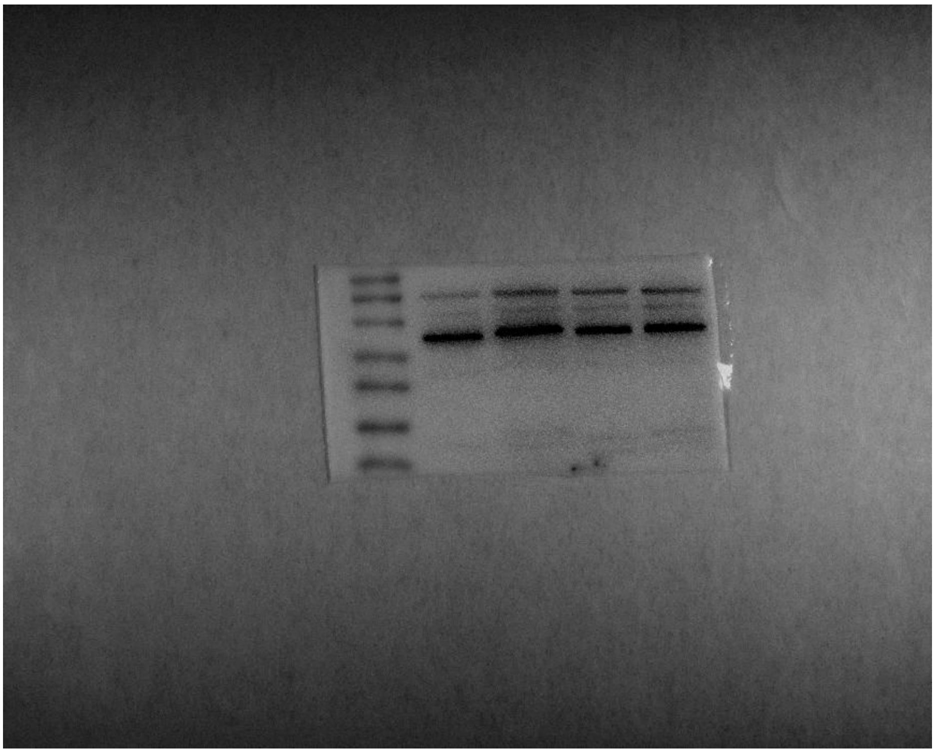

β - actin

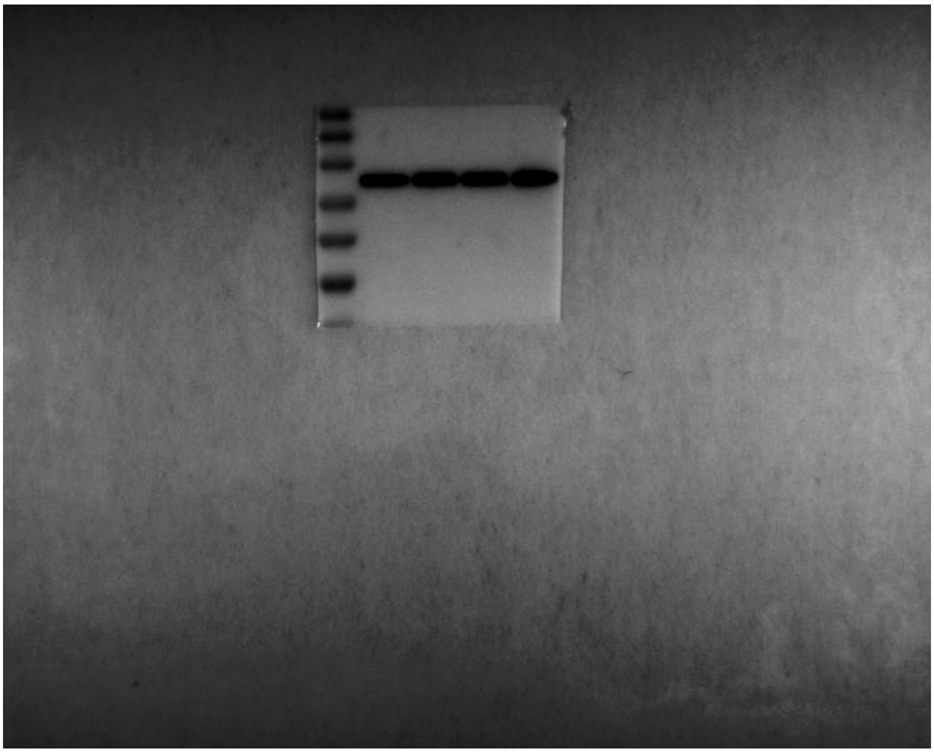

P53 - 2

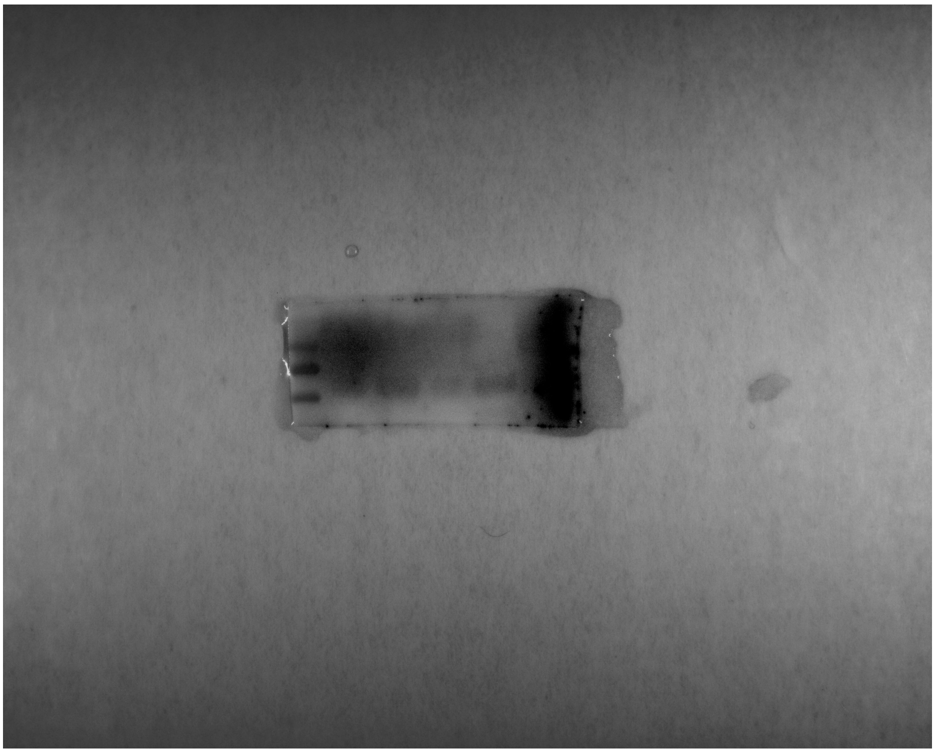

β - actin

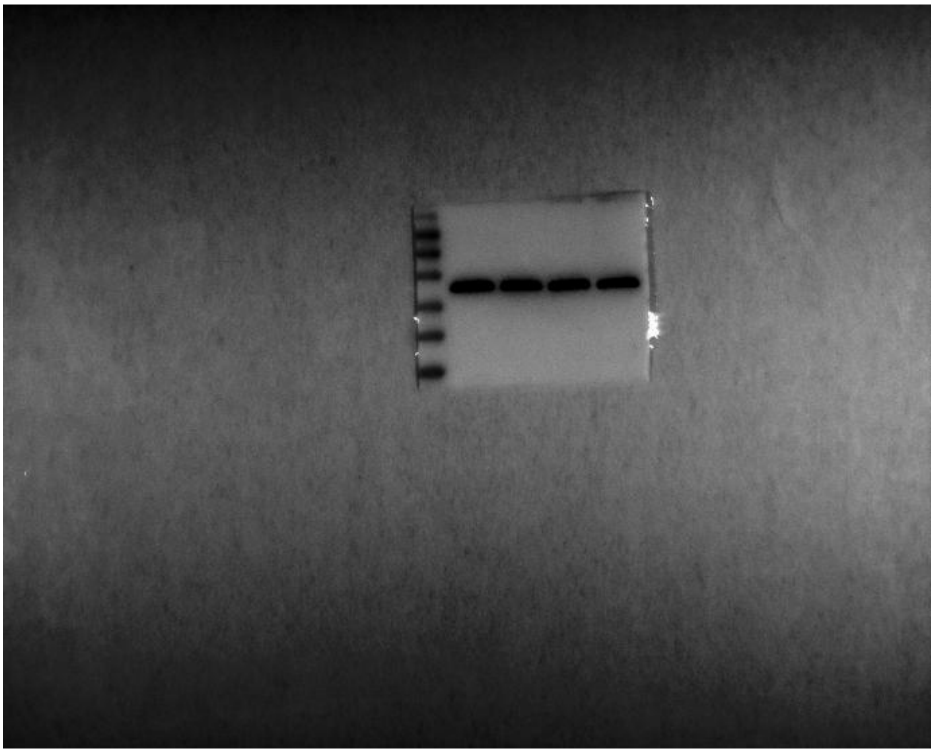

P53 - 3

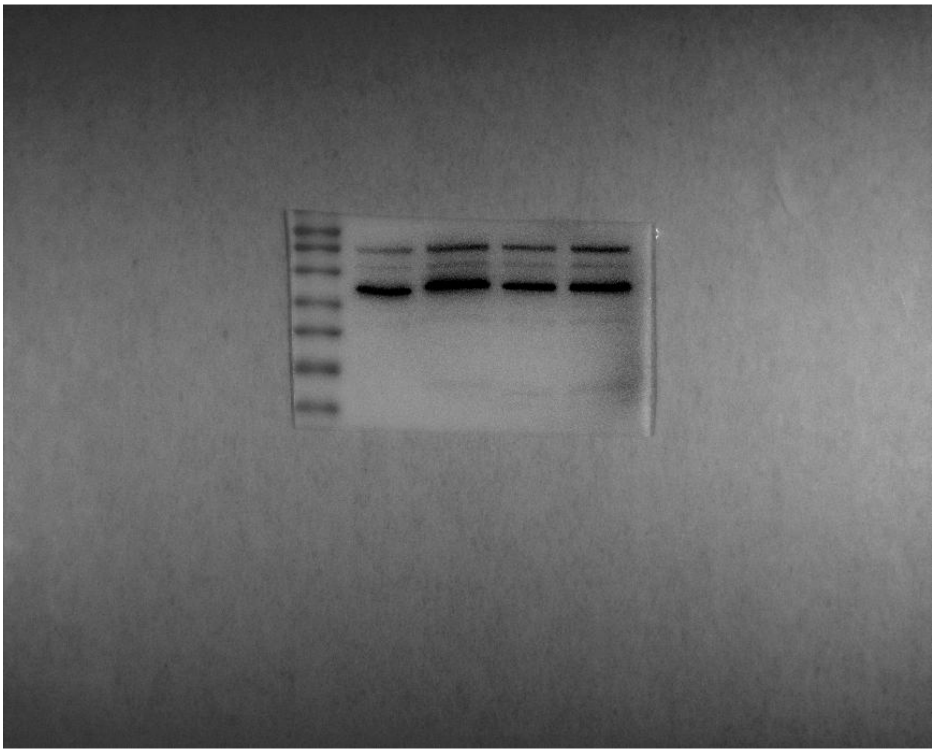

β - actin

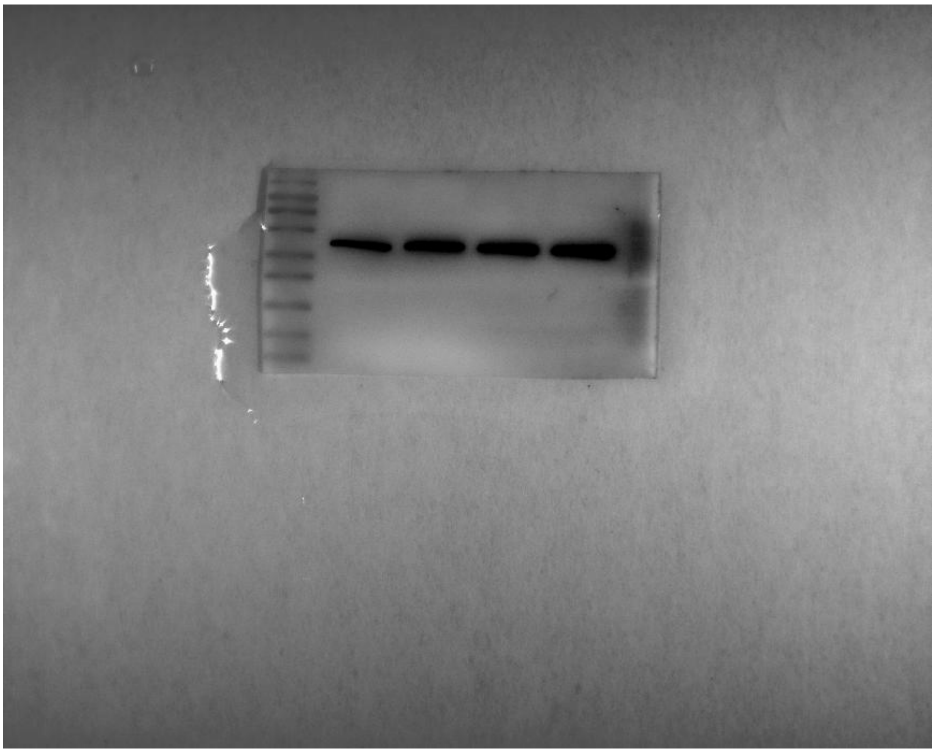

AKT - 1

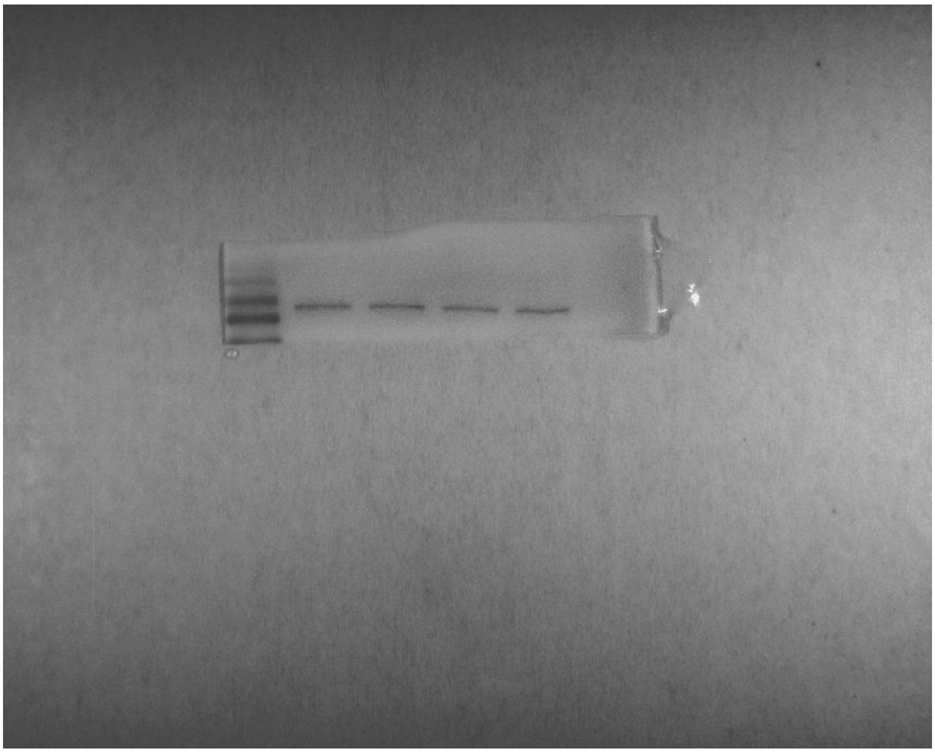

β - actin

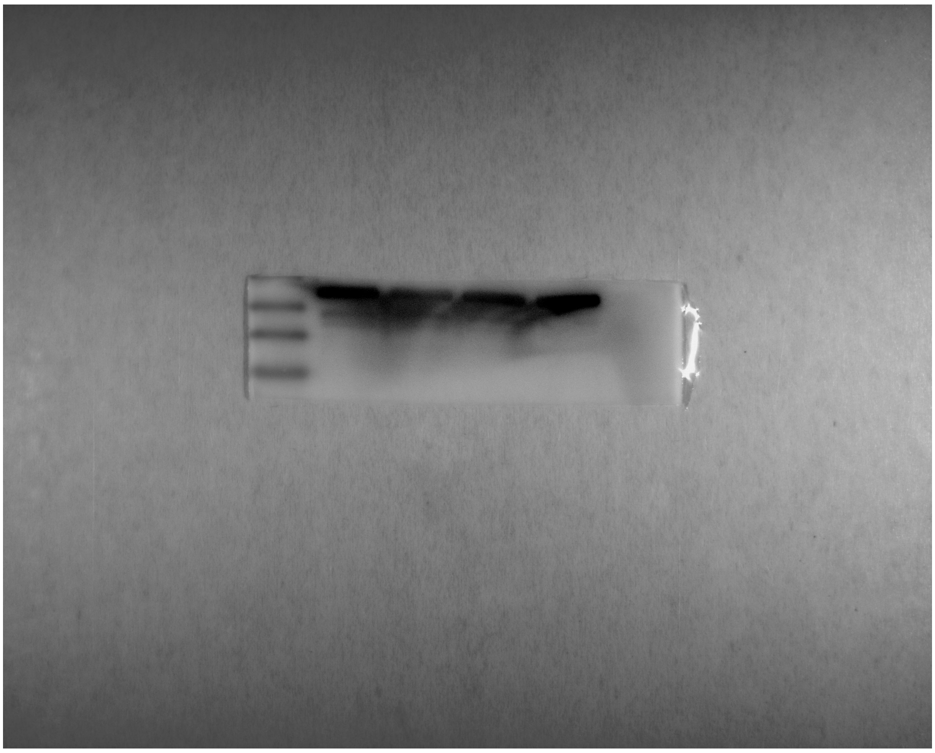

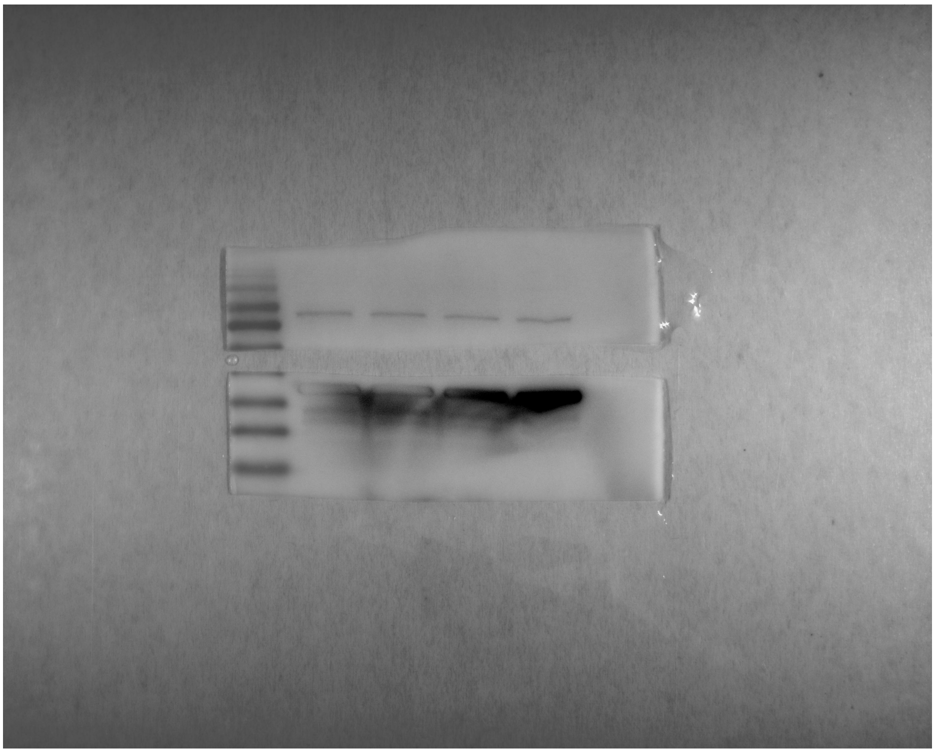

This is the same PVDV, we cut from the middle for imaging

AKT - 2

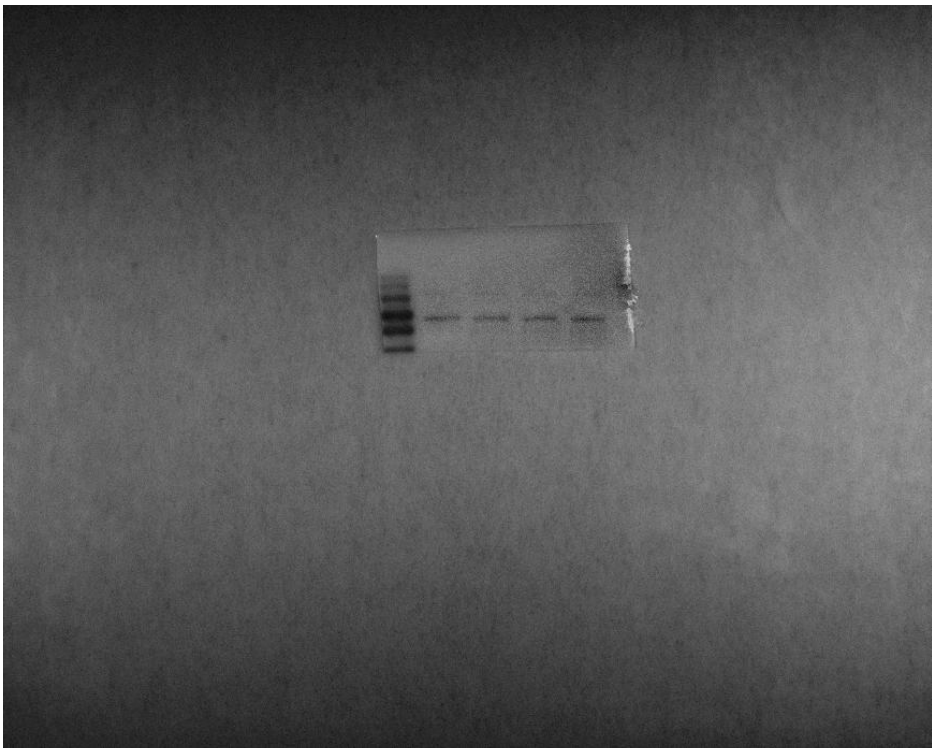

β - actin

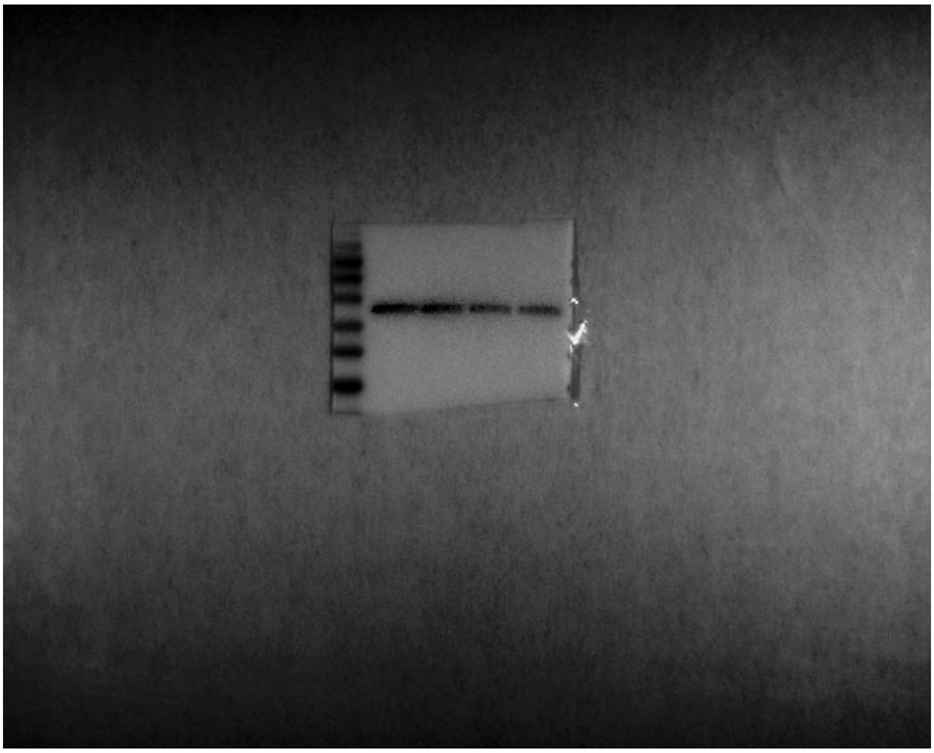

AKT - 3

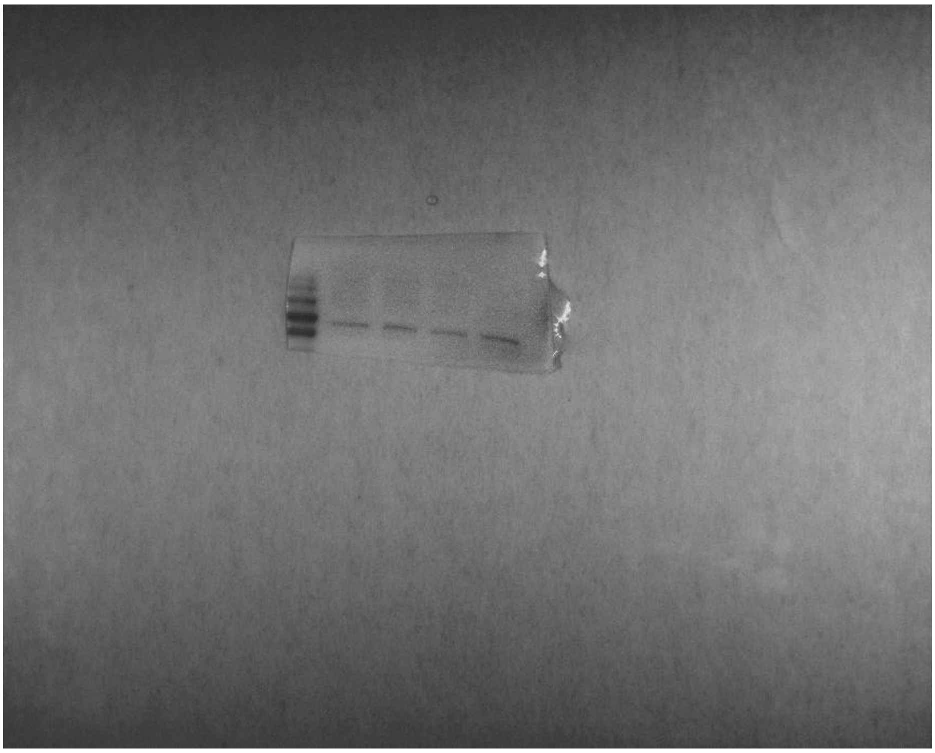

β - actin

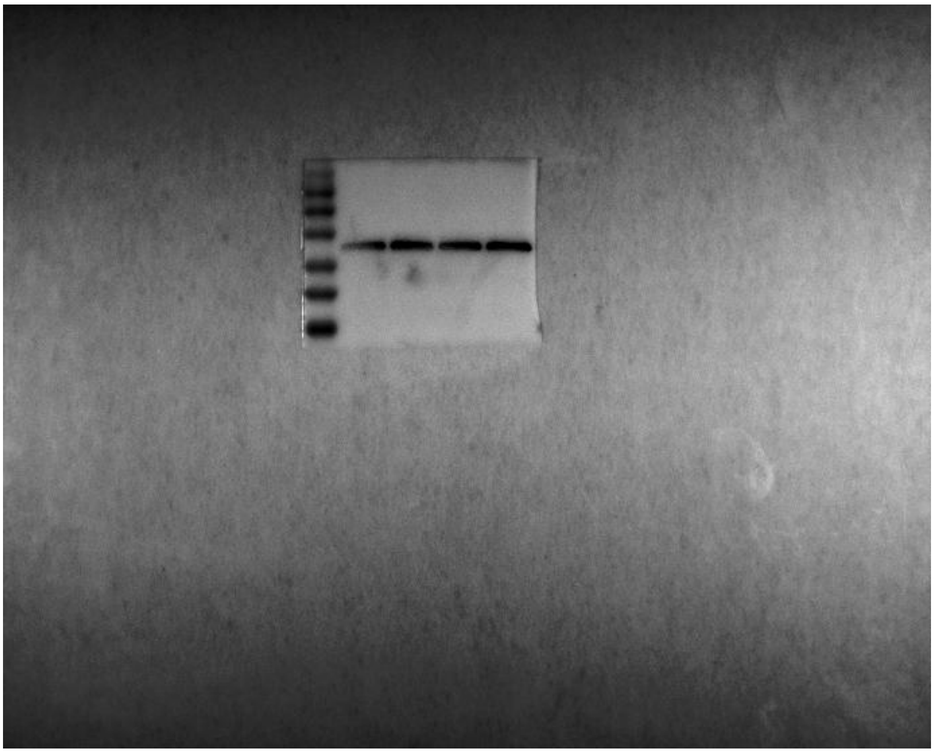

P-AKT - 1

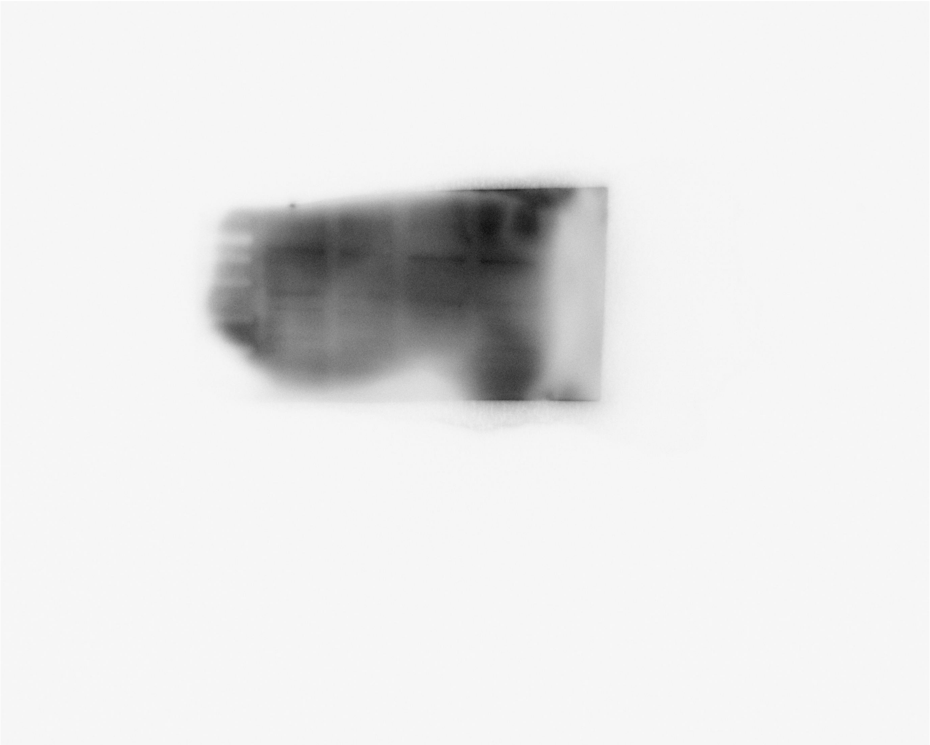

β - actin

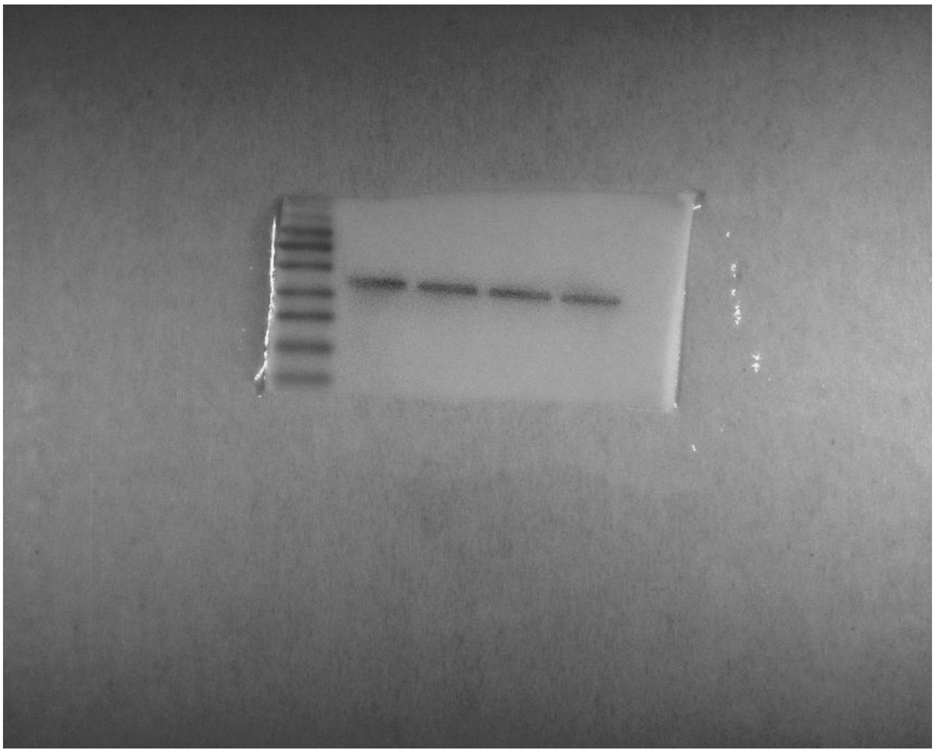

P-AKT - 2

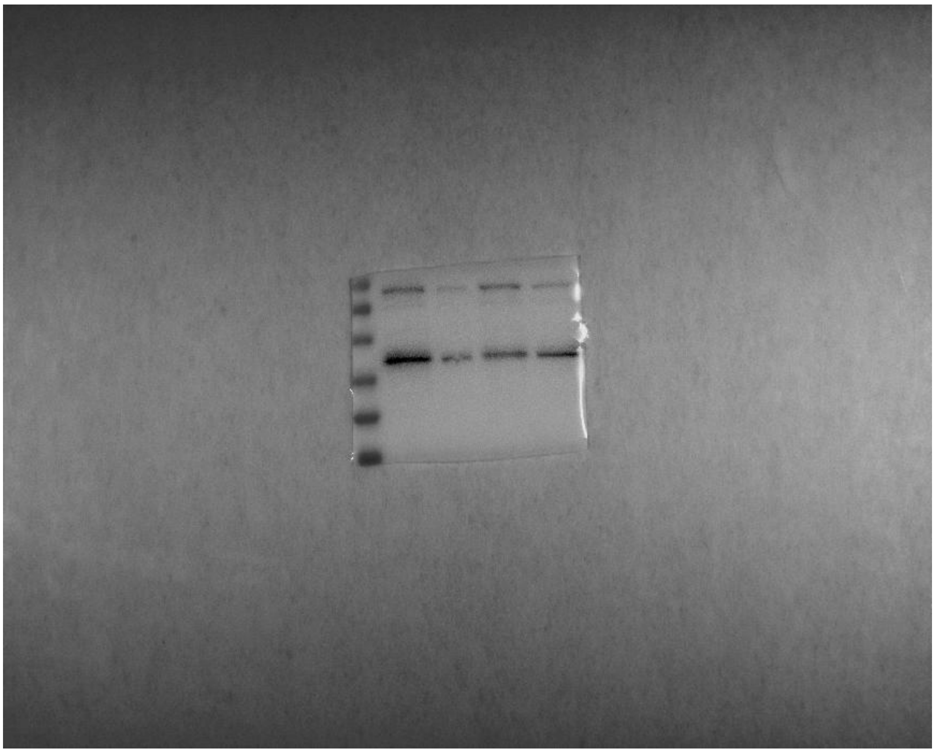

β - actin

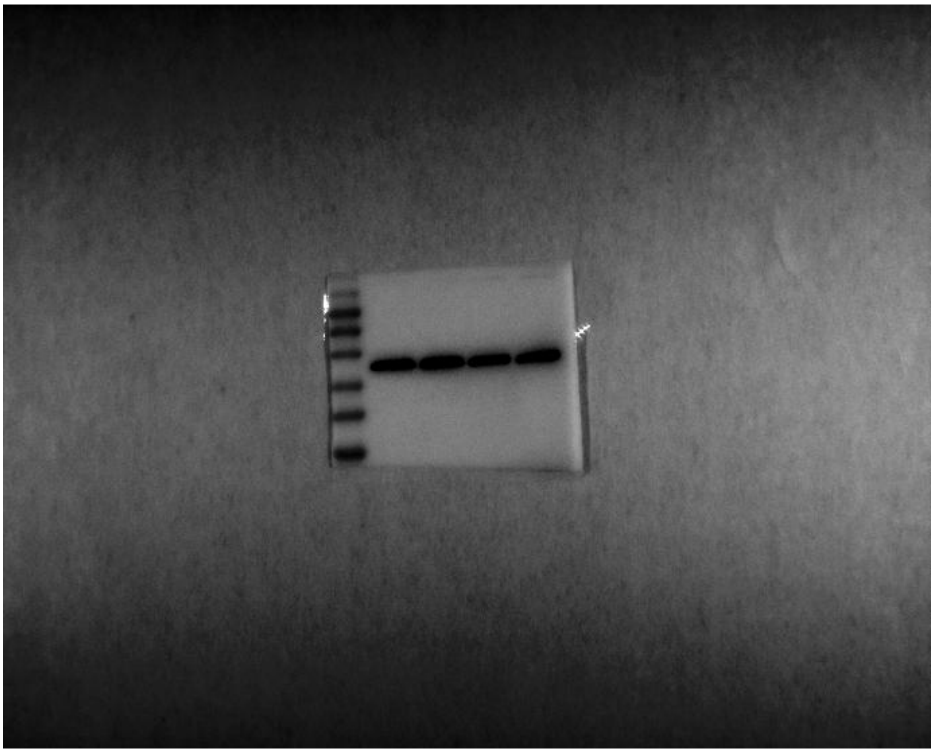

P-AKT -3

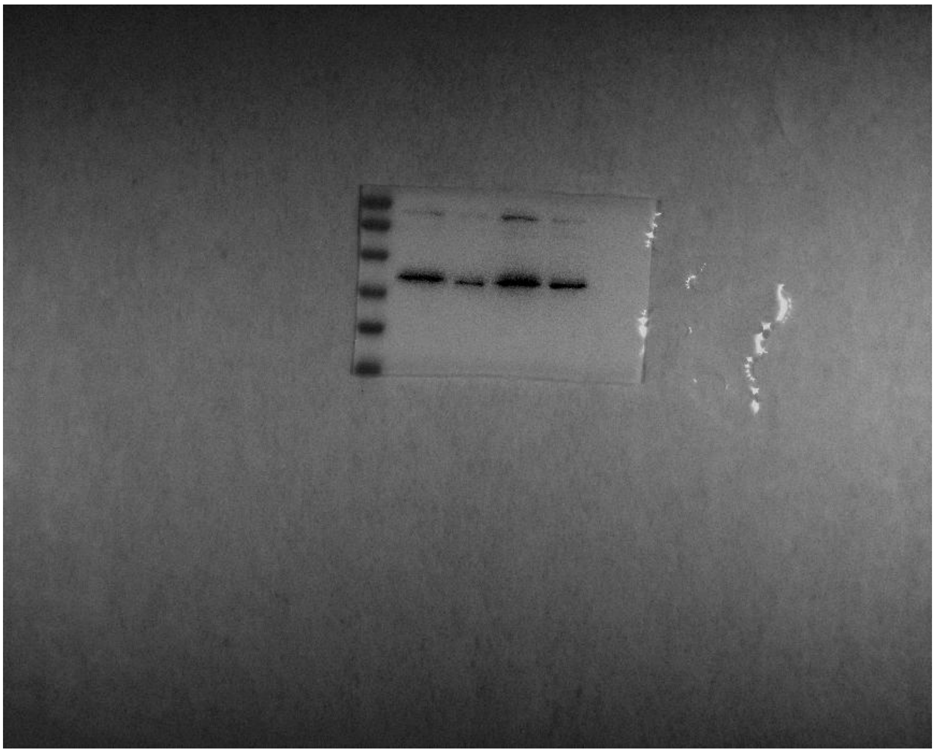

β - actin

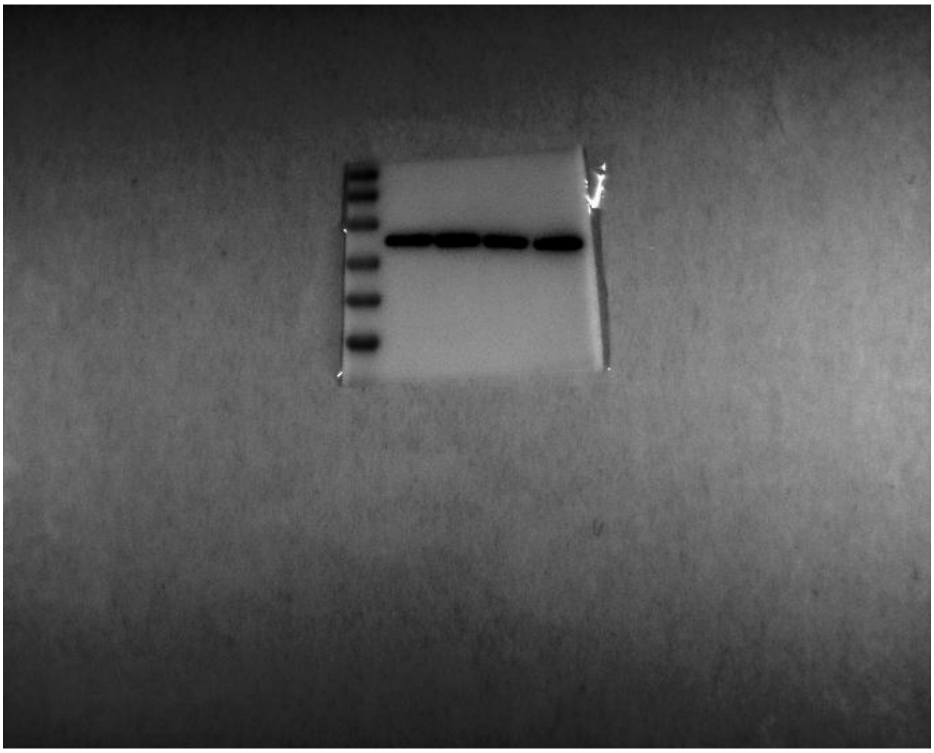

Supplement: Supplementary file 1 [file Datasheet1.pdf]
